# Chemistry of Oxygen Ionosorption on SnO_2_ Surfaces

**DOI:** 10.1021/acsami.1c08236

**Published:** 2021-07-12

**Authors:** Kostiantyn V. Sopiha, Oleksandr I. Malyi, Clas Persson, Ping Wu

**Affiliations:** † Solar Cell Technology, Department of Materials Science and Engineering, Uppsala University, Box 534, SE-75121 Uppsala, Sweden; ‡ Renewable and Sustainable Energy Institute, University of Colorado, Boulder, Colorado 80309, United States; § Centre for Materials Science and Nanotechnology/Department of Physics, University of Oslo, P.O. Box 1048, Blindern, NO-0316 Oslo, Norway; ∥ Division of Applied Materials Physics, Department of Materials Science and Engineering, KTH Royal Institute of Technology, SE-10044 Stockholm, Sweden; ⊥ Entropic Interface Group, Engineering Product Development, Singapore University of Technology and Design, 8 Somapah Road, Singapore 487372, Singapore

**Keywords:** ionosorption model, chemiresistive sensing, tin dioxide, charged oxygen species, surface chemistry

## Abstract

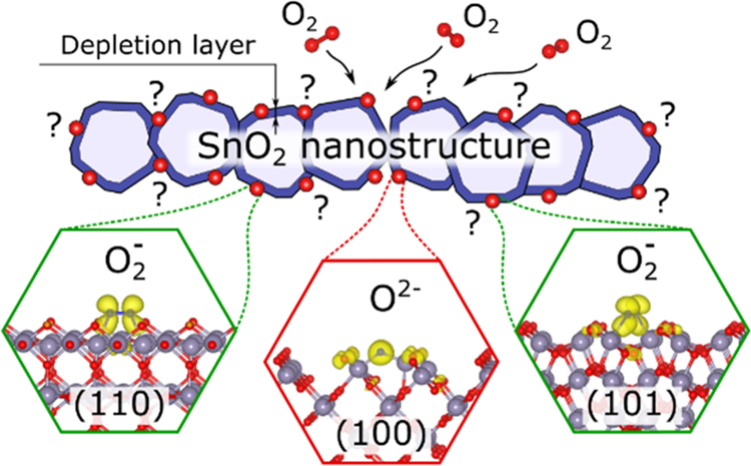

Ionosorbed oxygen is the key player in reactions on metal-oxide surfaces. This is particularly evident for chemiresistive gas sensors, which operate by modulating the conductivity of active materials through the formation/removal of surface O-related acceptors. Strikingly though, the exact type of species behind the sensing response remains obscure even for the most common material systems. The paradigm for *ab initio* modeling to date has been centered around charge-neutral surface species, ignoring the fact that molecular adsorbates are required to ionize to induce the sensing response. Herein, we resolve this inconsistency by carrying out a careful analysis of all charged O-related species on three naturally occurring surfaces of SnO_2_. We reveal that two types of surface acceptors can form spontaneously upon the adsorption of atmospheric oxygen: (i) superoxide O_2_^–^ on the (110) and the (101) surfaces and (ii) doubly ionized O^2–^ on the (100) facet, with the previous experimental evidence pointing to the latter as the source of sensing response. This species has a unique geometry involving a large displacement of surface Sn, forcing it to attain the coordination resembling that of Sn^2+^ in SnO, which seems necessary to stabilize O^2–^ and activate metal-oxide surfaces for gas sensing.

## Introduction

Ever since the oxygen ionosorption model was developed in the 1950s,^1^ it has been a rule-of-thumb explanation for chemiresistive gas-sensing effect.^2−6^ Its basic concept is that oxide surfaces exposed to oxidizing/reducing atmosphere undergo the formation/annihilation of charged oxygen species, which leads to the appearance/removal of the highly resistive surface depletion layer, thereby changing the overall electrical resistance of active material. Beyond any doubt, this mechanism is indispensable for describing gas sensing with single-phase metal oxides. Moreover, it became a foundation for sensing models in more complex material systems, such as p–n composites of various shapes and origins.^3,6^ In these regards, it is surprising how little is known about the exact defects responsible for the sensing behavior of each specific material.^7,8^

The problem is evident for SnO_2_, which is the most successful gas-sensing oxide material to date.^5^ The formation of three different surface acceptors has been suggested upon adsorption of O_2_ molecules on SnO_2_ surfaces: superoxide (O_2_^–^) ions by chemisorption and ionization, as well as singly (O^–^) and doubly (O^2–^) ionized atomic oxygen by the subsequent dissociation.^2,7^ Peroxide (O_2_^2–^) ions are also possible, in principle, but there is very little experimental evidence of their existence on SnO_2_ surfaces.^7^ Since any of these acceptors can rationalize the modulation of the surface depletion layer, phenomenological studies usually do not point out the exact source of sensing response. Most authors choose to speak in favor of O^–^, despite that species has not been observed by direct spectroscopic studies.^7^ Instead, electron paramagnetic resonance (EPR) measurements reveal an abundance of O_2_^–^ on the preliminary reduced surfaces and absence of O^–^ on the oxidized SnO_2_,^9,10^ while also concluding nothing about O^2–^ and O_2_^2–^ due to their diamagnetic nature being invisible to EPR.^4,10^

By combining the EPR results with temperature-programmed desorption (TPD) spectra for SnO_2_, the O_2_^–^ species were shown to escape at about 150 °C without transforming into other forms of ionosorbed oxygen.^11^ Further, exhaustive desorption of unidentified nonmagnetic oxygen (either O^2–^, O_2_^2–^, or lattice oxygen O_lat_^2–^) was observed at about 500 °C. Since the majority of SnO_2_-based chemiresistors operate at 250–350 °C, these unidentified defects must be the ones mediating the sensing response. Nevertheless, other authors claim that O^2–^ and O_2_^2–^ are not stable, and thus, are absent on the SnO_2_ surface.^2,7^ Excluding all four species leaves no suitable candidates for the oxygen ionosorption. Similar contradictions can be identified for other sensing oxides, such as ZnO and TiO_2_, thereby undermining the validity of the ionosorption model, in general.

Throughout the past decades, several works have used first-principles calculations to study the adsorption of neutral O adatoms and O_2_ molecules on the most stable surfaces of the common sensing oxides (SnO_2_, TiO_2_, and ZnO).^12−18^ Contrary to the initial expectations, they concluded that O adsorption on the fully oxidized surfaces via dissociation of atmospheric oxygen is unfavorable. Moreover, O_2_ molecules were found to undergo only weak physisorption, suggesting that neutral oxygen is not present in any form under the standard operating conditions. This is not particularly critical for gas sensing though, as the ionosorption model requires surface species to be charged, simply because the formation of neutral surface species would not alter carrier concentrations. Despite these concerns, several authors still analyzed interactions between surfaces containing the unstable surface oxygen and other gas molecules.^19−22^ However, the applicability of these results to the description of the gas-sensing reactions is questionable.

The first effort to investigate charged oxygen defects on SnO_2_ surfaces was made by Golovanov et al.^23,24^ The authors demonstrated that the presence of extra electrons or fluorine doping in SnO_2_(110) slab stabilizes certain O and O_2_ adsorption positions and provides them with an extra negative charge. Unfortunately, because the slab had arbitrary dimensions and since only default computational parameters were employed, those pioneering works could not identify any stable configurations and did not converge properties of the ionosorption species. A similar tendency for the formation of superoxide O_2_^–^ upon the molecular adsorption was later shown for Al-doped ZnO^13^ and defective anatase TiO_2_,^25,26^ where the adsorbing oxygen trapped electrons generated by (sub-)surface defects. The complex interplay between these defects, however, does not allow us to determine the true chemistry of the surface species. The desire to explain the sensing phenomenon has even led many authors into modeling adsorption of O and O_2_ on reduced surfaces.^12,14,18,24,27−29^ This approach, however, ignores the fact that reduced SnO_2_ surfaces^30^ and even individual surface oxygen vacancies^31^ are highly unstable under the O-rich conditions of atmospheric exposure. Naturally, the presence of the unstable oxygen vacancies promoted chemisorption, increased change transfer to the analyte, and promoted dissociation of the adsorbing molecules. However, the achieved stabilization was primarily due to irreversible healing of the surface, which is incompatible with the repeatability of sensing reactions in experiments, or, to a lesser extent, due to trapping of electrons introduced by the unstable surface oxygen vacancies.

The discrepancies between experimental findings, first-principles analyses, and basic ionosorption model reflect an evident lack of knowledge on surface defect chemistry of oxides. In this regard, SnO_2_ is only a conspicuous example but not an exceptional case. Motivated by the desire to fulfill this gap, we perform the first detailed first-principles investigation of oxygen-related defects on SnO_2_ surfaces with the focus on charged species. Most calculations herein are carried out using PBE+*U* scheme with the carefully selected Hubbard *U* value to reproduce the position of the conduction band minimum and to effectively correct for the spurious electrostatic interactions in periodic cells. We believe that the gained knowledge will provide a deeper understanding of the surface chemistry of oxides. Not only will it help to advance gas sensors, which is the primary discussion point for this study, but also stimulate the development of new routes for semiconductor fabrication, promote the rational design of adhesives and catalysts, and beyond.

## Results and Discussion

### Surface States and Band Bending

**Figure 1:**
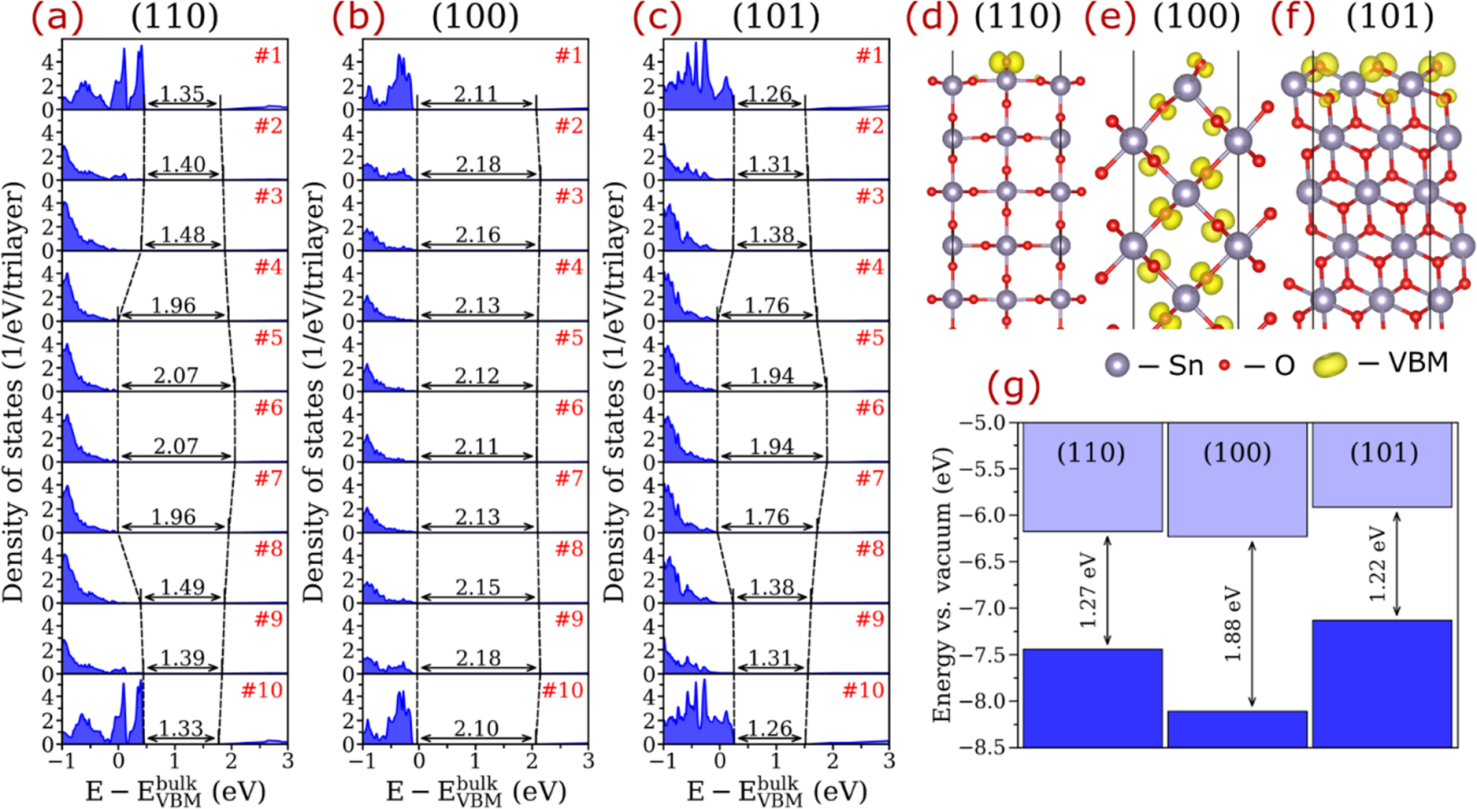
Electronic structures of the clean 10-trilayer-thick SnO_2_ slabs. (a–c) Layer-resolved local density of states (LDOS); the black numbers represent effective band gap energies computed from the layer-resolved LDOS neglecting the population densities below 0.1 1/eV/trilayer. (d–f) Charge density distribution at valence band maximum (VBM) for the slab systems. (g) Band edges with respect to the vacuum level for the surfaces.

Since a vast majority of gas sensors are based on either nanostructured or polycrystalline SnO_2_, careful investigation of all naturally grown surfaces is necessary. Experimentally, it has been established that surfaces of as-synthesized SnO_2_ are dominated by (110), (100), and (101) facets.^32^ This is not surprising considering that these crystal terminations have the lowest surface energies,^27,30^ with the values computed herein being 1.21, 1.27, and 1.60 J/m^2^, respectively. Despite similar energies, their electronic properties are considerably different, as illustrated in Figure 1. Specifically, the computed band gaps for the six-trilayer-thick (110) and (101) slabs are 1.27 and 1.16 eV, respectively, which is considerably lower compared to the bulk value (1.75 eV). The difference is due to the emergence of surface states with O 2p-like character above valence band maximum (VBM) of bulk SnO_2_. We have recently observed and characterized similar states at ABO_3_(001) perovskite surfaces,^33,34^ which therefore seems to be a common feature for oxides. In contrast, no formation of surface states was found on the clean (100) facet, which yielded an energy gap of 1.98 eV for the six-trilayer-thick system. As one can notice, this value is 0.23 eV larger than that for the bulk due to a long-range band bending.^35^ This effect also manifests itself as a thickness-dependent energy gap of the SnO_2_ slabs, as shown in Figure S3. Both surface states and band bending introduce VBM shifts for the slab systems, which are important to account for in the defect energy calculations, as discussed in the Methods section.

### Molecular Oxygen Species

**Figure 2:**
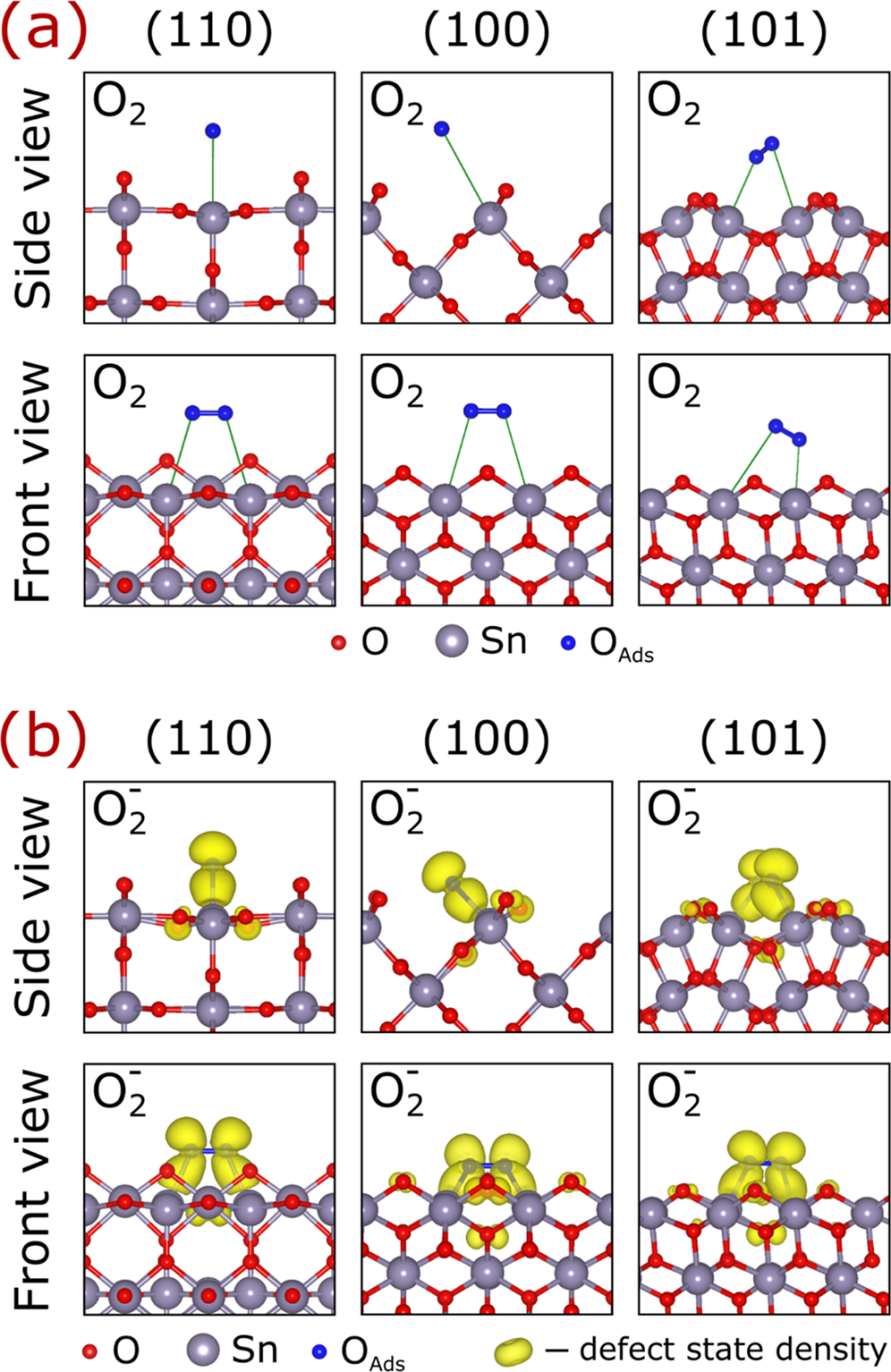
Adsorption configurations for molecular oxygen on SnO_2_ surfaces. (a) Physisorption of neutral O_2_ molecules and (b) chemisorption of superoxide O_2_^–^ ions. The defect state density depicts the highest occupied antibonding π* orbital in the band gap. The structures in (b) are provided in the Supporting Information.

**Figure 3:**
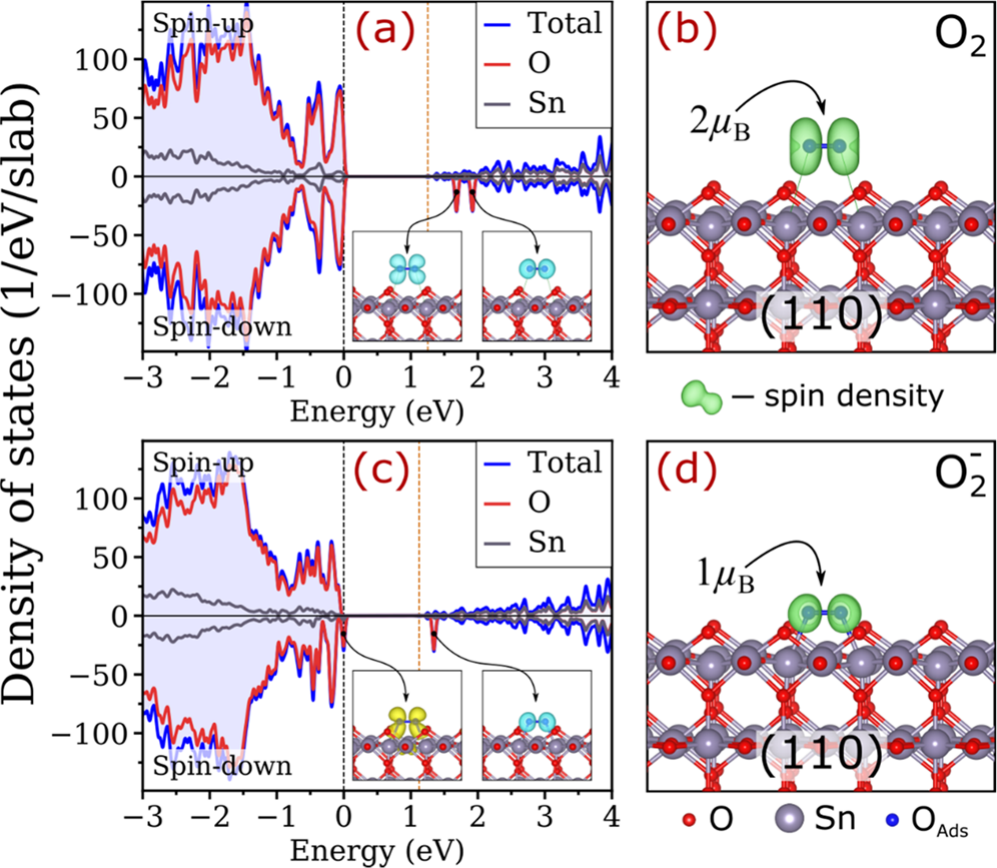
Analysis of the defect states for O_2_ adsorbing on the SnO_2_(110) surface in different charged states. (a, c) Element- and spin-resolved density of states (DOS) and (b, d) spin densities for the species on the slab containing (a, b) zero and (c, d) one extra electron. The black and orange vertical dashed lines in (a) and (c) indicate the highest occupied state and principal CBM of the slab, respectively. The insets in (a) and (c) illustrate change densities projected on the defect states (yellow and blue iso-surfaces denote filled and vacant defect states, respectively).

The screening protocol applied to O_2_ adsorption on all naturally occurring SnO_2_ facets (see the Methods section) revealed that the neutral molecules do not form chemical bonds with the surfaces, in agreement with the literature.^12^ Even for the most stable positions, O_2_ molecules maintain their O–O bond length of 1.23 Å and magnetic moments of 2 μ_B_, while keeping at least 2.78 Å distance to the corresponding surfaces (see Figure 2a and Table 1). Two antibonding π* orbitals of the O_2_ molecules appear above the conduction band minimum (CBM) level of the SnO_2_ slabs (see Figure 3a, for example), but they always remain vacant. All of these results suggest that a neutral physisorbed O_2_ molecule can only serve as an intermediate stage of ionosorption and does not influence the conductivity of SnO_2_ in any significant way.

**Table 1:** Computed Properties of O_2_ Molecules Adsorbed on the SnO_2_ Surfaces

SnO_2_ facet	number of extra electrons	Bader charge on O_2_ molecule (*e*)	magnetic moment (μ_B_)	O–O bond length (Å)	minimum Sn–O bond length (Å)
(110)	0	0.00	2	1.23	3.43
	1	0.58	1	1.33	2.27
(100)	0	0.00	2	1.23	3.96
	1	0.59	1	1.33	2.28
(101)	0	0.00	2	1.23	2.78
	1	0.57	1	1.33	2.23

A very different behavior is observed for O_2_ molecules on SnO_2_ containing one extra electron. In this case, the screening protocol yielded one adsorption position with apparent chemical bonding for each termination. The chemisorption is evident from the formation of two separate Sn–O bonds of 2.23–2.28 Å between each O atom of the molecules and surface Sn atoms, as shown in Figure 2b. Moreover, elongation of the original O–O bonds from 1.23 to 1.33 Å and change of the magnetic moments from 2 to 1 μ_B_ upon adsorption signify the stabilization of superoxide ions (O_2_^–^). Indeed, the superoxide nature of the adsorbed species is confirmed by Bader charge analysis revealing 0.57*e*–0.59*e* transfer to the molecules upon adsorption (see Table 1), which is about a half of that on O atoms in bulk SnO_2_ (1.12*e*). From the electronic perspective, the formation of O_2_^–^ is characterized by energetically shifting down one antibonding π* orbital and filling it with one electron (see densities of states (DOS) for the (110) facet in Figure 3 and the (101) facet in Figure S5). When hybridized via interaction with the surface, these orbitals are equivalent to defect states. Naturally, the occupied defect states are localized on the chemisorbed molecules (see Figure 2b), thus serving as traps for electrons from the conduction band, as observed. The associated changes in spin densities exemplified in Figures 3 and S5 are also consistent with the formation of superoxide species.

Addition of the second electron into the systems containing O_2_^–^ ion alters neither geometry nor electronic properties of the defect. It is not surprising as all subsequently added electrons occupy the conduction band of the host material. Although some partial filling of the defect state above the CBM was observed, that only occurred at unreasonably high concentration of conducting electrons in small slabs. Hence, it can be concluded that molecular oxygen does not adsorb on any naturally grown SnO_2_ surface in the doubly ionized (peroxide) state (O_2_^2–^).

### Atomic Oxygen Species

The screening protocol applied to neutral O adatoms yielded four principal adsorption configurations for each termination. The word “principal” here is used to highlight that the adsorption positions represent the most stable conformation within a larger group of chemisorption configurations with close geometries and electronic properties. Depending on the local bonding and electronic structure, these four principal configurations can be combined into two categories named peroxide-like and lattice-like.

### Peroxide-like Defects

**Figure 4:**
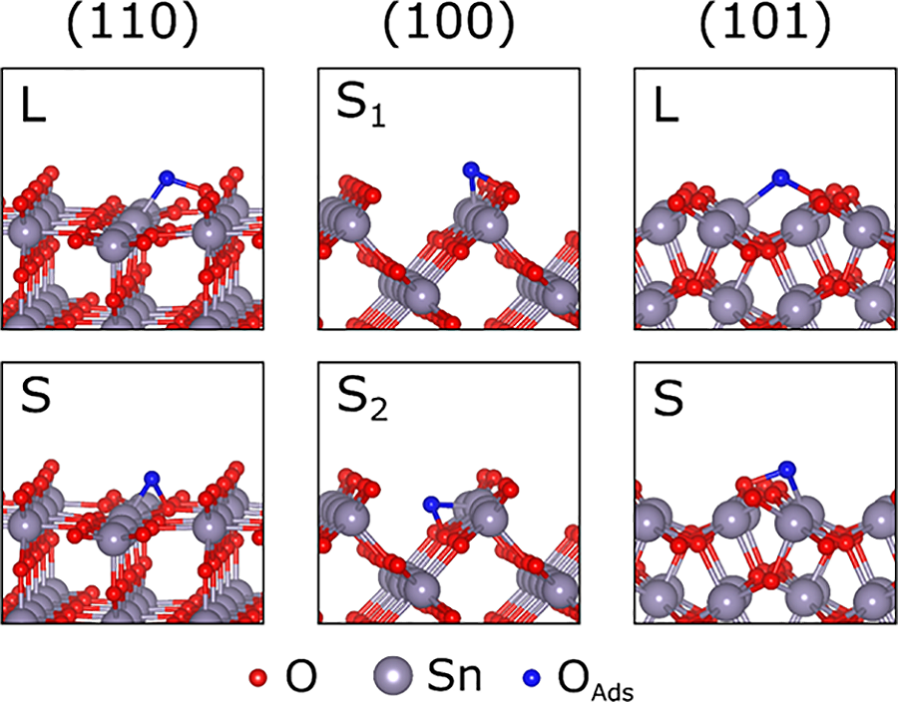
Peroxide-like O adsorption configurations on the SnO_2_ surfaces. The structures are provided in the Supporting Information. The "L" and "S" labels indicate the link- and side-type adsorption positions, respectively.

Peroxide-like defects are distinguished by an O adatom forming one chemical peroxide bond with one O atom at the surface, as illustrated in Figure 4. The formed O–O bond is typified as peroxide if its length is within the 1.48–1.54 Å range, which is quintessential for peroxide ions. Noteworthy, such coordination is typical for O interstitials in bulk SnO_2_ and many other oxides alike.^36,37^ Two types of local bonding can be distinguished for these defects. The first type labeled “S” (side) is recognized by both O atoms of the peroxide complex bonding to the same Sn atom. The second type labeled “L” (link) is characterized by the peroxide bond linking two neighboring Sn atoms. Thus, one S-type and one L-type conformation were found on (110) and (101) facets, while two different S-type defects (labeled S_1_ and S_2_) were observed on SnO_2_(100). Regardless of the local bonding, all peroxide-like conformations share two key characteristics: (i) zero magnetic moment, which is typical for peroxides; and (ii) 1.10*e*–1.26*e* net Bader charge on the peroxide group (see Table 2), which is close to that on O atom in bulk SnO_2_ (1.12*e*). These properties justify the peroxide-like classification of these species.

**Table 2:** Computed Properties of O Adatoms on the SnO_2_ Surfaces in the Peroxide-like Configurations

SnO_2_ facet	adsorption position	number of extra electrons	net Bader charge on O_2_ unit (*e*)	magnetic moment (μ_B_)	O–O bond length (Å)
(110)	L	0	1.18	0	1.49
	S	0	1.24	0	1.53
(100)	S_1_	0	1.18	0	1.54
	S_2_	0	1.26	0	1.50
(101)	L	0	1.17	0	1.48
	S	0	1.10	0	1.48

Another common feature of the peroxide-like configurations is that the addition of extra electrons into the slab does not change any of their properties. Instead, the introduced electrons occupy the conduction band as if the slabs had no surface adsorption species at all. This behavior can be rationalized by treating a peroxide-like defect as O_2_^2–^ ion replacing lattice oxygen (O_lat_^2–^). The isovalent character of such substitution explains why these species behave as neutral defects. Hence, the formation of peroxide-like defects is also irrelevant for the chemiresistive effect because it provides no mechanism to alter concentration of charge carriers.

### Lattice-like O Ionosorption Defects

**Figure 5:**
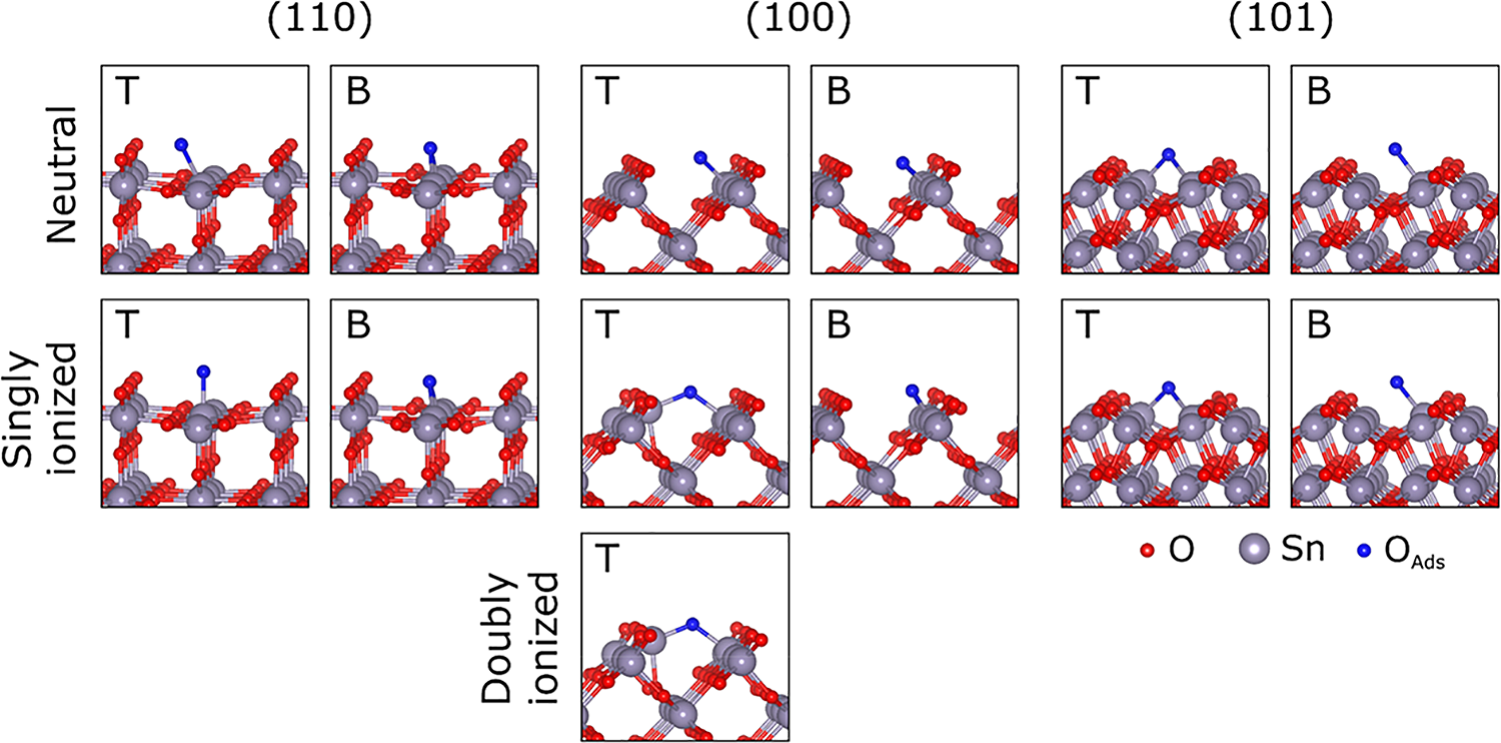
Lattice-like O adsorption configurations on the SnO_2_ surfaces. The "T" and "B" labels indicate the top- and bridge-type adsorption positions, respectively.

**Figure 6:**
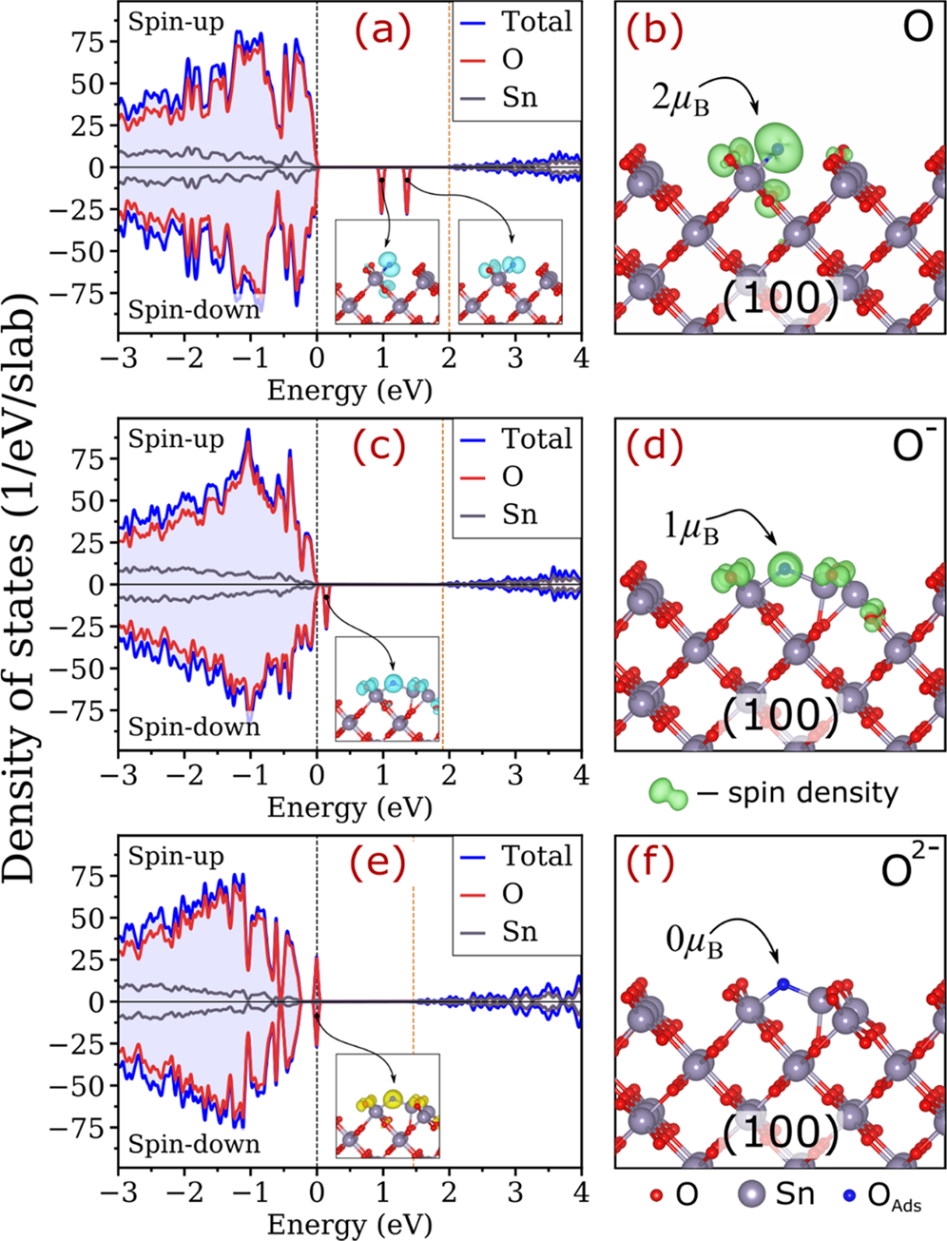
Analysis of the defect states for the T-type O adsorption species on the SnO_2_(100) surface in different charged states. (a, c, e) Element- and spin-resolved DOS and (b, d, f) spin densities for the defect species on the slab containing (a, b) zero, (c, d) one, and (e, f) two extra electrons. The black and orange vertical dashed lines in (a), (c), and (e) indicate the highest occupied state and principal CBM of the slab, respectively. The insets in (a), (c), and (e) illustrate change densities projected on the defect states (yellow and blue iso-surfaces denote filled and vacant defect states, respectively).

Lattice-like O ionosorption defects are the most important species discovered in this work. They are formed when an O adatom binds to surface Sn with chemical bonds of 2.04–2.29 Å. The resulting geometries resemble the local coordination of O in the bulk (see Figure 5), which is the reason for calling them “lattice-like” herein. Depending on the number of formed Sn–O bonds, two different lattice-like configurations can be distinguished on each termination: “T” (top) defect is recognized when a single Sn–O chemical bond occurs with the surface, whereas “B” (bridge) site is characterized by two separate chemical Sn–O bonds. It should be noted that the formed Sn–O bonds are slightly longer compared to the corresponding bonds in the bulk (2.05 Å). A common feature of the lattice-like defects is that O adatoms attract 0.39*e*–0.68*e* (see Table 3), which is considerably less compared to Bader charge of 1.12*e* computed for O atoms in bulk SnO_2_. Moreover, even this charge transfer is primarily induced by hybridization of the atomic orbitals, as evident from the atomic-like magnetic moments of 2 μ_B_ maintained by the adatoms. This behavior points to a rather weak chemical bond with the surfaces in all lattice-like configurations, which is confirmed by the formation energy calculations below. Two 2p-like orbitals of the O adatom emerge above the CBM or within the band gap, and they always remain vacant for configurations of this type (see Figure 6a, for example), affirming that O is not ionized upon interaction with neutral surfaces.

**Table 3:** Computed Properties of O Adatoms on the SnO_2_ Surfaces in Lattice-like Configurations

SnO_2_ facet	adsorption position	number of extra electrons	Bader charge on O adatom (*e*)	magnetic moment (μ_B_)	first Sn–O bond length (Å)	second Sn–O bond length (Å)
(110)	T	0	0.40	2	2.07	
		1	0.66	1	1.98	
	B	0	0.68	2	2.19	2.19
		1	0.75	1	2.17	2.17
(100)	T	0	0.39	2	2.07	
		1	0.88	1	2.01	2.05
		2	1.16	0	1.97	1.92
	B	0	0.50	2	2.29	2.29
		1	0.82	1	2.13	2.13
(101)	T	0	0.46	2	2.04	
		1	0.61	1	1.99	
	B	0	0.49	2	2.14	2.44
		1	0.78	1	2.15	2.19

**Figure 7:**
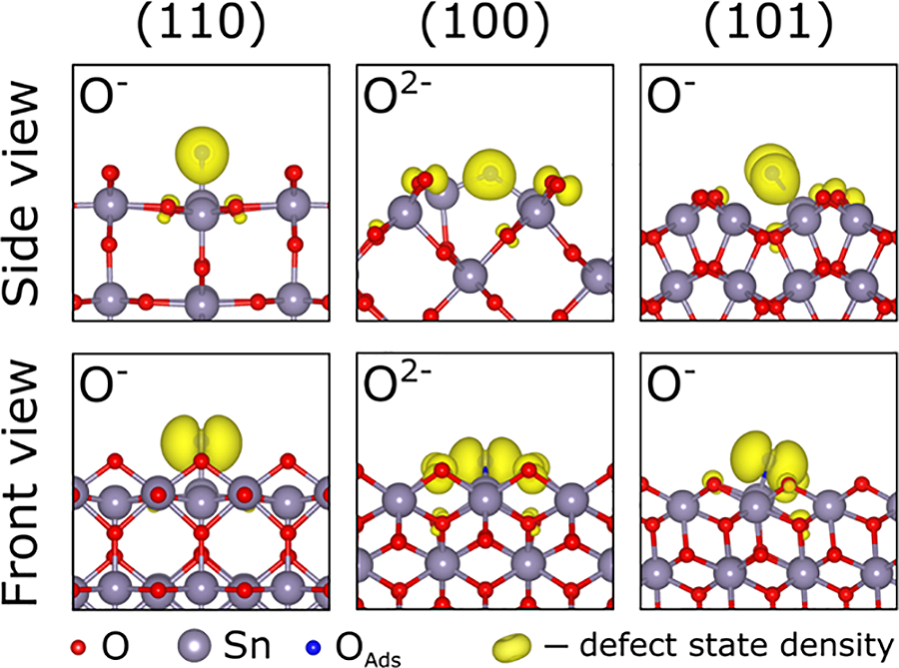
Density distribution for the highest occupied state for SnO_2_ surfaces with the T-type lattice-like O adsorption configurations. The densities are shown for the highest complete ionization states: O^–^ for the (110) and (101) terminations, O^2–^ for the (100) facet. The structures are provided in the Supporting Information.

An addition of one extra electron into the system with a lattice-like species results in several implicit signs of stabilizing O^–^ defect. First, the Sn–O bonds (where O is adatom) contracts significantly (see Table 3), suggesting stronger bonding. Second, Bader charges on O increase by 0.07*e*–0.49*e*, reflecting a higher degree of charge accumulation on the adsorbing species. Third, the 2p-like orbitals of the adatom shift down in energy, and one of them captures the introduced electron. The occupied states are located within the band gap for all except the T-type configuration on SnO_2_(100), in which the occupied defect state shifts even further down and enters the valence band (see Figure 6c). Fourth, magnetic moments change from the atomic 2 to 1 μ_B_, with the corresponding spin density (see Figure 6d) and shapes of the highest occupied orbital (see Figure 7) pointing to filling of the 2p-like orbital of the O adatom. Collectively, all of these changes evince the formation of O^–^*.*

An introduction of the second electron into the slab systems containing surface O^–^ defects changes their characteristics as expected for stabilization of O^2–^. Specifically, it leads to (i) contraction of the Sn–O bonds (where O is adatom) by 0.02–0.12 Å; (ii) increase in Bader charges on O adatoms to about 1.2*e*; and (iii) suppression of the magnetic moment toward zero for all adsorption sites in this category. However, the only defect attaining all characteristics of a true O^2–^ state is the T-type on the SnO_2_(100) surface, and this system has two in-gap defect states (one spin-up and one spin-down) filled with one electron each (see Figure 6e). These states are mostly localized on the O adatom, as shown in Figure 7. This exceptional charge localization could be due to the large displacement of surface Sn from its initial position upon the ionization of the defect.

When the second extra electron is added to the systems with any other species, the second defect state remains above the CBM of the slabs. Only a fraction of the added electron fills the defect state and the rest is instead transferred to the conduction band. An attempt was made to raise the state occupancy by increasing Hubbard *U* value to 6.0 eV. This modification was unable to fully resolve the issue though. It is worth stressing that the partial defect state occupancy in the dilute limit would cause the second extra electron to escape the defect state completely and fill the bottom of the conduction band instead. In other words, these O^2–^ defects would become O^–^ in the dilute limit, with the second extra electrons filling the CBM. As such, all these O^2–^ defects are discarded henceforth. At the same time, we recognize that further analysis would be beneficial to ascertain this conclusion. Possible verification strategies include: (i) lifting the CBM further with higher Hubbard *U* values or other (e.g., hybrid) functionals; (ii) excluding *k*-points that place the defect states above the CBM (e.g., using special off-Γ *k*-points^38,39^); (iii) fixing the occupation of otherwise partially filled states by employing *k*-dependent Fermi energy calculations;^40^ and (iv) applying an external field to flatten out the electrostatic profile within the slab and prevent possibly artificial charge transfer from the defect.^41^

### Defect Stabilities

**Figure 8:**
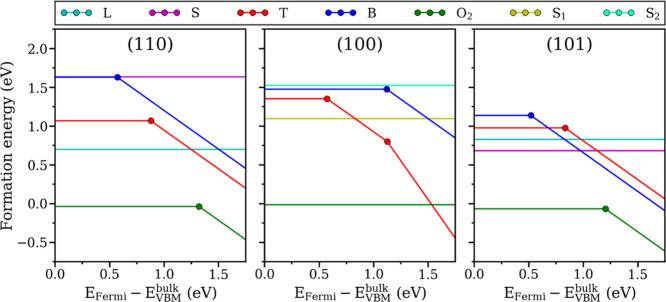
Formation energies of the oxygen ionosorption defects on SnO_2_ surfaces versus Fermi level of the bulk. The calculations are made for O-rich conditions of atmospheric exposure (see the Methods section).

The defect formation energies of each adsorption species were first analyzed individually using the uniform scaling method described in the Methods section, and then combined and presented as functions of Fermi level in Figure 8. All calculations are performed for O-rich conditions of atmospheric exposure (see the Methods section).

In the p-type region (i.e., when the Fermi level is below the mid-gap), all surface species exist in their neutral states. The most stable of them is the physisorbed O_2_ with the formation energies of about −0.07 eV on all investigated surfaces. The lowest formation energies among the O configurations are 0.68 and 0.70 eV computed for the S-type peroxide-like position on SnO_2_(101) and the L-type peroxide-like site on SnO_2_(110), implying that O adatoms are absent on all naturally occurring facets in the p-type regime. The relatively low energy of the L-type configuration on SnO_2_(110) compared to the other O species is the main reason why the majority of previous works analyzed them as a model O adsorption defect on rutile TiO_2_ and cassiterite SnO_2_.^12,15,17,18,42^ Formation energies of lattice-like defects are even higher. The most stable representatives among them are the T-type conformations on the (101) and (110) facets, with the computed formations energies of 0.98 and 1.07 eV, respectively. As such, the physisorbed O_2_ molecules are the only possible O-related adsorbates on the p-type material at atmospheric exposure, and even those are expected to persist at low temperatures only. However, the p-type regime is not of practical importance as SnO_2_ usually has high n-type conductivity, whereas making it p-type is challenging due to spontaneous compensation of acceptors with hole polarons.^36^

Remarkably different chemistry was predicted for n-type SnO_2_. This regime is more realistic because as-grown pristine SnO_2_ exhibits high n-type conductivity, although the main defect causing it remains under debate.^37,43−45^ Typical carrier concentrations for nominally undoped SnO_2_ films are in the range of 10^17^–10^19^ cm^–3^, while the Fermi level is about 0.1–0.3 eV below the CBM.^46^ The Fermi energy can further be shifted into the conduction band by n-type doping employed in the production of transparent conductive oxides (TCO).^47^ With this in mind, the discussion below is centered around the Fermi energy range within 0.3 eV from the CBM. Here, O_2_ prefers to form O_2_^–^ ions on both (110) and (101) facets, while keeping the neutral state of O_2_ physisorption on the (100) facet. The computed formation energies for the O_2_^–^ at Fermi energy fixed to CBM level of bulk SnO_2_ are −0.46 eV for the (110) and −0.62 eV for the (101) facets; low enough to expect an abundance of these species at ambient conditions. Similar stabilization of the O^–^ defects was revealed for all lattice-like configurations, but their formation energies remain positive. The only exception here is B-type defect on SnO_2_(101) having formation energy of −0.09 eV in O^–^ state, which still makes it unstable against O_2_^–^.

Most importantly, the (100) facet of n-type SnO_2_ is unique in having O^2–^ as the most stable form of chemisorbed oxygen. This defect has the T-type configuration, which undergoes a critical distortion upon ionization of O to O^–^ (or O^2–^) via the displacement of surface Sn adjacent to the adatom (see Figure 5). The computed formation energy at Fermi energy fixed to CBM for this defect is −0.45 eV, which is comparable to the formation energies of O_2_^–^ on the other facets. The exact energy values of the stable defects, however, should be treated with causion as the model approximations (i.e., slight mispositioning of the CBM due to the band gap underestimation, crude correction for O_2_ overbinding, negligence of the temperature dependence of oxygen chemical potential) may disproportionately stabilize O_2_^–^ against O^2–^. Moreover, the defect formation energies for any quantitative analysis should be extracted for the actual synthesis-dependent Fermi level. Hence, a clear conclusion at this point can only be made about the presence and nature of the most stable species for each facet.

### Uniqueness of Stable O^2–^ Species

Since O^2–^ on the (100) surface is unique in both stability of the doubly ionized state and the large distortion upon ionization, these features are likely related. To explore this hypothesis, we plotted the energy profile for relaxation of the neutral T-type configuration on (100) surface upon the addition of two extra electrons into the system. This relaxation eventually leads to the formation of stable O^2–^ discussed above. The analysis involved a two-run relaxation process (see Figure S6). The first run started from the neutral T-type configuration with the Sn atom fixed to prevent displacement (see the caption of Figure S6 for more details). During the second run, the geometry obtained from the first run was relaxed without constraints on any atom position. The results show that the energy decrease associated with the Sn displacement (extracted as the total energy difference at the ends of the first and second runs) is about 1.75 eV. Akin to all other O^2–^ species, the configuration at the end of the first run had partial occupancy of the O 2p-like defect states. At the end of the second run, these states are shifted energetically below the CBM, which allows them to capture electrons, acquire full occupancies, and thereby, greatly lower the total energy of the system. As such, a conclusion can be made that the stabilization of O^2–^ on SnO_2_(100) is indeed enabled by the Sn displacement. Conversely, the absence of stable O^2–^ on the two other surfaces is owing to the inability to displace the adjacent Sn, most likely due to the surface bonding geometry constraints.

Interestingly, coordination of the displaced Sn ion in the stable configuration of O^2–^ differs from those of the lattice cations. Instead, it resembles coordination of an interstitial Sn in a tetrahedral site in bulk SnO_2_,^37^ which itself was noted to be similar to that in SnO. This behavior could be a sign of the Sn^2+^ oxidation state, which is often related to enhanced sensing properties in experimental studies.^32,48,49^ The oxidation state was tentatively probed here with Bader charge analysis, which revealed identical charges for all surface Sn ions, thereby pointing to the Sn^4+^ oxidation state of the Sn ion in question. Still, the Sn^2+^ state cannot be ruled out as charge density analyses do not always capture variations in oxidation states.^50^ Besides, this configuration may appear as Sn^2+^ in experimental techniques sensitive to local bonding. Further investigation focusing on this defect is needed to examine this hypothesis.

Another observation supporting the dominant role of cationic displacement was made when trying to localize two electrons on the T-type configuration on SnO_2_(110) by excluding Γ point from the *k*-point grid. The logic here is that the conduction band of SnO_2_ is highly dispersive with the CBM being at the Γ point, whereas the defect states have flat bands with the same eigenvalues (±0.02 eV) at all *k*-points. As a result, the Γ point is usually responsible for the CBM locating below the highest defect state. Indeed, exclusion of the Γ point led to a proper electron localization, but the concurrent relaxation caused a large displacement of the adjacent Sn here, too. The Sn ion also attained somewhat unique coordination by shifting in the out-of-plane direction to the extent of breaking one bond with the O atom underneath, as shown in Figure S7. As expected, this structure relaxed back when Γ point was reintroduced. Despite the instability, this geometry emphasizes the importance of cationic displacement for the formation of O^2–^ species, in general.

### Experimental Insight

By combining the obtained results with previous findings, it becomes possible to pinpoint the roles played by each adsorption species. As it was concluded from the TPD analysis,^11^ the low-temperature (about 150 °C) oxygen desorption recorded for SnO_2_ after room-temperature oxidation reflects the presence of surface superoxide ions at ambient. These species are likely to be O_2_^–^ on the (110) and/or (101) surfaces. More importantly, since density functional theory (DFT) predicts that O^2–^ on the (100) termination is the only stable form of atomic oxygen, this defect stands out as the sole candidate for the high-temperature (about 500 °C) oxygen desorption observed in TPD.^11^ Hence, O^2–^ species on (100) must be responsible for the exceptional chemiresistive properties of SnO_2_.

Indeed, the nonmagnetic nature of O^2–^ explains why it is not visible to EPR,^4,10^ whereas the absence of the O^–^ signal is justified by the high formation energies of such defects. Furthermore, in light of these findings, the experimental claim that O_2_^–^ ions do not transform into other ionosorbed species (i.e., acting as a dead-end form of low-temperature oxygen adsorption)^8^ can be easily rationalized by the formation of O^2–^ and O_2_^–^ on different SnO_2_ facets. Although O^2–^ and O_2_^–^ have comparable formation energies, the higher desorption temperature of the former is reasonable because energy per atom is indeed lower for monoatomic O^2–^. The energy difference would only increase with temperature due to the rapidly rising entropy contribution to free energy for gaseous O_2_. However, an explicit inclusion of entropies is required to ascertain this claim. The usual heating of gas sensors for optimum performance can therefore be viewed as the way to remove O_2_^–^, thus activating O^2–^. Finally, the O^2–^ nature of the most stable species is consistent with the experimental dependence of the resistance *R* on the oxygen partial pressure *P*_O_2__ in dry air (i.e., *R* ∼ 1/4 *P*_O_2__),^51^ whereas the drift toward the steeper dependence upon cooling^52^ is justified by a greater portion of less stable O_2_^–^. The origin of switching to *R* ∼ 1/2 *P*_O_2__ dependence in moist air,^51^ however, needs further exploration by modeling of the interplay between the identified O-related species and H_2_O molecules on SnO_2_ surfaces.

### Refining Oxygen Ionosorption Model

**Figure 9:**
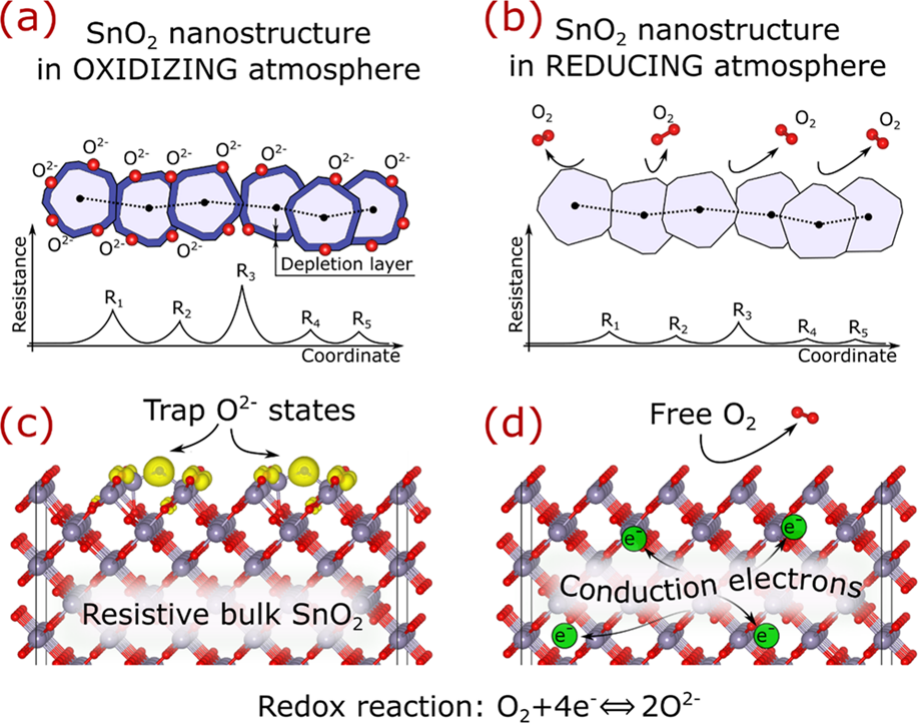
Revised oxygen ionosorption model for nanostructured SnO_2_. Illustration of polycrystalline SnO_2_ in (a) oxidizing and (b) reducing atmospheres. (c) Formation and (d) removal of the O^2–^ ions on SnO_2_(100).

With the acquired knowledge of the surface defect chemistry, the oxygen ionosorption model can now be refined for SnO_2_ nanostructures, as depicted in Figure 9*.* In an oxidizing atmosphere, the formation of O^2–^ species on SnO_2_(100) induces the appearance of the electron-depletion layer that increases the overall resistance of the sensing oxide. A likely scenario for this process is that O_2_ physisorption on SnO_2_(100) is followed by ionization to metastable O_2_^–^, promptly followed by decomposition and ionization into stable O^2–^. Owing to the similar geometry of these species, such a conversion may have a relatively low reaction barrier.

In a reducing atmosphere, the O^2–^ species escape SnO_2_ surfaces as products of chemical reactions with analyte gas or as molecular O_2_, demolishing the electron-depletion layer, and thus, decreasing the overall resistance of the sensor. During the process, stable O_2_^–^ defects on the (110) and (101) facets are not expected to form due to the operating temperature being above their desorption temperatures, while all other oxygen-related defects are absent due to their instability. Hence, all naturally occurring surfaces except SnO_2_(100) do not alter defect equilibrium during sensing, which is, therefore, the facet defining sensing properties of SnO_2_.

### Experimental Predictions

The developed understanding outlines several ways to enhance the performance of SnO_2_-based chemiresistors. First, as the (100) facet dominates the performance, rational synthesis of nanostructures with a high ratio of this facet might improve the response. Recent works have already discovered experimental routes to grow SnO_2_ nanoparticles with specific terminations.^49,53^ These nanostructures revealed facet-dependent gas-sensing characteristics, but those studies did not specifically target to produce (100) surfaces. Second, since the O^2–^ ions are found to be exclusively stable on the surfaces of n-type SnO_2_, rational tuning of carrier concentrations by means of doping may further promote gas sensing. Such tuning implies striking a perfect balance to produce sufficient conduction electron concentration and thereby promote the formation of O^2–^ species from one side, while still allowing to deplete the charge carriers under full coverage of the (100) surface with O^2–^ from the other. This effect might be responsible for the enhanced sensitivity of SnO_2_-based sensors after ion-beam irradiation,^48^ ion implantation,^54^ and n-type doping.^8^

Apart from sensing, surface ionosorption defects are important for related fields. For instance, considering a high concentration of n-type dopants in SnO_2_-based TCO materials (e.g., fluorine-doped tin oxide), the formation of O^2–^ species upon atmospheric exposure may deplete their conductivity. In this regard, the awareness of the compensating p-type oxygen ionosorption species seems indispensable for controlling the long-term degradation and synthesis-dependent properties of the TCO thin films.^47^

## Conclusions

With the extensive first-principles analysis, we refined the ionosorption model of chemiresistive gas sensing using SnO_2_ as a prototypical material. We investigated the adsorption of both atomic and molecular oxygen on naturally occurring (110), (100), and (101) surfaces of SnO_2_. In line with the oxygen ionosorption model, we focused on the charged O-related surface species. All neutral O adatom configurations were proven to be highly unstable with respect to molecular oxygen, whereas O_2_ molecules were shown to undergo weak physisorption on all surfaces. No stable ionosorption oxygen species were identified in the p-type regime. In the n-type material, O_2_^–^ ions were proven to stabilize on the (110) and (101) surfaces by trapping conduction electrons, without a possibility of further conversion to peroxide O_2_^2–^. Several configurations were shown to attain O^–^ character by trapping conduction electrons, none of which is stable with respect to O_2_ molecule and/or surface O_2_^–^. Most importantly, the formation of stable O^2–^ was revealed on the (100) surface, which is uniquely stabilized via displacement of the adjacent surface Sn. By fitting the computed properties to previously reported experimental data, the O_2_^–^ ions were concluded to desorb from the (110) and/or (101) facets at temperatures far below those of standard gas sensing, while O^2–^ ions on SnO_2_(100) were identified as the species mediating the chemiresistive effect. Implications of these results are far-reaching and include possibilities for (i) refinement of sensing models in accordance with experimental findings; (ii) outlining and controlling surface contamination of TCO films; and (iii) enhancement of gas-sensing response through preferential growth of SnO_2_(100) facets, n-type doping, etc. As such, these results are expected to provide better control over the chemistry of oxide surfaces and stimulate further advances on various fronts of material science.

## Methods

### General Computational Parameters

The analysis of oxygen ionosorption was carried out for three naturally occurring surfaces of SnO_2_, namely, (110), (100), and (101).^32^ The model slabs were constructed using the optimized unit cell of cassiterite SnO_2_, with the default systems consisting of six stoichiometric SnO_2_ trilayers, 12 Å of vacuum, and 3 × 3 supercells in lateral directions. Following common terminology, trilayers were defined as stoichiometric slices of the rutile or cassiterite crystal perpendicular to the slab surface and with cations in the middle.^19,42,48^ The first-principles calculations were performed using the Vienna *Ab initio* Simulation Package (VASP)^55^ utilizing projector augmented wave (PAW) pseudopotentials.^56^ The cutoff energy of 450 eV for the plane-wave basis set and pseudopotentials with Sn 4d^10^5s^2^5p^2^ and O 2s^2^2p^4^ valence electrons were deliberately selected for this study. Since the Perdew–Burke–Ernzerhof (PBE) exchange–correlation functional^57^ severely underestimates band gaps,^58^ which can be critical for localizing electrons on defect states, Hubbard *U* correction of various magnitudes was applied on 4d orbitals of Sn according to the formalism developed by Dudarev et al.^59^ In most cases, unless otherwise specified, the PBE+*U* scheme with the correction of *U* = 4.7 eV was used (see explanation below). Brillouin-zone integrations were performed using 5 × 2 × 1, 5 × 3 × 1, and 3 × 3 × 1 Γ-centered Monkhorst–Pack *k*-point grids^60^ for the default systems of (110), (100), and (101) facets, respectively. The ionic relaxations were performed until all Hellmann–Feynman forces in the systems decreased below 0.01 eV/Å. The presented densities of states (DOS) for defective systems were obtained using the 7 × 3 × 1, 7 × 5 × 1, and 4 × 5 × 1 *k*-point grids for the (110), (100), and (101) terminations, respectively. Symmetrical adsorption on both sides of the slab was adopted for DOS calculations. The projection operators were evaluated in real space for all supercell calculations. In contrast, unit cells of the clean surfaces were modeled using conventional reciprocal-space projections, cutoff energy of 400 eV, and Γ-centered *k*-point grids of 10 × 5 × 1, 10 × 7 × 1, and 6 × 7 × 1 for (110), (100), and (101) slabs, respectively. The supercell dimensions and *k*-point grids used for the formation energy calculations are given in Table S1. The obtained results were analyzed using the Visualisation for Electronic Structural Analysis (VESTA) software^61^ and the Python Materials Genomics (pymatgen) library.^62^

### Bader Charge Analysis

The nature of ionosorbed species was explored by comparing charges of different ions using the Bader partitioning scheme.^63^ The obtained charges were further adjusted to a convenient zero reference level by subtracting computed charges of isolated atoms. Hence, the presented charges should be treated as charge transfers induced by all bonds of the ion of interest.

### Band Edge Positions

Energies of band edges with respect to the vacuum level were calculated for the SnO_2_ surfaces by adjusting in-plane-average potential in the middle of the vacuum slab to zero, as discussed in detail elsewhere.^64^

### Screening for Equilibrium Conformations

Oxygen ionosorption defects were identified by optimizing a total of 500 systems with different O and O_2_ adsorption sites in the presence of zero, one, and two extra electrons. The initial adsorption positions were generated under constraint for a minimum distance of 1.6 (2.4) Å between the atoms of the adsorbing species and the nearest surface O (Sn) atom. The initial tests revealed that localization of extra charge and related defect properties are sensitive to the conduction band minimum (CBM) position (more details below). Hence, to allow stabilization of the different ionized states, the screening was done independently with PBE and PBE+*U* (*U* = 3.5 eV) methods. Considering the large number of the initial configurations, the screening calculations were carried out using smaller 2 × 2 surface supercells and 0.04 eV/Å atomic force threshold for the ionic relaxation. All identified configurations in the most relevant charged states are provided in the Supporting Information and shared via the MaterialsCloud repository^65^ under the following identifier.^66^

### Choice of PBE+*U* Parameters

Not only the band gaps^58^ but also basic defect properties depend strongly on the exchange–correlation functional.^67^ Indeed, changes in the ionization states and related properties were observed for several defects discussed herein when altering the Hubbard *U* correction value. This is not surprising considering that the PBE functional yields the band gap of 0.67 eV for bulk SnO_2_, which is more than 5 times smaller than the experimental value of 3.6 eV.^32^ This means that acceptor states lying between the experimental and computed CBM cannot localize electrons on the PBE level of theory, but must be able to do so in reality. As a result, the true ionization states of the defect species are inaccessible. In the case of SnO_2_, the lowest conduction band is highly dispersive,^36,47^ and hence, partial occupancy of such defect states can still occur when the portion of the conduction band below is filled. However, this yields inaccurate properties of the ionosorption species. To resolve this issue, we applied Hubbard *U* correction on Sn 4d electrons intending to tune CBM of SnO_2_ to the target level set by hybrid Heyd–Scuseria–Ernzerhof (HSE06) calculations with the default mixing parameter of 0.25^68^ (see Figure S1). This adjustment was possible because *U* correction primely shifts the conduction band of SnO_2_ upward in energy by introducing additional lattice compression while leaving the valence band maximum (VBM) position almost unaffected (see Figure S2). As one can see, the best matching between the CBM levels from PBE+*U* and HSE06 is found at *U* of 4.7 eV, and therefore, this value was adopted as a default. Noteworthy, since the remaining band gap underestimation is due to inaccurate positioning of the VBM, all properties of acceptor species in n-type regime must be reasonably estimated. Despite the improvement in terms of the band gap, HSE06 still underestimates its magnitude by yielding 2.86 eV for the bulk. Nonetheless, we decided to use the default HSE06 settings as further adjustments seem unjustified. All main conclusions were replicated with Hubbard *U* = 3.5 eV correction (this value was optimized for bulk SnO_2_ earlier,^43^ and it is also found to better reproduce the experimental cell volume than *U* = 4.7 eV; see Figure S2), providing nearly identical results.

### Avoiding Dielectric Breakthrough

Another important reason for using Hubbard *U* correction of 4.7 eV is that the larger energy gap helps to avoid defect-induced dielectric breakthrough. This effect occurs when the oxygen atom placed in proximity of the SnO_2_ surface with extra electrons traps this extra charge, inducing electrostatic potential throughout the slab in such a way that the occupied defect state shifts above the CBM level on the other (defect-free) slab surface. This situation leads to an artificial charge transfer between the opposite slab surfaces. A similar phenomenon can occur under a constant electric field when the height of the sawtooth potential exceeds band gap energy.^41,69^ The artificial breakthrough introduces spurious phenomena, which can distort defect properties significantly. Based on the experience gained from dealing with this problem, we suspect that such a breakthrough exacerbated the convergence issues that led to fractional magnetic moments in the works of Golovanov et al.^23,24^ We plan to discuss more details and related examples of such a phenomenon in a future publication.

### Formation Energy Calculations

Formation energy is the main thermodynamic parameter for estimating defect concentration in bulk solids and on their surfaces (where it is usually expressed via adsorption energy). Positive formation energy implies that the adsorbing species is unstable with respect to molecules in the surrounding medium (i.e., formation through adsorption is unfavorable). The conventional approach to calculating the formation energy of defect *X* in charged state *q* can be represented as
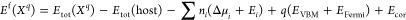
1where *E*_tot_(*X*^*q*^) and *E*_tot_(host) are total energies of the defective and pristine systems, respectively, *n*_*i*_ is the total number of atoms added to (*n*_*i*_ > 0) or removed from (*n*_*i*_ < 0) the system in exchange with the reservoir of the *i*th element, Δμ_*i*_ + *E*_*i*_ is the energy of the *i*th element in the reservoir consisting of the total energy in the most stable state of the *i*th element (*E*_*i*_) and a chemical potential related to this state (Δμ_*i*_), *E*_Fermi_ is the Fermi energy with respect to VBM of the host system (*E*_VBM_), and *E*_cor_ is the collective energy correction term for all errors arising from the periodic cell approximation.^40,70−72^ In this work, *E*_*i*_ was calculated as half of the total energy of an isolated O_2_ molecule, while Δμ_*i*_ was set to zero describing oxygen-rich conditions of atmospheric exposure. An artificial overbinding of O_2_ molecule^73,74^ was corrected by adding an empirical value of 0.5 eV to the formation energies for all defects without chemical O–O bonding (i.e., lattice-like ionosorption defects; see the Results and Discussion section). Similar energy shifts for chemical potentials are commonly implemented when calculating the formation energies of oxides^73−75^ and defects therein.^70,76^ However, this simple approximation does not account for a different degree of overbinding in O_2_^–^ species, which may lead to a partisan stabilization of atomic O over molecular O_2_ ionosorption. Finding a configuration-dependent correction may require a detailed comparative analysis of different species with various functionals, which is beyond the scope of this study. Meanwhile, all conclusions herein are formulated to be independent of the exact value of implemented correction for O_2_ overbinding.

The *E*_VBM_ term in eq 1 was additionally corrected for the existence of surface states (Δ*E*_VBM_^SS^) and band bending (Δ*E*_VBM_^BB^) effect on the SnO_2_ surfaces (see the Results and Discussion section) as
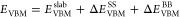
2where *E*_VBM_^slab^ is VBM of the clean slab with the thickness of *h* trilayers; Δ*E*_VBM_^BB^ is a difference in vacuum-relative VBM levels for infinitely thick SnO_2_ slab (*h* → ∞; approximated by the 24-trilayer-thick systems in this study; see Figure S3) and that with a thickness of *h* (0 < *h* ≤ ∞) atomic trilayers
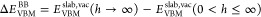
3and Δ*E*_VBM_^SS^ is a difference between as-computed VBM position for bulk SnO_2_ (*E*_VBM_^bulk^) and the 24-trilayer-thick slabs (*E*_VBM_^slab^ (*h* → ∞)) plus an alignment term Δ*V* calculated as a difference in the average electrostatic potential at cores of Sn atoms in two middle trilayers of the slab and that in bulk SnO_2_
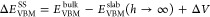
4

### Correction for Spurious Electrostatic Interaction

**Figure 10:**
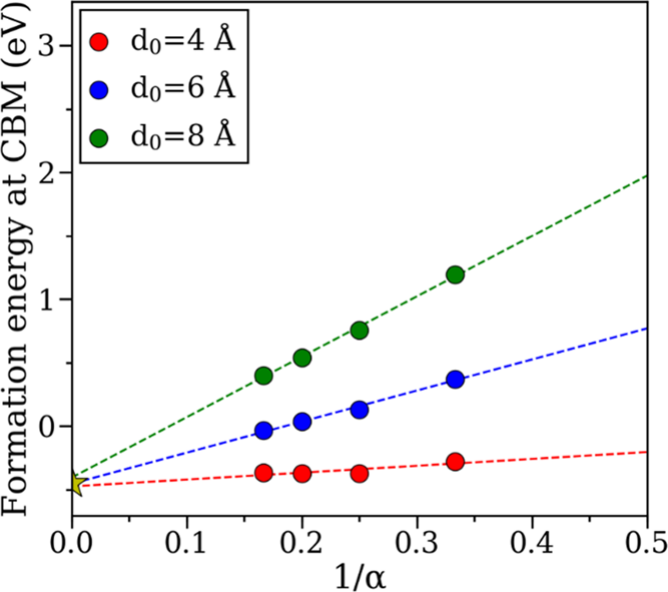
Calculation of the formation energy (at *E*_Fermi_ = *E*_CBM_) for O^2–^ in the T-type configuration on SnO_2_(100) using the uniform scaling method. Lateral dimensions, material slab thickness, and vacuum slab thickness in the systems are given by α × α, 2α, and α·*d*_0_, respectively.

The *E*_cor_ term in eq 1 is made by electrostatic interaction in charged periodic slab systems. Recently, Komsa et al. proposed an elegant post-treatment procedure to correct the artificial electrostatics by scaling the system uniformly in all directions.^77^ In the original work, the authors used model charge and fixed dielectric profile for the slab, and, shortly thereafter, Vinichenko et al. proposed to replace it with the actual charge distribution for defect states.^72^ In other works, the dielectric profile obtained from the linear response was replaced with the renormalized profile of charge density.^71^ To avoid the ambiguity, herein, we proceed by (i) extracting all energies from DFT directly, (ii) calculating the uncorrected defect formation energies using eq 1 with *E*_cor_ = 0, and then (iii) extrapolating the results for an infinite cell. To ensure convergence of the results, three independent uniform scaling procedures were performed for the slabs with different vacuum thicknesses (given by α·d_0_, where α is scaling coefficient, *d*_0_ = 4, 6, and 8 Å), and the final values were taken by averaging these three fits. The results are exemplified in Figure 10 for surface O^2–^ on the SnO_2_(100) surface (in the T-type configuration; see above) and for other defects in Figure S4*.* As one can see, the formation energies converged to ±0.05 eV, indicating a good accuracy of the employed method.

### Role of van der Waals (vdW) Forces

Dispersion interactions are yet another factor capable of disproportionally stabilizing certain species. To evaluate the magnitude of this effect, we calculated formation energies of the most stable ionosorption defects at each facet using optB88-vdW functional^78^ plus Hubbard *U* of 4.7 eV. The obtained values were then compared with those from PBE+*U* calculations on the same slabs. We estimated that vdW interactions lower the formation energies of the physisorbed O_2_ and chemisorbed O_2_^–^ by about 0.2 and 0.5 eV, respectively. The stronger effect for the latter is explained by the relative proximity to the surfaces (see Figure 2). For the T-type configuration of O on SnO_2_(100), the formation energy was lowered equally by about 0.3 eV in all three ionization states, reflecting that the distance between O adatom and the surface does not change much upon ionization. Hence, it can be concluded that vdW correction leads to a firmer binding with the surfaces for all species, with a slightly stronger effect for O_2_^–^, but these differences are insufficient to alter the described chemistry of oxygen ionosorption in a significant way. As such, and recognizing the sensitivity of the material properties to the choice of vdW functional,^79^ even the most accurate of which are not able to reproduce the true charge density distribution in weakly bonded systems,^80^ we decided to exclude dispersion forces from this study.

## Supporting Information Available

Slab dimensions and the *k*-point grids used; comparison of the band edge positions computed with PBE+*U* and HSE06; effect of Hubbard *U* correction value on the computed properties of SnO_2_ bulk and surfaces; illustration of the band bending effect; uniform scaling curves for the O^–^ and O_2_^–^ defects; element- and spin-resolved DOS, as well as spin densities for O_2_ on SnO_2_(101) with zero and one extra electron; energy evolution profile for the relaxation of the T-type configuration on SnO_2_(100) upon the addition of two extra electrons; optimized geometry of the T-type configuration of O^2–^ on SnO_2_(110) when the defect states are fully occupied; structures of all identified adsorption species in the most relevant charged states, which are also accessible via the MaterialsCloud repository^65^ under the following identifier^66^ (PDF)
