# Supplementary material for: Chemistry of Oxygen Ionosorption on SnO2 Surfaces
Source: ACS Appl Mater Interfaces. 2021 Jul 12;13(28):33664–76. doi: 10.1021/acsami.1c08236 (PMC8397246; doi:10.1021/acsami.1c08236)
Supplement: Supplementary file 1 — am1c08236_si_001.pdf [file am1c08236_si_001.pdf]

Supporting information

Chemistry of Oxygen Ionosorption on SnO_2 Surfaces

Kostiantyn V. Sopiha[1,], Oleksandr I. Malyi[2], Clas Persson[3,4], and Ping Wu[5,*]*

1 – Solar Cell Technology, Department of Materials Science and Engineering, Uppsala University, Box 534, SE-75121 Uppsala, Sweden

2 – Renewable and Sustainable Energy Institute, University of Colorado, Boulder, Colorado 80309, USA

3 – Centre for Materials Science and Nanotechnology/Department of Physics, University of Oslo, P. O. Box 1048 Blindern, NO-0316 Oslo, Norway

4 – Division of Applied Materials Physics, Department of Materials Science and Engineering, KTH Royal Institute of Technology, SE-10044 Stockholm, Sweden

5 – Entropic Interface Group, Engineering Product Development, Singapore University of Technology and Design, 8 Somapah Road, 487372, Singapore

E-mails: kostiantyn.sopiha@gmail.com (K.S.), wuping@sutd.edu.sg (W.P.)

Keywords: Ionosorption model, chemiresistive sensing, SnO_2, charged oxygen species, surface chemistry.

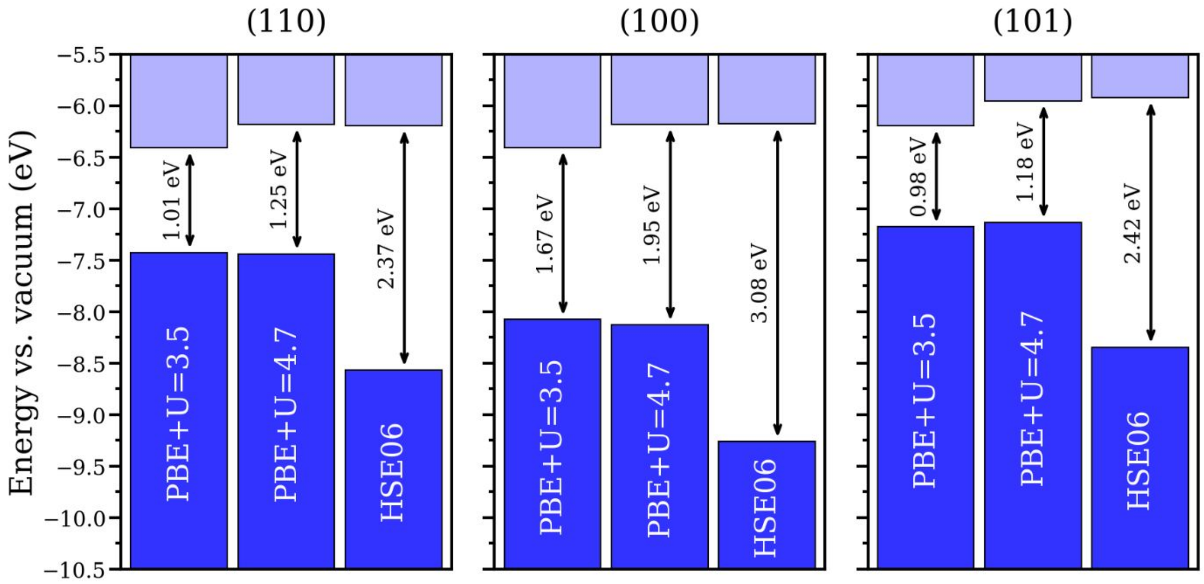

Figure S1: Comparison of the band edge positions of 7-trilayer-thick SnO_2 slabs computed using PBE+U and HSE06 functionals. In contrast to the rest of this work, the HSE06 calculations were performed using pseudopotentials with Sn $5s^25p^2$ valence electron configuration.

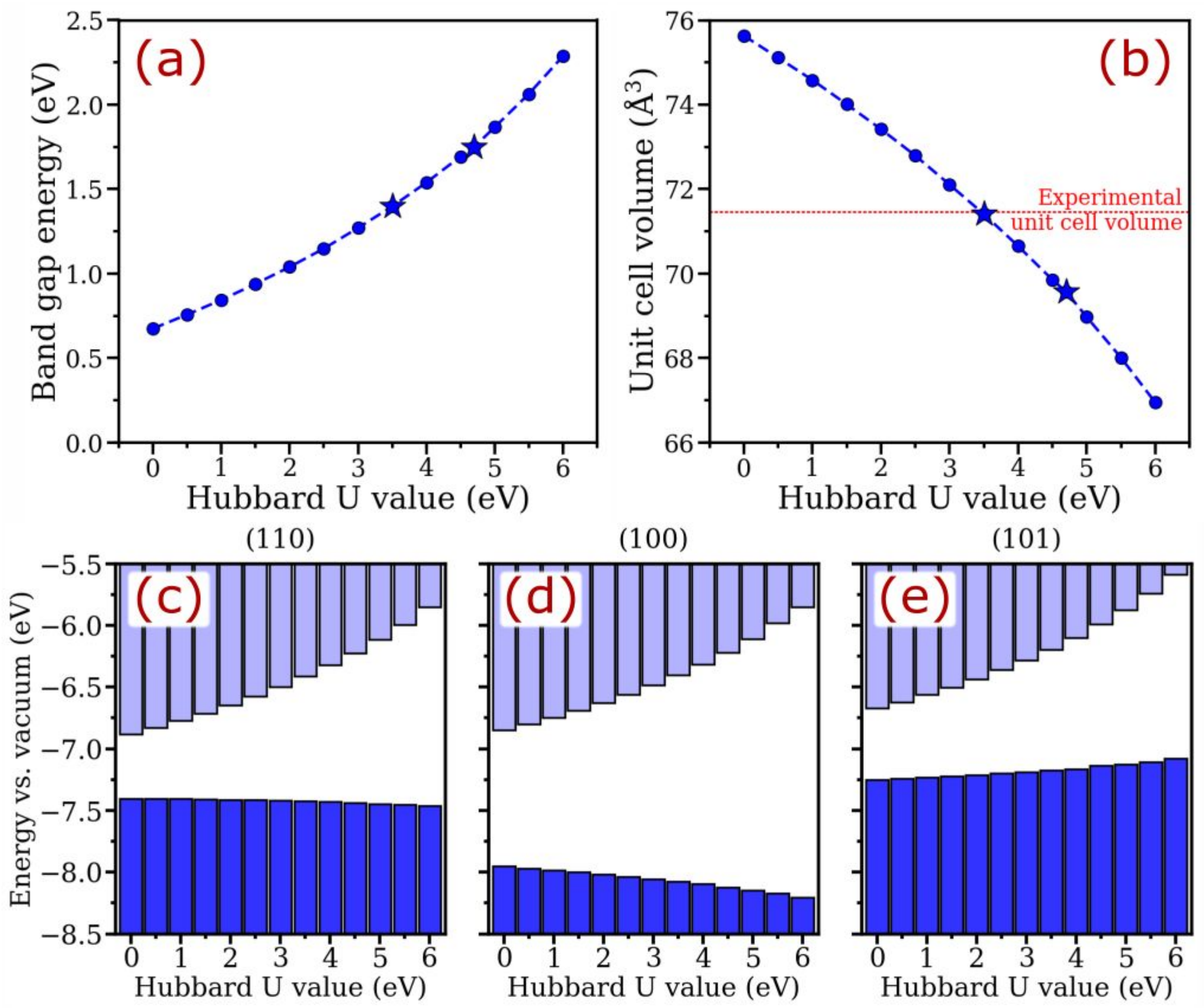

Figure S2: The effect of Hubbard U correction on (a) band gap energy and (b) unit cell volume, and band edge positions of the naturally occurring (c) (110), (d) (100), and (e) (101) surfaces of SnO_2. The star markers represent U values of primary interest.

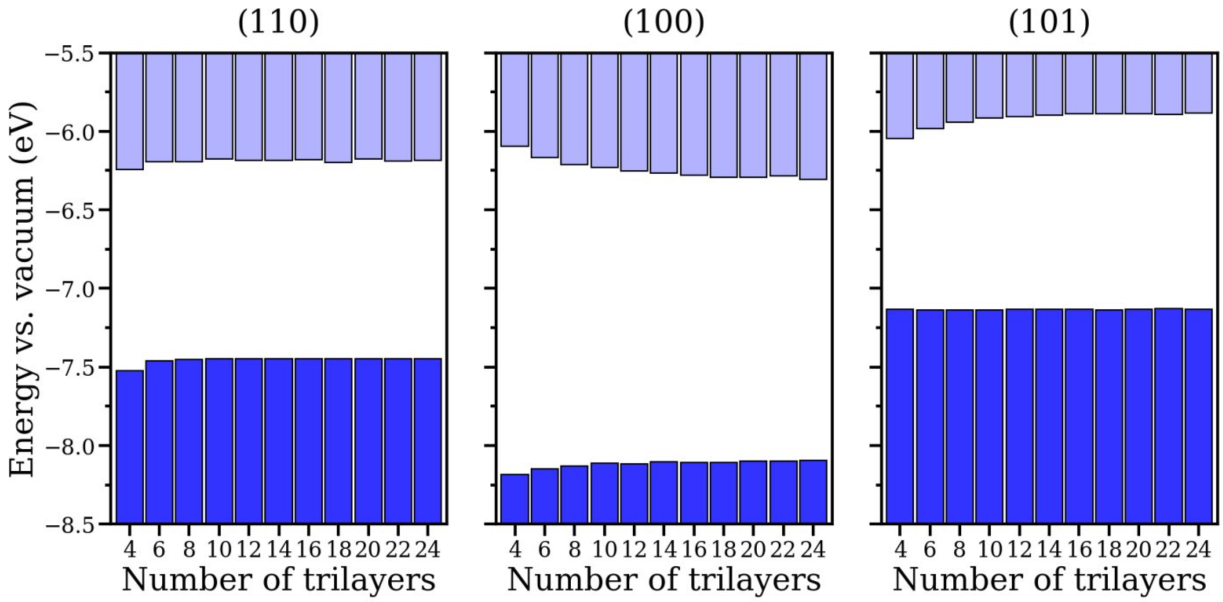

Figure S3: Illustration of the band bending effect as a change in the positions of CBM and VBM levels for the SnO_2 slabs with the thickness.

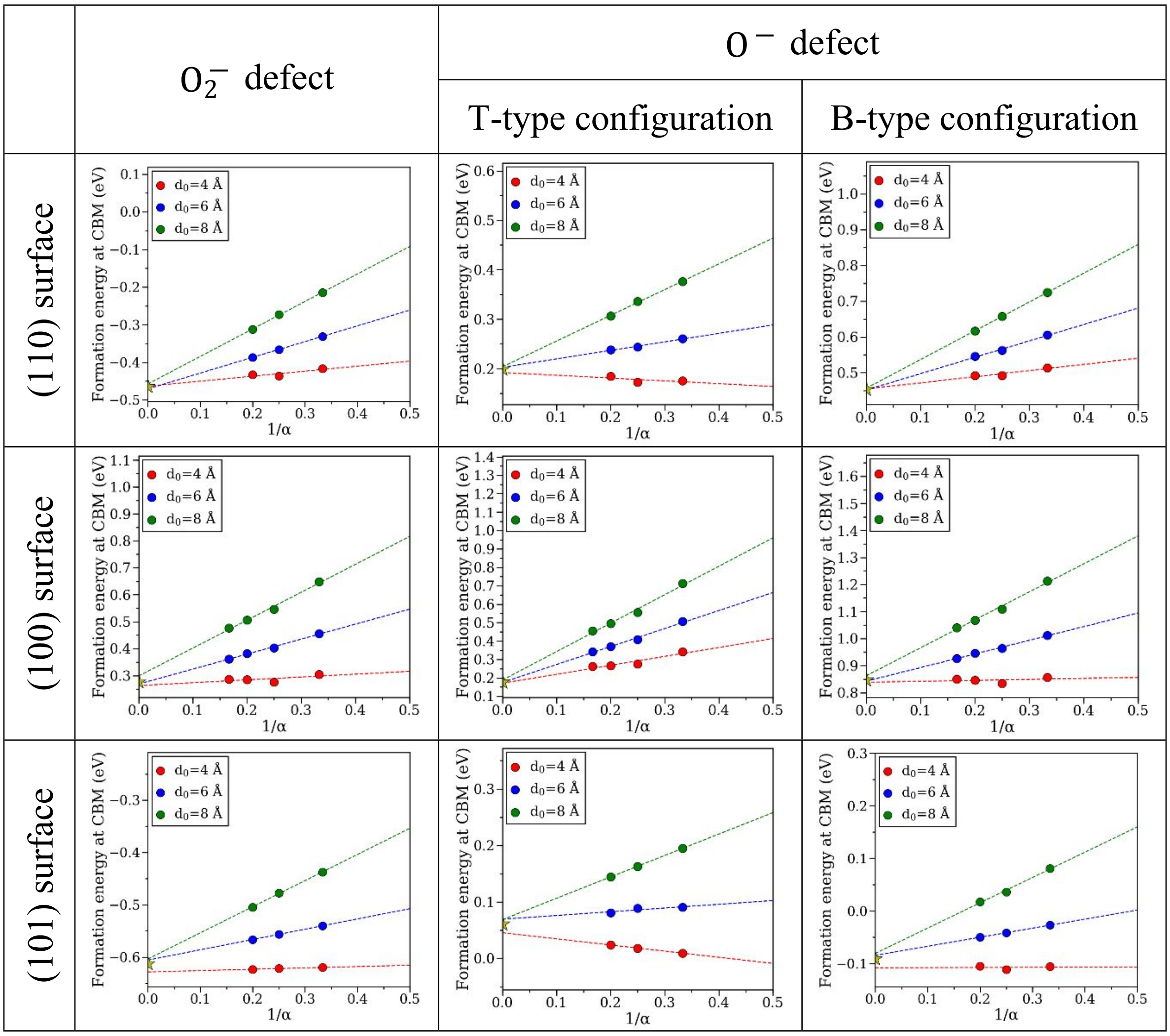

Figure S4: Uniform scaling curves for formation energies of the singly ionized ionosorption defects on SnO_2 surfaces.

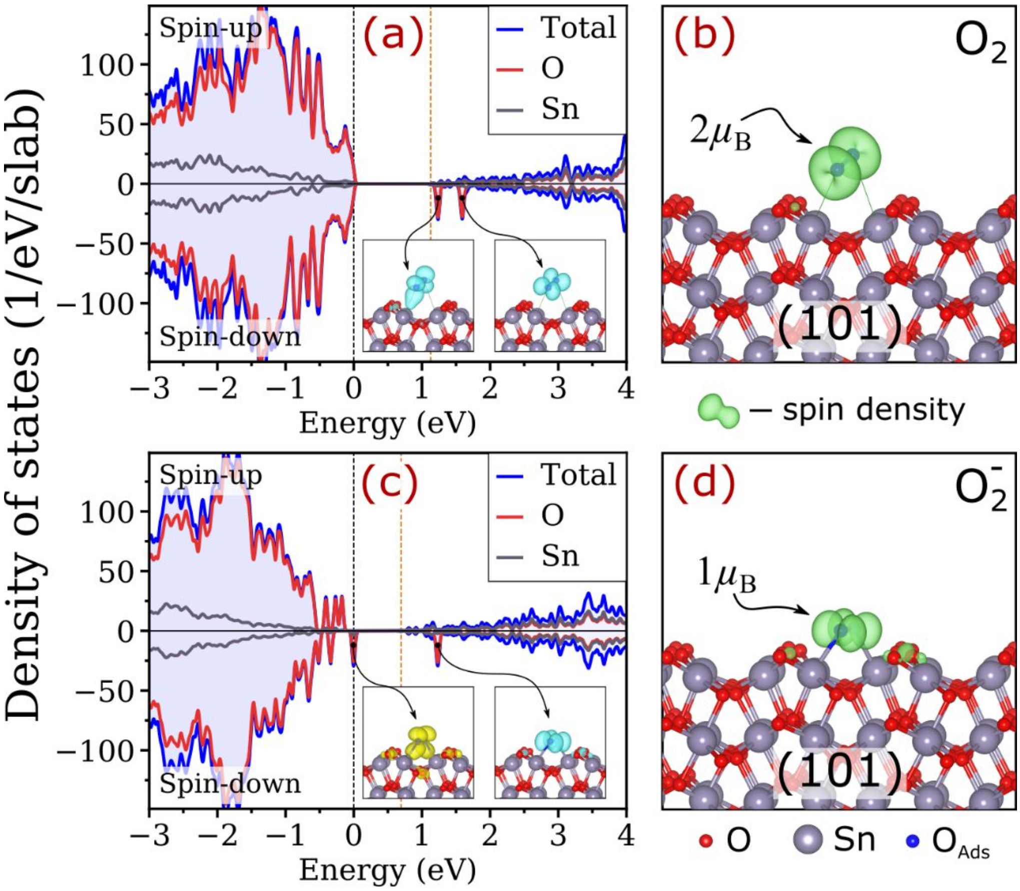

Figure S5: Analysis of the defect states for O_2 adsorbing on the $SnO_2(101)$ surface in different charged states. (a,c) element- and spin-resolved DOS and (b,d) spin densities for the species on the slab containing (a,b) zero and (c,d) one extra electron. The black and orange vertical dashed lines in (a,c) indicate the highest occupied state and principal CBM of the slab, respectively. The insets in (a,c) illustrate change densities projected on the defect states (yellow and blue iso-surfaces denote filled and vacant defect states, respectively).

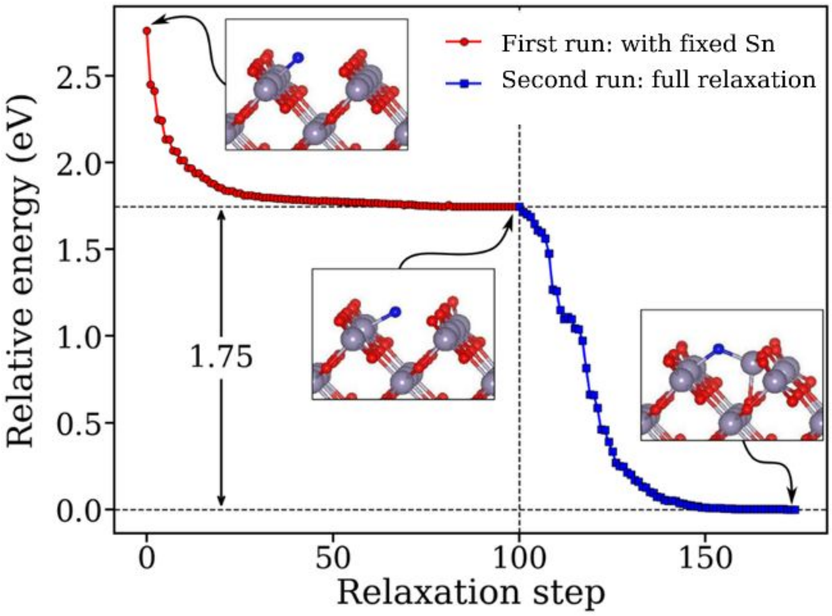

Figure S6: Energy evolution profile during the relaxation of the T-type configuration of O adatom on SnO_2(100) surface upon the addition of two extra electrons into the system. The relaxation consists of two runs. During the first run (shown by red markers), displacement of the Sn atom, which otherwise would form the second Sn-O bond in the stable O^{2-} configuration, was restrained. This was achieved by fixing one coordinate (along the displacement axis) for this Sn atom. To prevent the counter-movement of the slab together with O adatom towards the fixed Sn atom, the same coordinate was fixed for two other Sn atoms in remote regions of the slab. The second run (blue markers) started from the configuration optimized in the first run and continued without constraints of any atom position. To facilitate the formation of non-magnetic O^{2-}, both runs were performed without spin polarization.

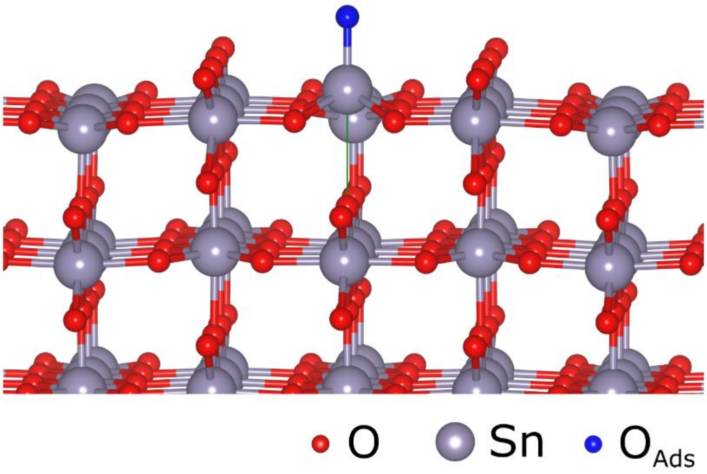

Figure S7: Optimized geometry of the T-type configuration of O^{2-} on SnO_2(110) surface obtained when the defect state is fully occupied (obtained by removing Γ point from k-point grid). The Sn ion adjacent to the adsorbing O is significantly displaced in out-of-plane direction.

SnO_2 surface	Scaling coefficient (α)	Lateral dimensions (unit cells)	Thickness (# of trilayers)	k-point grid	Number of atoms
(110)	3	3×3	6	2×1×1	324
	4	4×4	8	1×1×1	768
	5	5×5	10	1×1×1	1500
(100)	3	3×3	6	2×2×1	162
	4	4×4	8	2×1×1	384
	5	5×5	10	1×1×1	750
	6	6×6	12	1×1×1	1296
(101)	3	3×3	6	1×2×1	324
	4	4×4	8	1×1×1	768
	5	5×5	10	1×1×1	1500

Table S1: The slab dimensions and the Γ-centered k-point grids used for the formation energy calculations.

List of structures (links)

Structure 1: Configuration of $O_2^{=}$ on SnO_2(110) surface. S-11
Structure 2: Configuration of $O_2^{=}$ on SnO_2(100) surface. S-19
Structure 3: Configuration of $O_2^{=}$ on SnO_2(101) surface. S-24
Structure 4: T-type configuration of O = on SnO_2(110) surface. S-32
Structure 5: T-type configuration of O^{2} = on SnO_2(100) surface...................S-40
Structure 6: T-type configuration of O = on SnO_2(101) surface. S-45
Structure 7: B-type configuration of O = on SnO_2(110) surface. S-53
Structure 8: B-type configuration of O = on SnO_2(100) surface. S-61
Structure 9: B-type configuration of O = on SnO_2(101) surface. S-66
Structure 10: S-type configuration of O on SnO_2(110) surface. S-74
Structure 11: S_1-type configuration of O on SnO_2(100) surface.....................S-82
Structure 12: S-type configuration of O on SnO_2(101) surface. S-87
Structure 13: L-type configuration of O on SnO_2(110) surface......................S-95
Structure 14: S_2-type configuration of O on SnO_2(100) surface..................S-103
Structure 15: L-type configuration of O on SnO_2(101) surface...................S-108

Structure 1: Configuration of O_2^- on $SnO_2(110)$ surface.

```
_cell_length_a   9.42435700
_cell_length_b   19.96585700
_cell_length_c   37.96585700
_cell_angle_alpha   90.00000000
_cell_angle_beta   90.00000000
_cell_angle_gamma   90.00000000
loop_
 _atom_site_type_symbol
 _atom_site_fract_x
 _atom_site_fract_y
 _atom_site_fract_z
  Sn  0.833178  0.665191  0.723193
  Sn  0.833201  0.334378  0.723198
  Sn  0.670628  0.499806  0.718849
  Sn  0.995651  0.499813  0.718873
  Sn  0.166595  0.333921  0.724581
  Sn  0.166591  0.665671  0.724580
  Sn  0.166556  0.999792  0.724524
  Sn  0.499812  0.333916  0.724600
  Sn  0.499808  0.665666  0.724593
  Sn  0.500008  0.999798  0.724549
  Sn  0.833306  0.999796  0.724556
  Sn  0.166727  0.166399  0.629059
  Sn  0.165555  0.499787  0.629866
  Sn  0.166723  0.833137  0.629047
  Sn  0.499863  0.166421  0.629053
  Sn  0.500873  0.499777  0.629862
  Sn  0.499859  0.833137  0.629054
  Sn  0.833294  0.166446  0.629012
  Sn  0.833236  0.499788  0.632118
  Sn  0.833273  0.833087  0.629007
  Sn  0.166686  0.333170  0.370489
  Sn  0.166684  0.666513  0.370486
  Sn  0.166684  0.999839  0.370327
  Sn  0.500034  0.333156  0.370501
  Sn  0.500034  0.666520  0.370484
  Sn  0.500031  0.999849  0.370329
  Sn  0.833363  0.333170  0.370494
  Sn  0.833368  0.666517  0.370488
  Sn  0.833377  0.999837  0.370327
  Sn  0.166689  0.166609  0.274741
```

Sn	0.166692	0.499884	0.275121
Sn	0.166684	0.833172	0.274753
Sn	0.500026	0.166605	0.274746
Sn	0.500032	0.499886	0.275142
Sn	0.500025	0.833166	0.274755
Sn	0.833372	0.166607	0.274739
Sn	0.833358	0.499886	0.275121
Sn	0.833369	0.833172	0.274752
Sn	0.999838	0.167045	0.715577
Sn	0.999832	0.832532	0.715576
Sn	0.333249	0.166787	0.715755
Sn	0.333167	0.499793	0.715653
Sn	0.333246	0.832798	0.715752
Sn	0.666623	0.167028	0.715575
Sn	0.666631	0.832567	0.715575
Sn	0.000240	0.332857	0.633492
Sn	0.000209	0.666698	0.633487
Sn	0.999953	0.999764	0.633546
Sn	0.333276	0.332934	0.633575
Sn	0.333247	0.666636	0.633571
Sn	0.333314	0.999776	0.633554
Sn	0.666280	0.332877	0.633493
Sn	0.666235	0.666676	0.633493
Sn	0.666641	0.999775	0.633555
Sn	0.000028	0.166534	0.365882
Sn	0.000003	0.499849	0.366320
Sn	0.000027	0.833143	0.365886
Sn	0.333345	0.166529	0.365888
Sn	0.333367	0.499847	0.366327
Sn	0.333348	0.833152	0.365896
Sn	0.666710	0.166532	0.365889
Sn	0.666704	0.499847	0.366329
Sn	0.666702	0.833152	0.365896
Sn	0.000025	0.333250	0.283898
Sn	0.000031	0.666526	0.283893
Sn	0.000034	0.999889	0.283739
Sn	0.333368	0.333220	0.283903
Sn	0.333368	0.666542	0.283897
Sn	0.333380	0.999898	0.283749
Sn	0.666685	0.333219	0.283905
Sn	0.666694	0.666546	0.283897
Sn	0.666684	0.999896	0.283748
Sn	0.166670	0.333155	0.545058

Sn	0.166683	0.666380	0.545048
Sn	0.166652	0.999768	0.544906
Sn	0.499948	0.333155	0.545048
Sn	0.499944	0.666381	0.545047
Sn	0.499989	0.999776	0.544910
Sn	0.833319	0.333143	0.545030
Sn	0.833302	0.666392	0.545019
Sn	0.833329	0.999765	0.544915
Sn	0.166682	0.166482	0.454448
Sn	0.166547	0.499807	0.455003
Sn	0.166678	0.833113	0.454447
Sn	0.500009	0.166494	0.454434
Sn	0.500152	0.499806	0.455021
Sn	0.500007	0.833114	0.454433
Sn	0.833353	0.166481	0.454444
Sn	0.833354	0.499804	0.455112
Sn	0.833349	0.833114	0.454443
Sn	0.000009	0.166440	0.542141
Sn	0.999588	0.499772	0.543187
Sn	0.000000	0.833075	0.542132
Sn	0.333339	0.166415	0.542149
Sn	0.333311	0.499760	0.542869
Sn	0.333347	0.833145	0.542145
Sn	0.666629	0.166437	0.542139
Sn	0.667045	0.499765	0.543199
Sn	0.666604	0.833123	0.542137
Sn	0.000016	0.333112	0.457427
Sn	0.000020	0.666489	0.457423
Sn	0.000016	0.999795	0.457280
Sn	0.333338	0.333126	0.457418
Sn	0.333349	0.666477	0.457412
Sn	0.333350	0.999798	0.457271
Sn	0.666681	0.333117	0.457432
Sn	0.666680	0.666488	0.457427
Sn	0.666683	0.999797	0.457272
O	0.498585	0.561693	0.722897
O	0.833144	0.564878	0.713235
O	0.498612	0.437893	0.722913
O	0.833157	0.434708	0.713282
O	0.668270	0.665032	0.754305
O	0.668291	0.334433	0.754301
O	0.998149	0.665036	0.754284
O	0.998178	0.334471	0.754290

O	0.661391	0.499781	0.664789
O	0.166449	0.104548	0.722608
O	0.167728	0.437908	0.722924
O	0.166616	0.771608	0.723532
O	0.500090	0.104543	0.722672
O	0.499870	0.771603	0.723485
O	0.833245	0.104688	0.722703
O	0.833167	0.771299	0.723130
O	0.167292	0.267121	0.630655
O	0.166949	0.601756	0.631082
O	0.166682	0.934441	0.631338
O	0.499356	0.267139	0.630685
O	0.499525	0.601734	0.631108
O	0.499939	0.934450	0.631353
O	0.833206	0.267197	0.630724
O	0.833193	0.601821	0.631565
O	0.833268	0.934424	0.631303
O	0.166692	0.101047	0.367443
O	0.166693	0.434370	0.367790
O	0.166703	0.767727	0.367504
O	0.500024	0.101054	0.367436
O	0.500033	0.434351	0.367844
O	0.500020	0.767749	0.367565
O	0.833363	0.101045	0.367445
O	0.833338	0.434360	0.367761
O	0.833355	0.767732	0.367507
O	0.166683	0.271553	0.276220
O	0.166739	0.604830	0.276391
O	0.166765	0.938188	0.276168
O	0.500027	0.271530	0.276235
O	0.500034	0.604827	0.276427
O	0.500027	0.938182	0.276170
O	0.833373	0.271550	0.276216
O	0.833318	0.604834	0.276391
O	0.833298	0.938185	0.276170
O	0.166611	0.227978	0.723506
O	0.167775	0.561698	0.722898
O	0.166420	0.895039	0.722594
O	0.499842	0.227970	0.723503
O	0.500080	0.895044	0.722656
O	0.833186	0.228263	0.723118
O	0.833272	0.894893	0.722698
O	0.166669	0.065079	0.631348

O	0.166951	0.397816	0.631099
O	0.167277	0.732434	0.630657
O	0.499956	0.065112	0.631371
O	0.499577	0.397818	0.631089
O	0.499333	0.732425	0.630680
O	0.833291	0.065102	0.631295
O	0.833228	0.397745	0.631563
O	0.833130	0.732345	0.630725
O	0.166716	0.231940	0.367523
O	0.166688	0.565324	0.367793
O	0.166687	0.898627	0.367467
O	0.500023	0.231931	0.367591
O	0.500038	0.565327	0.367798
O	0.500022	0.898632	0.367487
O	0.833348	0.231943	0.367532
O	0.833350	0.565335	0.367779
O	0.833362	0.898627	0.367469
O	0.166747	0.061599	0.276163
O	0.166737	0.394945	0.276412
O	0.166696	0.728225	0.276237
O	0.500030	0.061612	0.276192
O	0.500024	0.394933	0.276424
O	0.500026	0.728218	0.276219
O	0.833319	0.061599	0.276165
O	0.833305	0.394945	0.276412
O	0.833374	0.728231	0.276242
O	0.999949	0.999780	0.754985
O	0.333186	0.331534	0.754997
O	0.333184	0.668059	0.754993
O	0.333233	0.999769	0.754968
O	0.666678	0.999770	0.754992
O	0.999995	0.166167	0.663652
O	0.004965	0.499817	0.664793
O	0.999987	0.833347	0.663651
O	0.333282	0.166330	0.663775
O	0.333171	0.499743	0.663729
O	0.333261	0.833241	0.663775
O	0.666565	0.166116	0.663644
O	0.666580	0.833416	0.663647
O	0.000027	0.333024	0.403472
O	0.000023	0.666655	0.403472
O	0.000029	0.999842	0.403349
O	0.333351	0.332997	0.403476

O	0.333366	0.666621	0.403466
O	0.333333	0.999850	0.403331
O	0.666703	0.332995	0.403481
O	0.666689	0.666635	0.403472
O	0.666723	0.999846	0.403331
O	0.000017	0.166649	0.312737
O	0.000044	0.499831	0.313109
O	0.000022	0.833160	0.312744
O	0.333290	0.166667	0.312746
O	0.333357	0.499889	0.313126
O	0.333304	0.833108	0.312758
O	0.666767	0.166666	0.312746
O	0.666693	0.499886	0.313124
O	0.666762	0.833106	0.312759
O	0.002540	0.328594	0.685934
O	0.002569	0.670978	0.685930
O	0.999862	0.999778	0.686403
O	0.333082	0.331999	0.686378
O	0.333064	0.667611	0.686373
O	0.333279	0.999814	0.686411
O	0.663794	0.328616	0.685944
O	0.663771	0.670956	0.685941
O	0.666763	0.999809	0.686414
O	0.000026	0.166435	0.420232
O	0.999904	0.499804	0.420677
O	0.000020	0.833154	0.420229
O	0.333292	0.166454	0.420234
O	0.333370	0.499843	0.420685
O	0.333261	0.833151	0.420236
O	0.666739	0.166461	0.420235
O	0.666796	0.499822	0.420690
O	0.666759	0.833153	0.420236
O	0.000023	0.333140	0.335587
O	0.000035	0.666554	0.335583
O	0.000035	0.999834	0.335439
O	0.333354	0.333151	0.335592
O	0.333330	0.666557	0.335587
O	0.333381	0.999871	0.335440
O	0.666711	0.333130	0.335593
O	0.666725	0.666569	0.335587
O	0.666684	0.999864	0.335439
O	0.000033	0.166576	0.244251
O	0.000023	0.499906	0.244614

O	0.000030	0.833192	0.244260
O	0.333365	0.166598	0.244253
O	0.333382	0.499860	0.244639
O	0.333358	0.833153	0.244268
O	0.666686	0.166596	0.244253
O	0.666673	0.499864	0.244636
O	0.666686	0.833151	0.244268
O	0.166668	0.101688	0.542657
O	0.166455	0.435077	0.543139
O	0.166798	0.768568	0.542733
O	0.500004	0.101677	0.542666
O	0.500168	0.435058	0.543127
O	0.499952	0.768579	0.542756
O	0.833300	0.101700	0.542680
O	0.833309	0.434931	0.542869
O	0.833199	0.768564	0.542737
O	0.166689	0.268409	0.455834
O	0.166651	0.601904	0.455972
O	0.166709	0.935156	0.455785
O	0.499999	0.268417	0.455864
O	0.500025	0.601910	0.456012
O	0.500013	0.935167	0.455757
O	0.833346	0.268403	0.455840
O	0.833366	0.601919	0.456001
O	0.833324	0.935155	0.455785
O	0.166757	0.230978	0.542746
O	0.166463	0.564458	0.543113
O	0.166642	0.897845	0.542660
O	0.499957	0.230981	0.542776
O	0.500156	0.564448	0.543140
O	0.499996	0.897874	0.542679
O	0.833275	0.230979	0.542755
O	0.833316	0.564602	0.542847
O	0.833313	0.897831	0.542688
O	0.166698	0.064430	0.455813
O	0.166662	0.397704	0.455988
O	0.166713	0.731197	0.455851
O	0.500021	0.064434	0.455757
O	0.500018	0.397699	0.456017
O	0.500001	0.731203	0.455853
O	0.833332	0.064431	0.455813
O	0.833355	0.397683	0.456006
O	0.833337	0.731199	0.455853

O	0.999896	0.332690	0.578679
O	0.999898	0.666833	0.578667
O	0.000011	0.999789	0.578727
O	0.333279	0.332734	0.578623
O	0.333298	0.666830	0.578621
O	0.333285	0.999755	0.578726
O	0.666718	0.332771	0.578664
O	0.666693	0.666800	0.578661
O	0.666662	0.999752	0.578729
O	0.000005	0.166360	0.488573
O	0.000130	0.499800	0.489169
O	0.000001	0.833214	0.488564
O	0.333380	0.166377	0.488574
O	0.333315	0.499797	0.489099
O	0.333384	0.833237	0.488571
O	0.666644	0.166372	0.488571
O	0.666580	0.499787	0.489187
O	0.666636	0.833234	0.488568
O	0.000061	0.165751	0.595648
O	0.997917	0.499803	0.596600
O	0.000027	0.833759	0.595637
O	0.333291	0.165489	0.595665
O	0.333249	0.499767	0.596219
O	0.333316	0.834024	0.595660
O	0.666559	0.165759	0.595650
O	0.668661	0.499790	0.596607
O	0.666526	0.833824	0.595649
O	0.000065	0.332830	0.510440
O	0.000067	0.666758	0.510434
O	0.999981	0.999766	0.510367
O	0.333283	0.333051	0.510424
O	0.333318	0.666487	0.510422
O	0.333307	0.999753	0.510365
O	0.666623	0.332841	0.510443
O	0.666592	0.666682	0.510440
O	0.666703	0.999753	0.510368
O	0.903610	0.499906	0.773556
O	0.761750	0.499883	0.773503

Structure 2: Configuration of O_2^- on $SnO_2(100)$ surface.

```
_cell_length_a   9.42441600
_cell_length_b   14.11538000
_cell_length_c   32.11538000
_cell_angle_alpha   90.00000000
_cell_angle_beta   90.00000000
_cell_angle_gamma   90.00000000
loop_
 _atom_site_type_symbol
 _atom_site_fract_x
 _atom_site_fract_y
 _atom_site_fract_z
  Sn  0.333589  0.662124  0.684176
  Sn  0.333196  0.332696  0.536574
  Sn  0.000065  0.326235  0.684099
  Sn  0.338069  0.326038  0.687256
  Sn  0.666561  0.662121  0.684176
  Sn  0.666916  0.332690  0.536575
  Sn  0.662065  0.326041  0.687255
  Sn  0.000069  0.661743  0.684051
  Sn  0.167108  0.166715  0.609532
  Sn  0.166891  0.497091  0.611140
  Sn  0.500076  0.170281  0.611417
  Sn  0.500068  0.495638  0.610673
  Sn  0.833046  0.166708  0.609531
  Sn  0.833238  0.497093  0.611139
  Sn  0.166705  0.160847  0.315734
  Sn  0.166717  0.495326  0.315562
  Sn  0.166714  0.828267  0.315687
  Sn  0.500030  0.160835  0.315713
  Sn  0.500054  0.495357  0.315556
  Sn  0.500033  0.828280  0.315681
  Sn  0.833358  0.160846  0.315735
  Sn  0.833389  0.495321  0.315562
  Sn  0.833354  0.828265  0.315687
  Sn  0.166703  0.165943  0.463167
  Sn  0.166739  0.499157  0.463150
  Sn  0.166858  0.831871  0.463746
  Sn  0.500048  0.165910  0.463158
  Sn  0.500058  0.499003  0.463218
  Sn  0.500055  0.832182  0.463858
  Sn  0.833394  0.165945  0.463166
```

Sn	0.833368	0.499159	0.463151
Sn	0.833253	0.831875	0.463747
Sn	0.166834	0.832694	0.609835
Sn	0.500062	0.832822	0.609686
Sn	0.833293	0.832692	0.609834
Sn	0.000052	0.332587	0.389682
Sn	0.000043	0.666113	0.389959
Sn	0.000041	0.998681	0.390115
Sn	0.333404	0.332603	0.389665
Sn	0.333450	0.666089	0.389987
Sn	0.333371	0.998735	0.390112
Sn	0.666696	0.332601	0.389666
Sn	0.666642	0.666090	0.389989
Sn	0.666707	0.998735	0.390112
Sn	0.000054	0.333169	0.536326
Sn	0.000059	0.664542	0.537225
Sn	0.000063	0.999471	0.536641
Sn	0.333431	0.664453	0.537198
Sn	0.333651	0.000277	0.536930
Sn	0.666691	0.664461	0.537198
Sn	0.666470	0.000278	0.536928
Sn	0.000075	0.995521	0.683772
Sn	0.333635	0.996222	0.683607
Sn	0.666519	0.996222	0.683603
O	0.331960	0.434916	0.580815
O	0.665594	0.101484	0.581219
O	0.668164	0.434902	0.580820
O	0.334585	0.565021	0.638450
O	0.328384	0.235216	0.636202
O	0.665551	0.565031	0.638446
O	0.671780	0.235211	0.636202
O	0.000067	0.566908	0.637680
O	0.000076	0.233155	0.636472
O	0.166334	0.071325	0.656482
O	0.166841	0.738650	0.656838
O	0.166161	0.404709	0.658243
O	0.500069	0.068780	0.655276
O	0.500066	0.737694	0.655813
O	0.500068	0.395460	0.653610
O	0.833817	0.071324	0.656481
O	0.833281	0.738651	0.656839
O	0.834007	0.404713	0.658243
O	0.166499	0.623268	0.716899

O	0.164511	0.282357	0.716181
O	0.500073	0.599186	0.565167
O	0.500079	0.630447	0.718184
O	0.500056	0.267696	0.564068
O	0.500074	0.249436	0.706342
O	0.833367	0.599581	0.565280
O	0.833649	0.623256	0.716897
O	0.834200	0.266770	0.563393
O	0.835615	0.282370	0.716183
O	0.166712	0.065470	0.361799
O	0.166760	0.399366	0.361380
O	0.166780	0.732889	0.361770
O	0.500035	0.065500	0.361792
O	0.500057	0.399396	0.361378
O	0.500040	0.732877	0.361749
O	0.833364	0.065471	0.361801
O	0.833351	0.399362	0.361380
O	0.833305	0.732886	0.361767
O	0.166678	0.063831	0.507626
O	0.166718	0.397373	0.507366
O	0.166704	0.729501	0.508171
O	0.500065	0.063542	0.507398
O	0.500057	0.397187	0.507517
O	0.500054	0.729589	0.508207
O	0.833443	0.063843	0.507627
O	0.833383	0.397371	0.507368
O	0.833419	0.729502	0.508171
O	0.166803	0.266810	0.417348
O	0.166742	0.600239	0.417618
O	0.166763	0.932910	0.417880
O	0.500043	0.266826	0.417363
O	0.500050	0.600094	0.417600
O	0.500040	0.933034	0.417885
O	0.833295	0.266805	0.417346
O	0.833351	0.600241	0.417619
O	0.833322	0.932913	0.417882
O	0.165922	0.266775	0.563390
O	0.166749	0.599577	0.565278
O	0.166832	0.933881	0.564524
O	0.500060	0.933444	0.564346
O	0.833294	0.933886	0.564521
O	0.167102	0.954901	0.716262
O	0.500086	0.955409	0.716269

O	0.833051	0.954894	0.716257
O	0.000028	0.120329	0.283031
O	0.000053	0.455320	0.282760
O	0.000037	0.788173	0.282884
O	0.333334	0.120292	0.283009
O	0.333379	0.455387	0.282737
O	0.333359	0.788208	0.282869
O	0.666721	0.120288	0.283010
O	0.666733	0.455378	0.282740
O	0.666706	0.788207	0.282867
O	0.000045	0.100811	0.434409
O	0.000047	0.434619	0.434159
O	0.000048	0.767404	0.434758
O	0.333299	0.101023	0.434360
O	0.333414	0.434552	0.434184
O	0.333551	0.767452	0.434774
O	0.666791	0.101023	0.434359
O	0.666690	0.434543	0.434186
O	0.666555	0.767456	0.434776
O	0.000077	0.100612	0.581115
O	0.000057	0.433726	0.581618
O	0.000062	0.767446	0.581461
O	0.334535	0.101477	0.581222
O	0.333080	0.767504	0.581276
O	0.667039	0.767511	0.581275
O	0.000041	0.238780	0.342078
O	0.000043	0.572737	0.342209
O	0.000030	0.905403	0.342349
O	0.333330	0.238786	0.342052
O	0.333337	0.572737	0.342212
O	0.333390	0.905450	0.342343
O	0.666743	0.238787	0.342051
O	0.666769	0.572722	0.342218
O	0.666682	0.905452	0.342342
O	0.000048	0.231720	0.490565
O	0.000063	0.564258	0.491201
O	0.000063	0.897406	0.491451
O	0.333466	0.231503	0.490791
O	0.333385	0.564367	0.491116
O	0.333177	0.897747	0.491512
O	0.666637	0.231504	0.490790
O	0.666733	0.564373	0.491119
O	0.666933	0.897748	0.491512

O	0.000062	0.899622	0.637492
O	0.333165	0.899552	0.637496
O	0.666968	0.899552	0.637495
O	0.570902	0.427011	0.735060
O	0.429193	0.427005	0.735057

Structure 3: Configuration of O_2^- on SnO_2(101) surface.

```
_cell_length_a   16.97242900
_cell_length_b   14.11702300
_cell_length_c   33.67701300
_cell_angle_alpha   90.00000000
_cell_angle_beta   90.00000000
_cell_angle_gamma   90.00000000
loop_
 _atom_site_type_symbol
 _atom_site_fract_x
 _atom_site_fract_y
 _atom_site_fract_z
  Sn  0.381151  0.175215  0.688461
  Sn  0.390984  0.509120  0.693776
  Sn  0.489390  0.498659  0.613203
  Sn  0.551897  0.658345  0.688634
  Sn  0.547391  0.324769  0.693707
  Sn  0.157373  0.164091  0.612765
  Sn  0.157745  0.497400  0.613000
  Sn  0.158192  0.829286  0.613019
  Sn  0.491568  0.165496  0.613648
  Sn  0.490761  0.831765  0.612184
  Sn  0.823876  0.164431  0.613184
  Sn  0.823734  0.497230  0.612919
  Sn  0.824345  0.830888  0.612748
  Sn  0.029661  0.165722  0.457497
  Sn  0.029538  0.499098  0.457388
  Sn  0.029757  0.832382  0.457371
  Sn  0.362874  0.166066  0.457307
  Sn  0.362898  0.499191  0.457401
  Sn  0.363070  0.832549  0.457409
  Sn  0.696290  0.166115  0.457542
  Sn  0.696113  0.499055  0.457548
  Sn  0.696410  0.832587  0.457299
  Sn  0.238988  0.153302  0.302840
  Sn  0.238980  0.486683  0.302795
  Sn  0.239082  0.819976  0.302804
  Sn  0.572338  0.153367  0.302831
  Sn  0.572353  0.486642  0.302859
  Sn  0.572447  0.819985  0.302855
  Sn  0.905674  0.153370  0.302912
  Sn  0.905640  0.486563  0.302908
```

Sn	0.905738	0.819987	0.302819
Sn	0.050396	0.180453	0.689151
Sn	0.051175	0.513379	0.689243
Sn	0.050655	0.846043	0.689035
Sn	0.384018	0.846564	0.688846
Sn	0.715566	0.181642	0.689614
Sn	0.716429	0.514004	0.689203
Sn	0.717339	0.847865	0.688939
Sn	0.259874	0.168018	0.534888
Sn	0.260088	0.500647	0.535078
Sn	0.260091	0.833485	0.535051
Sn	0.593158	0.168420	0.535189
Sn	0.592952	0.500452	0.535238
Sn	0.593399	0.834531	0.534680
Sn	0.926469	0.167607	0.535076
Sn	0.926329	0.500712	0.534930
Sn	0.926667	0.834258	0.534846
Sn	0.132131	0.169112	0.379446
Sn	0.132090	0.502557	0.379373
Sn	0.132211	0.835785	0.379381
Sn	0.465467	0.169287	0.379368
Sn	0.465459	0.502527	0.379412
Sn	0.465572	0.835854	0.379420
Sn	0.798807	0.169246	0.379495
Sn	0.798746	0.502427	0.379508
Sn	0.798881	0.835860	0.379364
Sn	0.216604	0.985545	0.688894
Sn	0.217868	0.319045	0.689215
Sn	0.219551	0.650920	0.689809
Sn	0.549706	0.986144	0.688647
Sn	0.883791	0.986983	0.689119
Sn	0.883618	0.320434	0.689222
Sn	0.883845	0.652912	0.689067
Sn	0.093552	0.998768	0.534946
Sn	0.093159	0.332512	0.534916
Sn	0.093306	0.665442	0.534870
Sn	0.426646	0.000050	0.534791
Sn	0.426192	0.332422	0.535139
Sn	0.426504	0.665685	0.534970
Sn	0.759887	0.999459	0.534901
Sn	0.759169	0.332481	0.535545
Sn	0.759957	0.665780	0.534782
Sn	0.298729	0.330997	0.379357

Sn	0.298877	0.664255	0.379380
Sn	0.298840	0.997489	0.379374
Sn	0.632082	0.330910	0.379481
Sn	0.632187	0.664179	0.379442
Sn	0.632167	0.997676	0.379407
Sn	0.965385	0.330830	0.379537
Sn	0.965501	0.664208	0.379364
Sn	0.965513	0.997512	0.379411
Sn	0.324246	0.002353	0.612250
Sn	0.324418	0.336255	0.613464
Sn	0.325459	0.667235	0.613676
Sn	0.657639	0.003487	0.612837
Sn	0.655441	0.336577	0.614341
Sn	0.657480	0.669512	0.612410
Sn	0.991041	0.002420	0.612846
Sn	0.990810	0.336076	0.612887
Sn	0.990847	0.668905	0.612761
Sn	0.196497	0.000739	0.457422
Sn	0.196212	0.334342	0.457361
Sn	0.196413	0.667425	0.457372
Sn	0.529707	0.001286	0.457372
Sn	0.529459	0.334305	0.457501
Sn	0.529663	0.667434	0.457451
Sn	0.863028	0.000981	0.457402
Sn	0.862693	0.334142	0.457662
Sn	0.863025	0.667532	0.457345
Sn	0.072366	0.013431	0.302843
Sn	0.072282	0.346692	0.302901
Sn	0.072396	0.680092	0.302808
Sn	0.405722	0.013424	0.302827
Sn	0.405632	0.346826	0.302805
Sn	0.405759	0.680093	0.302825
Sn	0.739040	0.013513	0.302861
Sn	0.738965	0.346724	0.302905
Sn	0.739084	0.679992	0.302874
O	0.455866	0.255635	0.714032
O	0.457425	0.616258	0.713658
O	0.560353	0.269106	0.636704
O	0.561078	0.603485	0.633745
O	0.315667	0.395333	0.669323
O	0.650084	0.399626	0.669806
O	0.482769	0.434818	0.666620
O	0.287045	0.224618	0.714171

O	0.624117	0.231502	0.716131
O	0.293270	0.563074	0.715239
O	0.624998	0.569261	0.714572
O	0.393667	0.564075	0.636033
O	0.226353	0.268352	0.633654
O	0.226880	0.602147	0.634117
O	0.227173	0.934548	0.633669
O	0.561995	0.934465	0.633519
O	0.894134	0.268618	0.634096
O	0.894155	0.601691	0.633867
O	0.894459	0.934957	0.634009
O	0.099184	0.269757	0.480403
O	0.099367	0.602761	0.480394
O	0.099499	0.936061	0.480449
O	0.432651	0.269490	0.480561
O	0.432719	0.602803	0.480446
O	0.432829	0.936554	0.480372
O	0.765872	0.269641	0.480821
O	0.766162	0.602831	0.480414
O	0.766178	0.936344	0.480446
O	0.306718	0.269181	0.323069
O	0.306907	0.602422	0.323074
O	0.306886	0.935692	0.323085
O	0.640096	0.269128	0.323160
O	0.640267	0.602361	0.323148
O	0.640245	0.935796	0.323121
O	0.973380	0.269116	0.323195
O	0.973553	0.602401	0.323091
O	0.973535	0.935717	0.323114
O	0.122811	0.271091	0.714555
O	0.124760	0.602482	0.715068
O	0.123352	0.936145	0.714925
O	0.456903	0.935664	0.715057
O	0.790716	0.270162	0.715042
O	0.790870	0.603021	0.714942
O	0.790790	0.936405	0.715088
O	0.328877	0.270780	0.558304
O	0.329967	0.602867	0.558640
O	0.329540	0.936954	0.557898
O	0.662203	0.270943	0.559040
O	0.663039	0.603705	0.558118
O	0.662986	0.937563	0.558230
O	0.995992	0.270619	0.558327

O	0.996077	0.603591	0.558232
O	0.996273	0.937021	0.558287
O	0.201984	0.269488	0.402909
O	0.202050	0.602759	0.402919
O	0.202100	0.935961	0.402953
O	0.535362	0.269429	0.403018
O	0.535373	0.602777	0.402983
O	0.535453	0.936200	0.402959
O	0.868625	0.269442	0.403129
O	0.868700	0.602795	0.402945
O	0.868753	0.936069	0.402969
O	0.087349	0.064076	0.588853
O	0.087209	0.397685	0.588880
O	0.087345	0.730428	0.588851
O	0.420712	0.064992	0.588412
O	0.419789	0.398200	0.588583
O	0.420845	0.730592	0.588853
O	0.754195	0.064704	0.588860
O	0.752983	0.397618	0.589461
O	0.754130	0.731368	0.588613
O	0.293219	0.063124	0.433450
O	0.293001	0.396694	0.433422
O	0.293221	0.729817	0.433484
O	0.626613	0.063426	0.433508
O	0.626337	0.396596	0.433561
O	0.626551	0.729819	0.433534
O	0.959906	0.063111	0.433544
O	0.959617	0.396460	0.433644
O	0.959903	0.729775	0.433508
O	0.165670	0.063586	0.276964
O	0.165605	0.396882	0.277025
O	0.165768	0.730201	0.276964
O	0.499034	0.063638	0.276960
O	0.499010	0.396972	0.276989
O	0.499176	0.730175	0.277009
O	0.832379	0.063672	0.277022
O	0.832317	0.396845	0.277057
O	0.832479	0.730079	0.277035
O	0.314618	0.062342	0.668111
O	0.315627	0.732375	0.668745
O	0.648961	0.064740	0.668763
O	0.650110	0.733923	0.667801
O	0.982506	0.064500	0.668755

O	0.982698	0.397817	0.668804
O	0.982481	0.730805	0.668676
O	0.189834	0.064114	0.511338
O	0.189663	0.397520	0.511349
O	0.189769	0.730498	0.511336
O	0.523241	0.064903	0.511263
O	0.522821	0.397502	0.511422
O	0.523009	0.731045	0.511233
O	0.856499	0.064265	0.511397
O	0.856081	0.397307	0.511662
O	0.856557	0.730821	0.511316
O	0.062084	0.065023	0.357863
O	0.061983	0.398373	0.357907
O	0.062143	0.731657	0.357824
O	0.395380	0.065156	0.357808
O	0.395341	0.398500	0.357803
O	0.395499	0.731727	0.357819
O	0.728752	0.065188	0.357872
O	0.728696	0.398390	0.357918
O	0.728833	0.731659	0.357875
O	0.148606	0.101640	0.668643
O	0.148989	0.435498	0.668698
O	0.149296	0.768114	0.668989
O	0.480754	0.096175	0.666990
O	0.483774	0.772966	0.667546
O	0.815604	0.102348	0.668961
O	0.816579	0.436298	0.668844
O	0.816251	0.769268	0.668661
O	0.023151	0.102442	0.511480
O	0.022949	0.435822	0.511364
O	0.023171	0.769070	0.511345
O	0.356211	0.102852	0.511152
O	0.356198	0.435616	0.511185
O	0.356438	0.768799	0.511274
O	0.689811	0.103075	0.511507
O	0.689584	0.435775	0.511530
O	0.689961	0.769359	0.511207
O	0.228727	0.101617	0.357845
O	0.228631	0.435010	0.357785
O	0.228801	0.768331	0.357802
O	0.562079	0.101738	0.357840
O	0.562011	0.434930	0.357850
O	0.562191	0.768381	0.357863

O	0.895424	0.101698	0.357923
O	0.895343	0.434924	0.357926
O	0.895504	0.768400	0.357837
O	0.253547	0.102011	0.588628
O	0.253776	0.435566	0.588975
O	0.254773	0.767913	0.588983
O	0.587602	0.103403	0.589006
O	0.586548	0.436319	0.589219
O	0.587506	0.769494	0.588309
O	0.920644	0.102556	0.589071
O	0.920448	0.435715	0.588935
O	0.920865	0.769141	0.588842
O	0.126526	0.103423	0.433548
O	0.126384	0.436904	0.433473
O	0.126554	0.770068	0.433489
O	0.459785	0.103713	0.433434
O	0.459693	0.436809	0.433473
O	0.459893	0.770227	0.433507
O	0.793197	0.103699	0.433611
O	0.793041	0.436828	0.433656
O	0.793298	0.770285	0.433495
O	0.332337	0.103121	0.276997
O	0.332301	0.436517	0.276924
O	0.332471	0.769877	0.276971
O	0.665707	0.103217	0.277004
O	0.665681	0.436446	0.277014
O	0.665849	0.769866	0.277040
O	0.999003	0.103194	0.277048
O	0.998946	0.436385	0.277031
O	0.999109	0.769893	0.276975
O	0.060221	0.231159	0.633838
O	0.060622	0.564828	0.634003
O	0.061011	0.897806	0.633901
O	0.392414	0.229317	0.633489
O	0.393779	0.898847	0.633561
O	0.727300	0.232054	0.634362
O	0.728041	0.566319	0.634067
O	0.728507	0.899687	0.633915
O	0.265786	0.230520	0.480303
O	0.266066	0.563876	0.480544
O	0.266062	0.896805	0.480468
O	0.599426	0.230870	0.480598
O	0.599320	0.563703	0.480624

O	0.599622	0.897494	0.480332
O	0.932636	0.230360	0.480577
O	0.932650	0.563757	0.480466
O	0.932859	0.897165	0.480422
O	0.140042	0.230865	0.323122
O	0.140147	0.564377	0.323077
O	0.140246	0.897633	0.323084
O	0.473439	0.231023	0.323085
O	0.473552	0.564363	0.323117
O	0.473639	0.897683	0.323107
O	0.806764	0.230961	0.323192
O	0.806846	0.564262	0.323180
O	0.806947	0.897690	0.323091
O	0.290504	0.897095	0.714685
O	0.623642	0.897134	0.714629
O	0.956708	0.229991	0.714820
O	0.957672	0.563746	0.714854
O	0.957122	0.896668	0.714634
O	0.162306	0.229257	0.558114
O	0.162798	0.562711	0.558323
O	0.162879	0.895375	0.558339
O	0.496398	0.230500	0.558798
O	0.496025	0.563219	0.558345
O	0.496161	0.896673	0.557838
O	0.829384	0.229684	0.558573
O	0.829432	0.562935	0.558332
O	0.829767	0.896556	0.558262
O	0.035331	0.230516	0.403021
O	0.035329	0.563995	0.402952
O	0.035398	0.897176	0.402945
O	0.368701	0.230814	0.402900
O	0.368725	0.564047	0.402950
O	0.368732	0.897231	0.402922
O	0.702087	0.230784	0.403066
O	0.702026	0.563957	0.403056
O	0.702100	0.897331	0.402908
O	0.516371	0.407983	0.746596
O	0.443576	0.443585	0.747690

Structure 4: T-type configuration of O^- on $SnO_2(110)$ surface.

```
_cell_length_a   9.42435700
_cell_length_b   19.96585700
_cell_length_c   37.96585700
_cell_angle_alpha   90.00000000
_cell_angle_beta   90.00000000
_cell_angle_gamma   90.00000000
loop_
 _atom_site_type_symbol
 _atom_site_fract_x
 _atom_site_fract_y
 _atom_site_fract_z
  Sn  0.499225  0.334602  0.724305
  Sn  0.499235  0.665131  0.724302
  Sn  0.834159  0.334597  0.724306
  Sn  0.834164  0.665142  0.724300
  Sn  0.332919  0.499863  0.715086
  Sn  0.666798  0.499870  0.722355
  Sn  0.166683  0.333265  0.724575
  Sn  0.166698  0.666482  0.724569
  Sn  0.166645  0.999867  0.724584
  Sn  0.500032  0.999864  0.724576
  Sn  0.833253  0.999865  0.724558
  Sn  0.166652  0.166409  0.629078
  Sn  0.166731  0.499801  0.628307
  Sn  0.166650  0.833160  0.629074
  Sn  0.499965  0.166495  0.629041
  Sn  0.502223  0.499800  0.631276
  Sn  0.499962  0.833099  0.629040
  Sn  0.833337  0.166465  0.629035
  Sn  0.831262  0.499797  0.631287
  Sn  0.833337  0.833103  0.629037
  Sn  0.166652  0.333174  0.370507
  Sn  0.166647  0.666527  0.370506
  Sn  0.166629  0.999848  0.370397
  Sn  0.499986  0.333172  0.370515
  Sn  0.499980  0.666528  0.370509
  Sn  0.499985  0.999853  0.370403
  Sn  0.833315  0.333175  0.370505
  Sn  0.833316  0.666527  0.370505
  Sn  0.833345  0.999848  0.370399
  Sn  0.166655  0.166621  0.274819
```

Sn	0.166650	0.499896	0.275050
Sn	0.166651	0.833178	0.274819
Sn	0.499986	0.166613	0.274806
Sn	0.499986	0.499901	0.275076
Sn	0.499986	0.833188	0.274805
Sn	0.833311	0.166621	0.274820
Sn	0.833316	0.499896	0.275049
Sn	0.833321	0.833180	0.274820
Sn	0.999765	0.166889	0.715647
Sn	0.000587	0.499877	0.715087
Sn	0.999764	0.832823	0.715646
Sn	0.333550	0.166927	0.715645
Sn	0.333558	0.832819	0.715641
Sn	0.666649	0.167220	0.715556
Sn	0.666657	0.832511	0.715558
Sn	0.000175	0.332934	0.633564
Sn	0.000163	0.666668	0.633557
Sn	0.999946	0.999786	0.633571
Sn	0.333177	0.332961	0.633556
Sn	0.333167	0.666640	0.633553
Sn	0.333371	0.999794	0.633574
Sn	0.666692	0.333023	0.633773
Sn	0.666669	0.666576	0.633772
Sn	0.666629	0.999796	0.633560
Sn	0.999988	0.166543	0.365943
Sn	0.999960	0.499855	0.366232
Sn	0.999987	0.833149	0.365944
Sn	0.333315	0.166535	0.365946
Sn	0.333328	0.499848	0.366236
Sn	0.333316	0.833164	0.365945
Sn	0.666657	0.166536	0.365945
Sn	0.666650	0.499850	0.366234
Sn	0.666654	0.833164	0.365945
Sn	0.999985	0.333243	0.283923
Sn	0.999983	0.666531	0.283929
Sn	0.999988	0.999899	0.283830
Sn	0.333326	0.333251	0.283940
Sn	0.333321	0.666548	0.283938
Sn	0.333316	0.999900	0.283849
Sn	0.666642	0.333250	0.283939
Sn	0.666639	0.666550	0.283938
Sn	0.666657	0.999900	0.283849
Sn	0.166654	0.333178	0.545078

Sn	0.166669	0.666358	0.545075
Sn	0.166635	0.999758	0.544946
Sn	0.500143	0.333117	0.545088
Sn	0.500145	0.666407	0.545086
Sn	0.499975	0.999765	0.544944
Sn	0.833219	0.333127	0.545077
Sn	0.833209	0.666407	0.545076
Sn	0.833341	0.999758	0.544946
Sn	0.166656	0.166481	0.454485
Sn	0.166644	0.499799	0.454852
Sn	0.166653	0.833101	0.454489
Sn	0.499980	0.166482	0.454471
Sn	0.500158	0.499807	0.454962
Sn	0.499984	0.833121	0.454467
Sn	0.833323	0.166476	0.454479
Sn	0.833155	0.499798	0.454957
Sn	0.833326	0.833109	0.454483
Sn	0.999997	0.166439	0.542163
Sn	0.999474	0.499777	0.542759
Sn	0.999998	0.833078	0.542160
Sn	0.333326	0.166436	0.542167
Sn	0.333929	0.499764	0.542757
Sn	0.333342	0.833105	0.542167
Sn	0.666638	0.166419	0.542155
Sn	0.666688	0.499757	0.543133
Sn	0.666627	0.833121	0.542155
Sn	0.999983	0.333113	0.457433
Sn	0.999977	0.666468	0.457439
Sn	0.999988	0.999786	0.457334
Sn	0.333331	0.333131	0.457451
Sn	0.333333	0.666477	0.457450
Sn	0.333307	0.999793	0.457329
Sn	0.666654	0.333111	0.457454
Sn	0.666652	0.666498	0.457449
Sn	0.666657	0.999793	0.457330
O	0.493505	0.563001	0.719422
O	0.840002	0.563038	0.719372
O	0.493521	0.436728	0.719429
O	0.840021	0.436709	0.719409
O	0.333095	0.668303	0.754850
O	0.666705	0.335292	0.754495
O	0.666718	0.664441	0.754493
O	0.666741	0.499785	0.666248

O	0.666685	0.669541	0.686272
O	0.166660	0.104533	0.722643
O	0.166747	0.437898	0.722479
O	0.166676	0.771994	0.723093
O	0.500180	0.104734	0.722692
O	0.500205	0.771358	0.723140
O	0.833121	0.104728	0.722671
O	0.833103	0.771369	0.723177
O	0.166678	0.267172	0.630697
O	0.166663	0.601644	0.630718
O	0.166689	0.934457	0.631346
O	0.499856	0.267314	0.630876
O	0.499933	0.601655	0.631515
O	0.500000	0.934451	0.631309
O	0.833451	0.267280	0.630864
O	0.833452	0.601667	0.631529
O	0.833252	0.934439	0.631317
O	0.166635	0.101055	0.367514
O	0.166638	0.434373	0.367733
O	0.166713	0.767739	0.367560
O	0.499986	0.101070	0.367529
O	0.499986	0.434348	0.367725
O	0.499986	0.767750	0.367612
O	0.833343	0.101056	0.367515
O	0.833319	0.434365	0.367706
O	0.833262	0.767740	0.367559
O	0.166653	0.271560	0.276306
O	0.166700	0.604838	0.276428
O	0.166684	0.938189	0.276282
O	0.499985	0.271568	0.276281
O	0.499979	0.604829	0.276488
O	0.499985	0.938195	0.276300
O	0.833320	0.271560	0.276304
O	0.833262	0.604841	0.276426
O	0.833297	0.938188	0.276282
O	0.166647	0.227754	0.723079
O	0.166737	0.561853	0.722462
O	0.166643	0.895212	0.722617
O	0.500181	0.228374	0.723146
O	0.500199	0.894994	0.722689
O	0.833120	0.228348	0.723169
O	0.833153	0.895003	0.722654
O	0.166675	0.065094	0.631328

O	0.166669	0.397951	0.630701
O	0.166709	0.732416	0.630675
O	0.500001	0.065156	0.631308
O	0.499935	0.397946	0.631495
O	0.499846	0.732291	0.630854
O	0.833276	0.065116	0.631291
O	0.833456	0.397926	0.631512
O	0.833396	0.732307	0.630861
O	0.166716	0.231945	0.367586
O	0.166641	0.565333	0.367750
O	0.166629	0.898630	0.367530
O	0.499983	0.231948	0.367624
O	0.499984	0.565338	0.367730
O	0.499985	0.898623	0.367558
O	0.833262	0.231945	0.367585
O	0.833316	0.565341	0.367725
O	0.833344	0.898631	0.367536
O	0.166692	0.061614	0.276291
O	0.166693	0.394955	0.276423
O	0.166648	0.728241	0.276316
O	0.499983	0.061621	0.276328
O	0.499985	0.394977	0.276482
O	0.499976	0.728224	0.276273
O	0.833282	0.061614	0.276291
O	0.833273	0.394952	0.276425
O	0.833312	0.728242	0.276317
O	0.000304	0.331564	0.754846
O	0.000308	0.668207	0.754841
O	0.999856	0.999879	0.754990
O	0.333086	0.331475	0.754853
O	0.333400	0.999844	0.754999
O	0.666672	0.999839	0.754998
O	0.999885	0.166216	0.663691
O	0.003965	0.499769	0.663129
O	0.999914	0.833338	0.663693
O	0.333409	0.166206	0.663681
O	0.329533	0.499818	0.663125
O	0.333386	0.833398	0.663680
O	0.666641	0.166187	0.663620
O	0.666653	0.833409	0.663625
O	0.999987	0.333058	0.403489
O	0.999983	0.666632	0.403493
O	0.999991	0.999854	0.403419

O	0.333324	0.333096	0.403497
O	0.333319	0.666602	0.403493
O	0.333295	0.999842	0.403399
O	0.666649	0.333092	0.403497
O	0.666649	0.666606	0.403492
O	0.666670	0.999838	0.403399
O	0.999988	0.166657	0.312808
O	0.999994	0.499861	0.313043
O	0.999988	0.833145	0.312807
O	0.333265	0.166669	0.312816
O	0.333254	0.499881	0.313059
O	0.333248	0.833135	0.312817
O	0.666712	0.166668	0.312816
O	0.666701	0.499881	0.313055
O	0.666726	0.833134	0.312818
O	0.001643	0.330715	0.686177
O	0.001670	0.668994	0.686169
O	0.999858	0.999847	0.686413
O	0.331568	0.330764	0.686177
O	0.331590	0.668946	0.686168
O	0.333443	0.999855	0.686423
O	0.666686	0.330137	0.686279
O	0.666650	0.999855	0.686415
O	0.999996	0.166448	0.420278
O	0.999857	0.499771	0.420590
O	0.999997	0.833152	0.420282
O	0.333248	0.166427	0.420283
O	0.333431	0.499819	0.420592
O	0.333215	0.833178	0.420281
O	0.666710	0.166429	0.420283
O	0.666654	0.499820	0.420582
O	0.666751	0.833180	0.420279
O	0.999990	0.333102	0.335615
O	0.999990	0.666583	0.335619
O	0.999982	0.999867	0.335522
O	0.333292	0.333129	0.335623
O	0.333278	0.666586	0.335621
O	0.333326	0.999854	0.335533
O	0.666664	0.333122	0.335622
O	0.666669	0.666591	0.335621
O	0.666651	0.999853	0.335534
O	0.999977	0.166632	0.244338
O	0.999985	0.499933	0.244543

O	0.999987	0.833184	0.244333
O	0.333316	0.166586	0.244324
O	0.333357	0.499904	0.244567
O	0.333327	0.833191	0.244327
O	0.666657	0.166587	0.244324
O	0.666612	0.499906	0.244565
O	0.666645	0.833198	0.244326
O	0.166668	0.101701	0.542692
O	0.166686	0.435137	0.543147
O	0.166771	0.768521	0.542753
O	0.499973	0.101679	0.542703
O	0.500498	0.434951	0.542842
O	0.499996	0.768573	0.542801
O	0.833308	0.101691	0.542697
O	0.832855	0.434980	0.542850
O	0.833220	0.768562	0.542758
O	0.166677	0.268424	0.455864
O	0.166648	0.601872	0.455971
O	0.166656	0.935146	0.455856
O	0.499967	0.268409	0.455881
O	0.500007	0.601907	0.456007
O	0.499974	0.935159	0.455831
O	0.833324	0.268406	0.455855
O	0.833312	0.601896	0.455978
O	0.833311	0.935146	0.455850
O	0.166709	0.231010	0.542759
O	0.166691	0.564398	0.543129
O	0.166673	0.897813	0.542697
O	0.499993	0.230979	0.542805
O	0.500517	0.564557	0.542855
O	0.499977	0.897854	0.542711
O	0.833276	0.230974	0.542774
O	0.832872	0.564555	0.542850
O	0.833307	0.897827	0.542700
O	0.166665	0.064433	0.455872
O	0.166644	0.397725	0.455981
O	0.166656	0.731163	0.455896
O	0.499983	0.064431	0.455836
O	0.500008	0.397700	0.456022
O	0.499963	0.731197	0.455889
O	0.833313	0.064431	0.455871
O	0.833313	0.397696	0.455993
O	0.833332	0.731186	0.455881

O	0.999795	0.332698	0.578683
O	0.999812	0.666835	0.578680
O	0.000001	0.999756	0.578746
O	0.333525	0.332744	0.578666
O	0.333516	0.666791	0.578667
O	0.333238	0.999764	0.578746
O	0.666715	0.332866	0.578786
O	0.666692	0.666679	0.578782
O	0.666710	0.999764	0.578738
O	0.999989	0.166399	0.488602
O	0.000075	0.499817	0.488995
O	0.999987	0.833163	0.488603
O	0.333351	0.166400	0.488606
O	0.333228	0.499781	0.489004
O	0.333362	0.833197	0.488602
O	0.666627	0.166390	0.488600
O	0.666684	0.499784	0.489080
O	0.666616	0.833211	0.488597
O	0.000014	0.165727	0.595667
O	0.997137	0.499799	0.595986
O	0.000001	0.833778	0.595657
O	0.333309	0.165752	0.595668
O	0.336345	0.499808	0.595981
O	0.333299	0.833829	0.595668
O	0.666645	0.165837	0.595654
O	0.666777	0.499775	0.596804
O	0.666649	0.833734	0.595656
O	0.000003	0.332989	0.510453
O	0.999998	0.666576	0.510461
O	0.999991	0.999753	0.510409
O	0.333312	0.333005	0.510469
O	0.333349	0.666523	0.510472
O	0.333267	0.999741	0.510412
O	0.666687	0.332712	0.510470
O	0.666681	0.666820	0.510466
O	0.666694	0.999742	0.510410
O	0.667009	0.499964	0.774153

Structure 5: T-type configuration of O^{2-} on $SnO_2(100)$ surface.

```
_cell_length_a   9.42441600
_cell_length_b   14.11538000
_cell_length_c   32.11538000
_cell_angle_alpha   90.00000000
_cell_angle_beta   90.00000000
_cell_angle_gamma   90.00000000
loop_
 _atom_site_type_symbol
 _atom_site_fract_x
 _atom_site_fract_y
 _atom_site_fract_z
  Sn  0.334772  0.666405  0.682791
  Sn  0.333213  0.324959  0.683661
  Sn  0.666561  0.665964  0.538068
  Sn  0.666685  0.570802  0.705228
  Sn  0.666591  0.332916  0.537185
  Sn  0.666713  0.334015  0.690996
  Sn  0.998651  0.666396  0.682787
  Sn  0.000225  0.324990  0.683649
  Sn  0.166608  0.498505  0.610701
  Sn  0.502074  0.168658  0.611370
  Sn  0.498518  0.829624  0.612272
  Sn  0.498992  0.497374  0.611179
  Sn  0.831180  0.168653  0.611367
  Sn  0.834644  0.829614  0.612267
  Sn  0.834221  0.497356  0.611177
  Sn  0.166602  0.161441  0.316182
  Sn  0.166598  0.494684  0.316139
  Sn  0.166598  0.828337  0.315981
  Sn  0.499940  0.161463  0.316129
  Sn  0.499927  0.494698  0.316098
  Sn  0.499923  0.828320  0.315930
  Sn  0.833272  0.161445  0.316144
  Sn  0.833247  0.494689  0.316110
  Sn  0.833243  0.828325  0.315944
  Sn  0.166589  0.165106  0.463936
  Sn  0.166573  0.499298  0.463807
  Sn  0.166587  0.831888  0.463905
  Sn  0.500187  0.165058  0.463954
  Sn  0.500133  0.499328  0.463834
  Sn  0.500167  0.831902  0.464023
```

Sn	0.832994	0.165058	0.463941
Sn	0.833005	0.499307	0.463831
Sn	0.832969	0.831850	0.464013
Sn	0.166617	0.164171	0.609089
Sn	0.166560	0.833833	0.609482
Sn	0.999895	0.332243	0.390497
Sn	0.999856	0.665787	0.390299
Sn	0.999860	0.998661	0.390450
Sn	0.333333	0.332239	0.390503
Sn	0.333326	0.665805	0.390292
Sn	0.333350	0.998711	0.390437
Sn	0.666574	0.332225	0.390459
Sn	0.666552	0.665800	0.390253
Sn	0.666584	0.998713	0.390396
Sn	0.999921	0.332569	0.536872
Sn	0.999753	0.665759	0.537491
Sn	0.998523	0.998579	0.537411
Sn	0.333284	0.332585	0.536872
Sn	0.333397	0.665784	0.537496
Sn	0.334609	0.998583	0.537424
Sn	0.666571	0.998104	0.538232
Sn	0.000114	0.995684	0.683635
Sn	0.333258	0.995696	0.683624
Sn	0.666674	0.994138	0.684304
O	0.332273	0.434718	0.580513
O	0.666610	0.102140	0.581720
O	0.666594	0.766975	0.582340
O	0.666595	0.435294	0.581043
O	0.332363	0.566007	0.637263
O	0.330177	0.232755	0.635971
O	0.666632	0.559188	0.641522
O	0.666666	0.237117	0.638363
O	0.166757	0.739634	0.654523
O	0.166687	0.402664	0.656717
O	0.500553	0.071730	0.657048
O	0.481787	0.743677	0.658511
O	0.492179	0.400682	0.656113
O	0.832683	0.071669	0.657023
O	0.851732	0.743722	0.658509
O	0.841155	0.400638	0.656092
O	0.166707	0.629414	0.715644
O	0.166725	0.285726	0.716416
O	0.500425	0.599658	0.565270

O	0.480993	0.614365	0.720250
O	0.499979	0.266973	0.564118
O	0.493292	0.272736	0.714109
O	0.832694	0.599628	0.565264
O	0.852414	0.614333	0.720238
O	0.833224	0.266990	0.564121
O	0.840188	0.272804	0.714116
O	0.166620	0.065022	0.361569
O	0.166655	0.398409	0.361564
O	0.166616	0.731872	0.361365
O	0.499925	0.064981	0.361477
O	0.499938	0.398377	0.361492
O	0.499916	0.731766	0.361252
O	0.833251	0.064977	0.361498
O	0.833205	0.398395	0.361513
O	0.833203	0.731780	0.361277
O	0.166598	0.063370	0.507827
O	0.166594	0.396139	0.507103
O	0.166627	0.728635	0.507092
O	0.500906	0.062610	0.507781
O	0.500101	0.396482	0.507369
O	0.500567	0.728838	0.507640
O	0.832201	0.062649	0.507787
O	0.833050	0.396446	0.507364
O	0.832528	0.728799	0.507632
O	0.166624	0.074123	0.657628
O	0.166623	0.266128	0.417716
O	0.166618	0.599775	0.417575
O	0.166664	0.932638	0.417628
O	0.500006	0.266153	0.417705
O	0.499991	0.599718	0.417528
O	0.500052	0.932739	0.417634
O	0.833172	0.266160	0.417683
O	0.833119	0.599741	0.417537
O	0.833069	0.932684	0.417635
O	0.166636	0.265551	0.563318
O	0.166585	0.599606	0.564337
O	0.166570	0.933813	0.563590
O	0.500516	0.931859	0.564799
O	0.832621	0.931890	0.564810
O	0.166752	0.955525	0.716605
O	0.498751	0.957314	0.717345
O	0.834641	0.957376	0.717368

O	0.999975	0.122171	0.282802
O	0.999952	0.455424	0.282783
O	0.999964	0.789420	0.282544
O	0.333201	0.122378	0.282743
O	0.333214	0.455493	0.282748
O	0.333194	0.789345	0.282537
O	0.666638	0.122359	0.282719
O	0.666606	0.455487	0.282723
O	0.666593	0.789318	0.282512
O	0.999762	0.100936	0.434384
O	0.999778	0.434963	0.434278
O	0.999601	0.767965	0.434234
O	0.333419	0.101007	0.434368
O	0.333380	0.435030	0.434257
O	0.333594	0.768012	0.434228
O	0.666611	0.101219	0.434250
O	0.666580	0.435003	0.434235
O	0.666531	0.767991	0.434188
O	0.997759	0.100832	0.581212
O	0.000917	0.434700	0.580513
O	0.000666	0.770371	0.579586
O	0.335488	0.100801	0.581231
O	0.332442	0.770367	0.579608
O	0.999936	0.238832	0.342459
O	0.999939	0.572160	0.342383
O	0.999927	0.905377	0.342397
O	0.333270	0.238770	0.342483
O	0.333230	0.572119	0.342396
O	0.333279	0.905407	0.342383
O	0.666599	0.238782	0.342438
O	0.666589	0.572106	0.342366
O	0.666575	0.905408	0.342342
O	0.999505	0.231369	0.490837
O	0.999579	0.564698	0.491191
O	0.999645	0.898034	0.490905
O	0.333649	0.231367	0.490854
O	0.333522	0.564696	0.491203
O	0.333440	0.898027	0.490935
O	0.666605	0.231484	0.491018
O	0.666587	0.564768	0.491354
O	0.666627	0.897722	0.491183
O	0.003071	0.232781	0.635966
O	0.000836	0.566011	0.637247

O	0.002666	0.906421	0.635537
O	0.330486	0.906425	0.635531
O	0.666620	0.900492	0.637708
O	0.666661	0.445648	0.727127

Structure 6: T-type configuration of O^- on $SnO_2(101)$ surface.

_cell_length_a 16.97242900
_cell_length_b 14.11702300
_cell_length_c 33.67701300
_cell_angle_alpha 90.00000000
_cell_angle_beta 90.00000000
_cell_angle_gamma 90.00000000
loop_
_atom_site_type_symbol
_atom_site_fract_x
_atom_site_fract_y
_atom_site_fract_z
Sn 0.382458 0.178085 0.688788
Sn 0.388286 0.508056 0.697121
Sn 0.489234 0.498301 0.614140
Sn 0.323559 0.336501 0.613373
Sn 0.218413 0.319476 0.689201
Sn 0.548985 0.322714 0.689883
Sn 0.217252 0.653422 0.689711
Sn 0.551207 0.656011 0.689396
Sn 0.157295 0.163815 0.612690
Sn 0.157488 0.497637 0.613136
Sn 0.158389 0.829442 0.612924
Sn 0.490875 0.164503 0.612986
Sn 0.490650 0.831316 0.612586
Sn 0.824094 0.164143 0.612860
Sn 0.823994 0.497424 0.612752
Sn 0.824121 0.830908 0.612730
Sn 0.029739 0.165651 0.457387
Sn 0.029578 0.499059 0.457327
Sn 0.029691 0.832324 0.457334
Sn 0.362950 0.166031 0.457394
Sn 0.362875 0.499113 0.457522
Sn 0.362976 0.832189 0.457536
Sn 0.696218 0.165904 0.457373
Sn 0.696018 0.499022 0.457586
Sn 0.696265 0.832446 0.457334
Sn 0.239107 0.153216 0.302836
Sn 0.239054 0.486577 0.302808
Sn 0.239092 0.819906 0.302842
Sn 0.572388 0.153311 0.302835
Sn 0.572365 0.486602 0.302907

Sn	0.572376	0.819867	0.302907
Sn	0.905731	0.153252	0.302811
Sn	0.905683	0.486499	0.302885
Sn	0.905703	0.819897	0.302808
Sn	0.050787	0.180402	0.689110
Sn	0.050414	0.513549	0.689147
Sn	0.050520	0.846748	0.689042
Sn	0.383948	0.846356	0.688943
Sn	0.715924	0.181620	0.689105
Sn	0.717008	0.513320	0.689057
Sn	0.717197	0.847396	0.689068
Sn	0.259862	0.167835	0.534895
Sn	0.259797	0.500904	0.535147
Sn	0.260250	0.832992	0.535263
Sn	0.593036	0.167937	0.534915
Sn	0.592671	0.500680	0.535426
Sn	0.593210	0.834264	0.534838
Sn	0.926580	0.167467	0.534873
Sn	0.926422	0.500851	0.534820
Sn	0.926547	0.834189	0.534795
Sn	0.132197	0.169019	0.379411
Sn	0.132077	0.502473	0.379345
Sn	0.132178	0.835746	0.379384
Sn	0.465457	0.169216	0.379402
Sn	0.465386	0.502483	0.379477
Sn	0.465443	0.835679	0.379494
Sn	0.798774	0.169148	0.379370
Sn	0.798661	0.502394	0.379481
Sn	0.798780	0.835783	0.379364
Sn	0.217112	0.986220	0.689047
Sn	0.549796	0.986327	0.688941
Sn	0.883711	0.987003	0.689050
Sn	0.883817	0.320050	0.689057
Sn	0.883642	0.653194	0.689032
Sn	0.093528	0.998599	0.534876
Sn	0.093231	0.332545	0.534879
Sn	0.093206	0.665529	0.534887
Sn	0.426472	0.999391	0.534805
Sn	0.426157	0.332795	0.535276
Sn	0.426442	0.665191	0.535369
Sn	0.759771	0.999217	0.534795
Sn	0.759612	0.332443	0.535019
Sn	0.759720	0.665764	0.534860

Sn	0.298756	0.330913	0.379389
Sn	0.298802	0.664099	0.379434
Sn	0.298876	0.997401	0.379432
Sn	0.632011	0.330870	0.379473
Sn	0.632056	0.664071	0.379518
Sn	0.632083	0.997508	0.379370
Sn	0.965457	0.330741	0.379396
Sn	0.965427	0.664130	0.379355
Sn	0.965480	0.997446	0.379343
Sn	0.324055	0.002297	0.612493
Sn	0.325915	0.666044	0.614477
Sn	0.657512	0.003222	0.612790
Sn	0.656679	0.336420	0.613108
Sn	0.657267	0.669447	0.612787
Sn	0.991024	0.002316	0.612776
Sn	0.990846	0.335934	0.612764
Sn	0.990688	0.669177	0.612733
Sn	0.196584	0.000548	0.457460
Sn	0.196212	0.334241	0.457359
Sn	0.196307	0.667397	0.457405
Sn	0.529598	0.000965	0.457360
Sn	0.529369	0.334287	0.457543
Sn	0.529534	0.667297	0.457624
Sn	0.862983	0.000902	0.457315
Sn	0.862862	0.334096	0.457410
Sn	0.862904	0.667487	0.457346
Sn	0.072443	0.013369	0.302814
Sn	0.072386	0.346619	0.302816
Sn	0.072386	0.680041	0.302806
Sn	0.405787	0.013318	0.302877
Sn	0.405714	0.346749	0.302835
Sn	0.405726	0.679977	0.302881
Sn	0.739062	0.013405	0.302829
Sn	0.738988	0.346714	0.302870
Sn	0.739013	0.679917	0.302910
O	0.457438	0.263741	0.714463
O	0.458057	0.615249	0.715625
O	0.313402	0.395490	0.668387
O	0.651200	0.397346	0.668970
O	0.486774	0.434704	0.667889
O	0.288188	0.224738	0.714365
O	0.623127	0.232209	0.715169
O	0.290182	0.569133	0.715456

O	0.624550	0.564757	0.714531
O	0.393565	0.230704	0.633756
O	0.393897	0.562600	0.637892
O	0.226114	0.267253	0.633673
O	0.225987	0.602881	0.634099
O	0.227183	0.934745	0.633827
O	0.561382	0.267428	0.634728
O	0.560972	0.603561	0.634118
O	0.561369	0.935499	0.633724
O	0.893974	0.268421	0.633901
O	0.893718	0.602148	0.633757
O	0.894159	0.935110	0.633925
O	0.099169	0.269692	0.480358
O	0.099255	0.602803	0.480351
O	0.099474	0.935972	0.480397
O	0.432549	0.269643	0.480629
O	0.432725	0.602550	0.480714
O	0.432753	0.936179	0.480423
O	0.765908	0.269587	0.480496
O	0.766013	0.602851	0.480438
O	0.766056	0.936264	0.480361
O	0.306871	0.269043	0.323094
O	0.306918	0.602291	0.323142
O	0.306950	0.935587	0.323136
O	0.640143	0.269063	0.323139
O	0.640191	0.602271	0.323218
O	0.640221	0.935663	0.323104
O	0.973507	0.268966	0.323097
O	0.973548	0.602317	0.323084
O	0.973573	0.935616	0.323064
O	0.123792	0.270370	0.714544
O	0.121859	0.604592	0.714555
O	0.123536	0.936524	0.714886
O	0.456742	0.936486	0.715020
O	0.790467	0.269971	0.714846
O	0.790350	0.603504	0.714747
O	0.790610	0.936569	0.714974
O	0.328972	0.270624	0.558429
O	0.330153	0.602189	0.559188
O	0.329594	0.936754	0.558099
O	0.662569	0.270641	0.558407
O	0.662762	0.603951	0.558283
O	0.662889	0.937349	0.558230

O	0.995947	0.270547	0.558190
O	0.995727	0.604023	0.558103
O	0.996153	0.936975	0.558198
O	0.202013	0.269415	0.402908
O	0.201999	0.602636	0.402967
O	0.202104	0.935905	0.402983
O	0.535288	0.269427	0.403022
O	0.535284	0.602655	0.403104
O	0.535350	0.936040	0.402968
O	0.868666	0.269346	0.402946
O	0.868588	0.602761	0.402927
O	0.868674	0.936038	0.402909
O	0.087297	0.063960	0.588741
O	0.087169	0.397571	0.588826
O	0.087182	0.730686	0.588853
O	0.420568	0.064665	0.588522
O	0.419848	0.398037	0.588974
O	0.420998	0.729792	0.589307
O	0.754079	0.064421	0.588778
O	0.753814	0.397514	0.588948
O	0.753974	0.731101	0.588821
O	0.293298	0.063007	0.433523
O	0.293042	0.396582	0.433483
O	0.293200	0.729620	0.433556
O	0.626516	0.063161	0.433479
O	0.626347	0.396393	0.433619
O	0.626472	0.729615	0.433645
O	0.959873	0.063065	0.433454
O	0.959783	0.396323	0.433518
O	0.959846	0.729675	0.433499
O	0.165834	0.063402	0.276967
O	0.165757	0.396777	0.276972
O	0.165748	0.730207	0.276973
O	0.499149	0.063410	0.277015
O	0.499115	0.396859	0.277013
O	0.499090	0.730122	0.277047
O	0.832446	0.063440	0.276968
O	0.832380	0.396775	0.277019
O	0.832351	0.730087	0.277033
O	0.315320	0.063439	0.668129
O	0.315103	0.734367	0.668319
O	0.648979	0.065214	0.668796
O	0.650059	0.732590	0.668628

O	0.982372	0.064473	0.668685
O	0.982309	0.397434	0.668677
O	0.982173	0.731194	0.668654
O	0.189806	0.064009	0.511307
O	0.189607	0.397577	0.511259
O	0.189688	0.730457	0.511343
O	0.523068	0.064388	0.511270
O	0.522816	0.397513	0.511508
O	0.523004	0.730644	0.511515
O	0.856405	0.064127	0.511305
O	0.856337	0.397304	0.511403
O	0.856383	0.730767	0.511326
O	0.062086	0.064986	0.357814
O	0.062000	0.398349	0.357810
O	0.062099	0.731605	0.357813
O	0.395429	0.065046	0.357860
O	0.395324	0.398465	0.357832
O	0.395454	0.731549	0.357890
O	0.728705	0.065066	0.357826
O	0.728630	0.398353	0.357887
O	0.728743	0.731544	0.357914
O	0.148531	0.101368	0.668578
O	0.148033	0.435556	0.668727
O	0.148834	0.769144	0.668930
O	0.481733	0.100103	0.668125
O	0.483682	0.772402	0.667945
O	0.815589	0.102456	0.668769
O	0.816506	0.435948	0.668697
O	0.816039	0.769438	0.668648
O	0.023157	0.102271	0.511347
O	0.022964	0.435852	0.511301
O	0.023094	0.769119	0.511307
O	0.356296	0.102631	0.511247
O	0.356054	0.435650	0.511328
O	0.356564	0.768400	0.511464
O	0.689727	0.102782	0.511347
O	0.689518	0.435864	0.511547
O	0.689855	0.769355	0.511309
O	0.228764	0.101491	0.357835
O	0.228655	0.434903	0.357788
O	0.228790	0.768242	0.357839
O	0.562041	0.101634	0.357839
O	0.561981	0.434906	0.357894

O	0.562112	0.768285	0.357930
O	0.895370	0.101551	0.357810
O	0.895317	0.434849	0.357879
O	0.895444	0.768348	0.357824
O	0.253587	0.101746	0.588546
O	0.253090	0.435518	0.588758
O	0.254872	0.767478	0.589060
O	0.587316	0.102660	0.588735
O	0.586720	0.436281	0.589268
O	0.587698	0.769863	0.588723
O	0.920693	0.102319	0.588865
O	0.920571	0.435804	0.588818
O	0.920674	0.769318	0.588814
O	0.126637	0.103386	0.433522
O	0.126411	0.436853	0.433430
O	0.126560	0.770098	0.433486
O	0.459845	0.103677	0.433510
O	0.459683	0.436807	0.433554
O	0.459874	0.770070	0.433640
O	0.793169	0.103616	0.433503
O	0.793030	0.436829	0.433610
O	0.793218	0.770281	0.433516
O	0.332505	0.103093	0.277013
O	0.332450	0.436549	0.276962
O	0.332442	0.769728	0.277013
O	0.665793	0.103223	0.276997
O	0.665746	0.436578	0.277039
O	0.665724	0.769688	0.277066
O	0.999133	0.103147	0.276977
O	0.999026	0.436385	0.276998
O	0.999048	0.769759	0.276953
O	0.059997	0.230852	0.633758
O	0.059736	0.564528	0.633748
O	0.060943	0.897940	0.633819
O	0.394335	0.899610	0.633804
O	0.727460	0.231789	0.633860
O	0.727797	0.565483	0.633924
O	0.727936	0.899303	0.633976
O	0.265846	0.230421	0.480358
O	0.266118	0.564030	0.480658
O	0.266144	0.896575	0.480638
O	0.599290	0.230557	0.480449
O	0.599202	0.563635	0.480744

O	0.599444	0.897231	0.480411
O	0.932625	0.230230	0.480402
O	0.932585	0.563674	0.480350
O	0.932758	0.897106	0.480365
O	0.140208	0.230856	0.323092
O	0.140205	0.564323	0.323065
O	0.140290	0.897595	0.323111
O	0.473526	0.230982	0.323107
O	0.473547	0.564299	0.323179
O	0.473600	0.897558	0.323176
O	0.806834	0.230929	0.323100
O	0.806832	0.564224	0.323170
O	0.806904	0.897615	0.323092
O	0.290783	0.897775	0.714876
O	0.623865	0.897647	0.714783
O	0.956989	0.229979	0.714779
O	0.956259	0.562750	0.714609
O	0.956963	0.896947	0.714702
O	0.162292	0.229109	0.558020
O	0.162372	0.562723	0.558276
O	0.162969	0.895425	0.558327
O	0.496160	0.229966	0.558423
O	0.495825	0.562849	0.558885
O	0.496009	0.896299	0.558084
O	0.829431	0.229460	0.558280
O	0.829349	0.562874	0.558195
O	0.829503	0.896358	0.558199
O	0.035380	0.230446	0.402919
O	0.035270	0.563870	0.402896
O	0.035340	0.897137	0.402913
O	0.368687	0.230691	0.402942
O	0.368646	0.563941	0.403052
O	0.368680	0.897069	0.403030
O	0.702026	0.230632	0.402949
O	0.701901	0.563828	0.403088
O	0.701965	0.897198	0.402916
O	0.413281	0.424469	0.742316

Structure 7: B-type configuration of O^- on $SnO_2(110)$ surface.

```
_cell_length_a   9.42435700
_cell_length_b   19.96585700
_cell_length_c   37.96585700
_cell_angle_alpha   90.00000000
_cell_angle_beta   90.00000000
_cell_angle_gamma   90.00000000
loop_
 _atom_site_type_symbol
 _atom_site_fract_x
 _atom_site_fract_y
 _atom_site_fract_z
  Sn  0.499975  0.665865  0.722456
  Sn  0.832971  0.334254  0.724563
  Sn  0.166973  0.334254  0.724562
  Sn  0.499986  0.500165  0.632272
  Sn  0.341482  0.501316  0.719225
  Sn  0.658467  0.501313  0.719225
  Sn  0.166669  0.665839  0.724542
  Sn  0.166632  0.999765  0.724499
  Sn  0.499970  0.332479  0.723403
  Sn  0.499967  0.999784  0.724532
  Sn  0.833282  0.665838  0.724541
  Sn  0.833303  0.999764  0.724498
  Sn  0.166496  0.166446  0.629038
  Sn  0.167660  0.499801  0.630011
  Sn  0.166462  0.833283  0.629024
  Sn  0.499976  0.166502  0.628993
  Sn  0.499982  0.833260  0.628957
  Sn  0.833454  0.166447  0.629039
  Sn  0.832311  0.499803  0.630012
  Sn  0.833504  0.833285  0.629024
  Sn  0.166675  0.333186  0.370520
  Sn  0.166678  0.666543  0.370482
  Sn  0.166644  0.999862  0.370350
  Sn  0.499996  0.333182  0.370535
  Sn  0.499997  0.666543  0.370492
  Sn  0.499999  0.999874  0.370345
  Sn  0.833319  0.333187  0.370520
  Sn  0.833317  0.666543  0.370482
  Sn  0.833353  0.999862  0.370351
  Sn  0.166668  0.166647  0.274794
```

Sn	0.166666	0.499895	0.275176
Sn	0.166669	0.833185	0.274779
Sn	0.500000	0.166635	0.274776
Sn	0.499998	0.499899	0.275200
Sn	0.500000	0.833191	0.274755
Sn	0.833332	0.166647	0.274794
Sn	0.833330	0.499896	0.275177
Sn	0.833330	0.833185	0.274779
Sn	0.999968	0.166858	0.715779
Sn	0.999976	0.499459	0.715971
Sn	0.999969	0.832818	0.715837
Sn	0.332955	0.166715	0.715550
Sn	0.333279	0.832696	0.715528
Sn	0.666977	0.166716	0.715551
Sn	0.666653	0.832692	0.715528
Sn	0.999977	0.332964	0.633546
Sn	0.999982	0.666757	0.633515
Sn	0.999984	0.999868	0.633535
Sn	0.333000	0.332861	0.633491
Sn	0.332840	0.667040	0.633330
Sn	0.333328	0.999870	0.633536
Sn	0.666961	0.332862	0.633491
Sn	0.667125	0.667044	0.633330
Sn	0.666642	0.999871	0.633537
Sn	0.999997	0.166557	0.365924
Sn	0.999997	0.499870	0.366369
Sn	0.999997	0.833164	0.365909
Sn	0.333326	0.166552	0.365918
Sn	0.333350	0.499860	0.366381
Sn	0.333327	0.833184	0.365903
Sn	0.666670	0.166552	0.365917
Sn	0.666641	0.499860	0.366382
Sn	0.666669	0.833184	0.365903
Sn	0.000000	0.333260	0.283937
Sn	0.999998	0.666539	0.283913
Sn	0.000000	0.999914	0.283776
Sn	0.333342	0.333260	0.283956
Sn	0.333337	0.666552	0.283919
Sn	0.333332	0.999915	0.283786
Sn	0.666657	0.333261	0.283956
Sn	0.666658	0.666552	0.283919
Sn	0.666668	0.999915	0.283786
Sn	0.166608	0.333201	0.545060

Sn	0.166549	0.666453	0.544989
Sn	0.166643	0.999826	0.544905
Sn	0.499990	0.333185	0.545054
Sn	0.499992	0.666454	0.544994
Sn	0.499991	0.999819	0.544908
Sn	0.833370	0.333201	0.545060
Sn	0.833434	0.666454	0.544990
Sn	0.833336	0.999827	0.544905
Sn	0.166665	0.166501	0.454462
Sn	0.166776	0.499826	0.455060
Sn	0.166667	0.833139	0.454448
Sn	0.499992	0.166511	0.454451
Sn	0.499993	0.499835	0.455189
Sn	0.499996	0.833141	0.454437
Sn	0.833318	0.166501	0.454461
Sn	0.833211	0.499827	0.455060
Sn	0.833325	0.833139	0.454448
Sn	0.999987	0.166453	0.542143
Sn	0.999991	0.499833	0.542955
Sn	0.999994	0.833240	0.542130
Sn	0.333323	0.166476	0.542145
Sn	0.333725	0.499825	0.543285
Sn	0.333318	0.833183	0.542132
Sn	0.666654	0.166476	0.542145
Sn	0.666257	0.499826	0.543284
Sn	0.666670	0.833183	0.542133
Sn	0.999994	0.333132	0.457422
Sn	0.999995	0.666522	0.457367
Sn	0.999996	0.999810	0.457289
Sn	0.333324	0.333147	0.457458
Sn	0.333325	0.666520	0.457409
Sn	0.333331	0.999821	0.457284
Sn	0.666663	0.333148	0.457458
Sn	0.666666	0.666521	0.457409
Sn	0.666660	0.999821	0.457284
O	0.499986	0.567695	0.708825
O	0.833676	0.561800	0.723243
O	0.166278	0.561802	0.723243
O	0.499984	0.431898	0.709605
O	0.831527	0.438519	0.723424
O	0.168420	0.438519	0.723421
O	0.335171	0.333867	0.754342
O	0.336078	0.663453	0.753970

O	0.664762	0.333881	0.754341
O	0.663875	0.663452	0.753969
O	0.327614	0.499473	0.664799
O	0.672351	0.499471	0.664799
O	0.329237	0.673245	0.685702
O	0.670718	0.673255	0.685703
O	0.330234	0.327342	0.685930
O	0.166521	0.104449	0.722653
O	0.166734	0.771788	0.723999
O	0.499951	0.104470	0.722642
O	0.499966	0.771592	0.723435
O	0.833414	0.104448	0.722653
O	0.833208	0.771787	0.723998
O	0.165849	0.267103	0.630603
O	0.166306	0.601896	0.631189
O	0.166631	0.934554	0.631401
O	0.499979	0.267205	0.630674
O	0.499988	0.602301	0.631275
O	0.499987	0.934549	0.631345
O	0.834102	0.267103	0.630602
O	0.833654	0.601899	0.631191
O	0.833333	0.934555	0.631400
O	0.166650	0.101069	0.367483
O	0.166663	0.434379	0.367819
O	0.166713	0.767759	0.367506
O	0.499998	0.101095	0.367478
O	0.499996	0.434351	0.367853
O	0.499998	0.767772	0.367543
O	0.833347	0.101069	0.367483
O	0.833331	0.434380	0.367819
O	0.833281	0.767760	0.367507
O	0.166660	0.271578	0.276283
O	0.166713	0.604838	0.276439
O	0.166712	0.938203	0.276207
O	0.500000	0.271583	0.276266
O	0.499997	0.604830	0.276497
O	0.500000	0.938204	0.276221
O	0.833339	0.271578	0.276283
O	0.833285	0.604839	0.276443
O	0.833288	0.938203	0.276207
O	0.166429	0.227956	0.723615
O	0.166707	0.895133	0.722472
O	0.499969	0.227518	0.723335

O	0.499960	0.894992	0.722563
O	0.833514	0.227956	0.723620
O	0.833237	0.895131	0.722472
O	0.166649	0.065155	0.631340
O	0.166298	0.397752	0.631101
O	0.165606	0.732647	0.630334
O	0.499981	0.065200	0.631311
O	0.499981	0.397770	0.631626
O	0.499983	0.732627	0.630503
O	0.833314	0.065157	0.631343
O	0.833662	0.397753	0.631100
O	0.834357	0.732649	0.630335
O	0.166720	0.231953	0.367551
O	0.166668	0.565357	0.367822
O	0.166642	0.898648	0.367480
O	0.499998	0.231960	0.367575
O	0.499996	0.565363	0.367818
O	0.499998	0.898646	0.367479
O	0.833270	0.231955	0.367552
O	0.833326	0.565357	0.367822
O	0.833356	0.898649	0.367480
O	0.166704	0.061632	0.276220
O	0.166708	0.394961	0.276462
O	0.166657	0.728245	0.276267
O	0.500000	0.061637	0.276257
O	0.499999	0.394982	0.276514
O	0.499998	0.728222	0.276222
O	0.833295	0.061632	0.276220
O	0.833290	0.394961	0.276463
O	0.833340	0.728244	0.276266
O	0.999975	0.332502	0.754892
O	0.999977	0.668033	0.754974
O	0.999964	0.999622	0.754958
O	0.333254	0.999659	0.754969
O	0.666679	0.999662	0.754968
O	0.999971	0.166416	0.663807
O	0.999981	0.499672	0.664020
O	0.999980	0.833087	0.663851
O	0.333245	0.166143	0.663649
O	0.333218	0.833415	0.663630
O	0.666701	0.166147	0.663650
O	0.666745	0.833417	0.663631
O	0.999997	0.333008	0.403491

O	0.999996	0.666707	0.403449
O	0.999999	0.999858	0.403371
O	0.333339	0.333031	0.403508
O	0.333348	0.666668	0.403466
O	0.333328	0.999844	0.403345
O	0.666653	0.333034	0.403508
O	0.666647	0.666668	0.403466
O	0.666668	0.999844	0.403345
O	0.000001	0.166662	0.312778
O	0.999998	0.499885	0.313165
O	0.000001	0.833166	0.312764
O	0.333252	0.166666	0.312780
O	0.333322	0.499869	0.313175
O	0.333253	0.833148	0.312765
O	0.666747	0.166666	0.312780
O	0.666673	0.499873	0.313175
O	0.666746	0.833148	0.312765
O	0.999966	0.332680	0.686413
O	0.999976	0.667833	0.686387
O	0.999976	0.999916	0.686384
O	0.333344	0.999920	0.686396
O	0.669720	0.327336	0.685930
O	0.666610	0.999919	0.686396
O	0.999995	0.166457	0.420256
O	0.999993	0.499803	0.420712
O	0.999997	0.833185	0.420235
O	0.333282	0.166457	0.420253
O	0.333452	0.499842	0.420733
O	0.333254	0.833217	0.420241
O	0.666706	0.166456	0.420252
O	0.666538	0.499841	0.420732
O	0.666741	0.833219	0.420241
O	0.999999	0.333158	0.335624
O	0.000003	0.666575	0.335594
O	0.000002	0.999872	0.335470
O	0.333325	0.333172	0.335635
O	0.333297	0.666564	0.335600
O	0.333330	0.999890	0.335472
O	0.666666	0.333174	0.335635
O	0.666699	0.666565	0.335600
O	0.666668	0.999890	0.335472
O	0.000000	0.166699	0.244304
O	0.999997	0.499897	0.244657

O	0.000000	0.833174	0.244288
O	0.333329	0.166617	0.244288
O	0.333360	0.499903	0.244688
O	0.333340	0.833189	0.244272
O	0.666671	0.166617	0.244288
O	0.666636	0.499903	0.244688
O	0.666659	0.833189	0.244272
O	0.166708	0.101727	0.542659
O	0.166773	0.435128	0.543210
O	0.166638	0.768669	0.542744
O	0.499984	0.101728	0.542696
O	0.499986	0.434955	0.542892
O	0.499994	0.768655	0.542794
O	0.833265	0.101726	0.542655
O	0.833206	0.435129	0.543211
O	0.833350	0.768670	0.542744
O	0.166675	0.268422	0.455849
O	0.166648	0.601934	0.455970
O	0.166688	0.935183	0.455803
O	0.499990	0.268426	0.455886
O	0.499995	0.601960	0.456033
O	0.499996	0.935186	0.455796
O	0.833316	0.268423	0.455847
O	0.833343	0.601935	0.455969
O	0.833305	0.935184	0.455803
O	0.166671	0.231003	0.542756
O	0.166770	0.564511	0.543131
O	0.166723	0.897940	0.542629
O	0.499990	0.231015	0.542793
O	0.499991	0.564698	0.542854
O	0.499995	0.897917	0.542683
O	0.833306	0.231004	0.542756
O	0.833210	0.564513	0.543134
O	0.833261	0.897940	0.542630
O	0.166675	0.064450	0.455822
O	0.166658	0.397716	0.456005
O	0.166678	0.731239	0.455817
O	0.499996	0.064459	0.455789
O	0.499992	0.397710	0.456066
O	0.499996	0.731242	0.455832
O	0.833315	0.064450	0.455821
O	0.833331	0.397717	0.456005
O	0.833312	0.731239	0.455817

O	0.999986	0.332702	0.578620
O	0.999990	0.666790	0.578563
O	0.999985	0.999818	0.578727
O	0.333361	0.332755	0.578668
O	0.333237	0.666921	0.578573
O	0.333246	0.999807	0.578729
O	0.666619	0.332752	0.578668
O	0.666742	0.666922	0.578572
O	0.666737	0.999812	0.578730
O	0.999987	0.166395	0.488581
O	0.999992	0.499825	0.489135
O	0.999996	0.833234	0.488559
O	0.333359	0.166397	0.488584
O	0.333150	0.499810	0.489247
O	0.333373	0.833230	0.488571
O	0.666625	0.166399	0.488584
O	0.666842	0.499811	0.489246
O	0.666619	0.833229	0.488570
O	0.999977	0.165415	0.595651
O	0.999990	0.499884	0.596379
O	0.999988	0.834494	0.595639
O	0.333227	0.165739	0.595654
O	0.335324	0.499921	0.596661
O	0.333178	0.834100	0.595638
O	0.666737	0.165740	0.595654
O	0.664652	0.499926	0.596661
O	0.666788	0.834099	0.595639
O	0.999990	0.333043	0.510423
O	0.999992	0.666658	0.510358
O	0.999990	0.999816	0.510370
O	0.333217	0.332882	0.510468
O	0.333215	0.666780	0.510412
O	0.333305	0.999813	0.510375
O	0.666767	0.332882	0.510467
O	0.666773	0.666781	0.510411
O	0.666682	0.999812	0.510376
O	0.499937	0.486783	0.759830

Structure 8: B-type configuration of O^- on SnO_2(100) surface.

_cell_length_a 9.42441600
_cell_length_b 14.11538000
_cell_length_c 32.11538000
_cell_angle_alpha 90.00000000
_cell_angle_beta 90.00000000
_cell_angle_gamma 90.00000000
loop_
_atom_site_type_symbol
_atom_site_fract_x
_atom_site_fract_y
_atom_site_fract_z
Sn 0.000076 0.660949 0.683845
Sn 0.333004 0.332456 0.536312
Sn 0.000103 0.328467 0.684384
Sn 0.333768 0.660274 0.683891
Sn 0.342437 0.328105 0.687907
Sn 0.666379 0.660275 0.683888
Sn 0.667141 0.332464 0.536309
Sn 0.657780 0.328101 0.687907
Sn 0.167159 0.167364 0.609988
Sn 0.166939 0.497106 0.610738
Sn 0.500104 0.171123 0.611476
Sn 0.500069 0.495103 0.609470
Sn 0.833055 0.167359 0.609986
Sn 0.833198 0.497103 0.610737
Sn 0.166717 0.161005 0.315680
Sn 0.166729 0.495553 0.315653
Sn 0.166710 0.828301 0.315651
Sn 0.500043 0.161003 0.315657
Sn 0.500061 0.495552 0.315650
Sn 0.500030 0.828280 0.315636
Sn 0.833370 0.161009 0.315681
Sn 0.833394 0.495556 0.315653
Sn 0.833352 0.828301 0.315648
Sn 0.166652 0.165727 0.463152
Sn 0.166680 0.499237 0.463107
Sn 0.166831 0.832447 0.463790
Sn 0.500061 0.165665 0.463144
Sn 0.500049 0.499123 0.463176
Sn 0.500057 0.832701 0.463896
Sn 0.833469 0.165725 0.463154

Sn	0.833424	0.499247	0.463106
Sn	0.833288	0.832446	0.463791
Sn	0.166888	0.832547	0.610018
Sn	0.500070	0.832778	0.610044
Sn	0.833263	0.832546	0.610017
Sn	0.000051	0.332642	0.389683
Sn	0.000047	0.666466	0.390044
Sn	0.000055	0.998790	0.390018
Sn	0.333396	0.332624	0.389688
Sn	0.333438	0.666426	0.390077
Sn	0.333387	0.998840	0.390018
Sn	0.666708	0.332623	0.389690
Sn	0.666649	0.666435	0.390074
Sn	0.666723	0.998835	0.390019
Sn	0.000069	0.332912	0.536235
Sn	0.000067	0.664945	0.537158
Sn	0.000080	0.999891	0.536948
Sn	0.333330	0.664890	0.537069
Sn	0.333671	0.000559	0.537198
Sn	0.666798	0.664890	0.537071
Sn	0.666479	0.000554	0.537201
Sn	0.000102	0.995281	0.684004
Sn	0.333605	0.996421	0.683773
Sn	0.666588	0.996435	0.683769
O	0.330852	0.435806	0.579733
O	0.334369	0.101944	0.581451
O	0.665803	0.101933	0.581456
O	0.669292	0.435820	0.579729
O	0.000067	0.433755	0.581361
O	0.335512	0.564367	0.637855
O	0.328206	0.235285	0.636788
O	0.664622	0.564364	0.637854
O	0.672019	0.235255	0.636799
O	0.000061	0.567002	0.637259
O	0.000108	0.233765	0.637255
O	0.166119	0.071435	0.656810
O	0.167121	0.737960	0.656904
O	0.167126	0.405910	0.658204
O	0.500079	0.068880	0.655266
O	0.500064	0.737943	0.656713
O	0.500091	0.390455	0.649776
O	0.834098	0.071440	0.656810
O	0.833019	0.737955	0.656898

O	0.833033	0.405895	0.658200
O	0.166672	0.600125	0.565149
O	0.166560	0.621553	0.716614
O	0.165896	0.266894	0.563532
O	0.166832	0.286643	0.716832
O	0.500063	0.599592	0.564826
O	0.500079	0.623678	0.717194
O	0.500090	0.267720	0.563875
O	0.500100	0.236365	0.704433
O	0.833463	0.600142	0.565154
O	0.833590	0.621573	0.716615
O	0.834260	0.266888	0.563530
O	0.833383	0.286636	0.716834
O	0.166724	0.065570	0.361725
O	0.166782	0.399363	0.361377
O	0.166804	0.733207	0.361833
O	0.500049	0.065639	0.361732
O	0.500056	0.399404	0.361401
O	0.500038	0.733115	0.361794
O	0.833375	0.065573	0.361727
O	0.833325	0.399370	0.361380
O	0.833295	0.733210	0.361832
O	0.166629	0.063993	0.507821
O	0.166351	0.396833	0.507033
O	0.166683	0.730087	0.508204
O	0.500070	0.063700	0.507599
O	0.500056	0.396897	0.507248
O	0.500066	0.730131	0.508214
O	0.833499	0.063995	0.507824
O	0.833769	0.396838	0.507032
O	0.833459	0.730094	0.508207
O	0.166771	0.266771	0.417354
O	0.166721	0.600609	0.417674
O	0.166798	0.933119	0.417814
O	0.500047	0.266761	0.417378
O	0.500046	0.600460	0.417675
O	0.500048	0.933216	0.417817
O	0.833339	0.266765	0.417353
O	0.833371	0.600624	0.417678
O	0.833324	0.933115	0.417818
O	0.166871	0.934034	0.564774
O	0.500072	0.933546	0.564602
O	0.833294	0.934043	0.564774

O	0.167249	0.954738	0.716462
O	0.500101	0.955706	0.716417
O	0.832939	0.954772	0.716460
O	0.000036	0.120472	0.282976
O	0.000060	0.455560	0.282841
O	0.000027	0.788111	0.282865
O	0.333357	0.120507	0.282936
O	0.333393	0.455656	0.282807
O	0.333351	0.788123	0.282851
O	0.666729	0.120510	0.282937
O	0.666748	0.455664	0.282808
O	0.666705	0.788128	0.282846
O	0.000055	0.100678	0.434391
O	0.000043	0.434972	0.433997
O	0.000055	0.767864	0.434832
O	0.333280	0.100856	0.434364
O	0.333396	0.434759	0.434099
O	0.333535	0.767859	0.434850
O	0.666835	0.100857	0.434364
O	0.666709	0.434758	0.434102
O	0.666585	0.767865	0.434848
O	0.000089	0.101126	0.581560
O	0.000081	0.767591	0.581574
O	0.333409	0.767372	0.581545
O	0.666725	0.767369	0.581545
O	0.000052	0.238836	0.342067
O	0.000052	0.573001	0.342298
O	0.000043	0.905559	0.342257
O	0.333335	0.238847	0.342052
O	0.333340	0.572947	0.342329
O	0.333395	0.905590	0.342255
O	0.666761	0.238851	0.342052
O	0.666755	0.572953	0.342328
O	0.666691	0.905586	0.342255
O	0.000063	0.231363	0.490587
O	0.000075	0.564466	0.491169
O	0.000070	0.897959	0.491552
O	0.333393	0.231219	0.490723
O	0.333268	0.564548	0.491062
O	0.333169	0.898199	0.491610
O	0.666731	0.231227	0.490722
O	0.666828	0.564548	0.491063
O	0.666951	0.898191	0.491616

O	0.000072	0.899198	0.637792
O	0.333087	0.899439	0.637742
O	0.667060	0.899437	0.637742
O	0.500110	0.384857	0.728231

Structure 9: B-type configuration of O^- on SnO_2(101) surface.

```
_cell_length_a   16.97242900
_cell_length_b   14.11702300
_cell_length_c   33.67701300
_cell_angle_alpha   90.00000000
_cell_angle_beta   90.00000000
_cell_angle_gamma   90.00000000
loop_
 _atom_site_type_symbol
 _atom_site_fract_x
 _atom_site_fract_y
 _atom_site_fract_z
  Sn  0.385582  0.177694  0.688585
  Sn  0.377293  0.501207  0.693901
  Sn  0.051420  0.514439  0.688917
  Sn  0.158059  0.497206  0.612900
  Sn  0.321781  0.332019  0.610897
  Sn  0.229733  0.334448  0.694824
  Sn  0.550980  0.319329  0.689156
  Sn  0.212814  0.659082  0.688156
  Sn  0.159679  0.167341  0.614087
  Sn  0.157536  0.831741  0.612297
  Sn  0.491201  0.163644  0.612419
  Sn  0.487524  0.495670  0.614260
  Sn  0.490830  0.829576  0.613237
  Sn  0.824347  0.164670  0.612789
  Sn  0.824164  0.497235  0.612914
  Sn  0.824329  0.831137  0.613033
  Sn  0.029761  0.165786  0.457325
  Sn  0.029694  0.499107  0.457347
  Sn  0.029716  0.832852  0.457463
  Sn  0.362993  0.165556  0.457277
  Sn  0.362970  0.499055  0.457272
  Sn  0.363033  0.832039  0.457424
  Sn  0.696414  0.165786  0.457277
  Sn  0.696116  0.499349  0.457700
  Sn  0.696319  0.832338  0.457524
  Sn  0.239073  0.153103  0.302751
  Sn  0.239051  0.486574  0.302710
  Sn  0.239040  0.819870  0.302756
  Sn  0.572443  0.153199  0.302788
  Sn  0.572446  0.486696  0.302805
```

Sn	0.572423	0.819807	0.302849
Sn	0.905744	0.153215	0.302768
Sn	0.905747	0.486751	0.302903
Sn	0.905695	0.819899	0.302858
Sn	0.056433	0.187497	0.690590
Sn	0.049400	0.848378	0.688978
Sn	0.382908	0.847055	0.689219
Sn	0.717972	0.180510	0.689094
Sn	0.716912	0.512960	0.689307
Sn	0.716684	0.845904	0.689230
Sn	0.259465	0.166663	0.534587
Sn	0.259389	0.501317	0.534596
Sn	0.260235	0.833292	0.535038
Sn	0.593362	0.167396	0.534706
Sn	0.592555	0.500943	0.535655
Sn	0.593077	0.833823	0.535096
Sn	0.926633	0.167994	0.534834
Sn	0.926576	0.500916	0.534913
Sn	0.926888	0.835099	0.535129
Sn	0.132199	0.168961	0.379299
Sn	0.132091	0.502498	0.379275
Sn	0.132098	0.835773	0.379329
Sn	0.465564	0.169038	0.379320
Sn	0.465543	0.502541	0.379337
Sn	0.465528	0.835566	0.379420
Sn	0.798887	0.169176	0.379306
Sn	0.798812	0.502641	0.379549
Sn	0.798835	0.835756	0.379478
Sn	0.217135	0.987126	0.689254
Sn	0.550692	0.985994	0.689187
Sn	0.546152	0.647650	0.689711
Sn	0.884358	0.987808	0.689146
Sn	0.885466	0.320526	0.689308
Sn	0.883640	0.652797	0.689140
Sn	0.093268	0.000106	0.535305
Sn	0.093708	0.332672	0.534923
Sn	0.093102	0.665958	0.534869
Sn	0.426716	0.998504	0.534790
Sn	0.427089	0.332864	0.534554
Sn	0.426483	0.664807	0.535257
Sn	0.759943	0.999153	0.534947
Sn	0.759724	0.332661	0.534857
Sn	0.759742	0.665887	0.535307

Sn	0.298881	0.330813	0.379254
Sn	0.298794	0.664153	0.379310
Sn	0.298884	0.997275	0.379353
Sn	0.632283	0.330963	0.379344
Sn	0.632176	0.664134	0.379524
Sn	0.632239	0.997350	0.379353
Sn	0.965531	0.330852	0.379342
Sn	0.965433	0.664317	0.379508
Sn	0.965487	0.997492	0.379358
Sn	0.324353	0.002332	0.612641
Sn	0.325332	0.667261	0.613914
Sn	0.657636	0.002335	0.612892
Sn	0.657186	0.336056	0.612766
Sn	0.656866	0.668870	0.613606
Sn	0.991829	0.005465	0.613541
Sn	0.991231	0.335902	0.612800
Sn	0.990658	0.669575	0.612739
Sn	0.196272	0.000394	0.457363
Sn	0.196227	0.333979	0.457148
Sn	0.196149	0.667684	0.457265
Sn	0.529769	0.000592	0.457317
Sn	0.529821	0.334271	0.457230
Sn	0.529641	0.667239	0.457594
Sn	0.863016	0.000997	0.457402
Sn	0.862947	0.334310	0.457340
Sn	0.863001	0.667771	0.457648
Sn	0.072393	0.013267	0.302756
Sn	0.072370	0.346690	0.302738
Sn	0.072313	0.680031	0.302817
Sn	0.405793	0.013207	0.302787
Sn	0.405766	0.346735	0.302736
Sn	0.405726	0.680029	0.302761
Sn	0.739112	0.013259	0.302812
Sn	0.739117	0.346885	0.302822
Sn	0.739071	0.679975	0.302922
O	0.130516	0.277243	0.714976
O	0.459493	0.265461	0.714705
O	0.120772	0.608069	0.714570
O	0.455263	0.594336	0.716522
O	0.227744	0.269459	0.636348
O	0.226371	0.603340	0.633788
O	0.317961	0.400701	0.661214
O	0.313114	0.736299	0.667780

O	0.150754	0.441182	0.669042
O	0.483296	0.431893	0.669651
O	0.294185	0.225889	0.714839
O	0.291859	0.583642	0.715378
O	0.394717	0.229287	0.633213
O	0.394008	0.564817	0.637358
O	0.227360	0.935130	0.633988
O	0.562057	0.266479	0.634073
O	0.560186	0.599866	0.634378
O	0.561530	0.933394	0.634248
O	0.894435	0.268658	0.634147
O	0.893618	0.602190	0.633830
O	0.894546	0.935615	0.634065
O	0.099438	0.269237	0.480428
O	0.099213	0.603099	0.480295
O	0.099283	0.936545	0.480617
O	0.433004	0.269392	0.480213
O	0.432794	0.602498	0.480560
O	0.433053	0.935569	0.480411
O	0.766000	0.269639	0.480402
O	0.766058	0.603026	0.480738
O	0.766143	0.936195	0.480507
O	0.306890	0.268996	0.322979
O	0.306938	0.602318	0.323008
O	0.306928	0.935487	0.323066
O	0.640279	0.269111	0.323053
O	0.640296	0.602333	0.323189
O	0.640285	0.935533	0.323103
O	0.973476	0.269029	0.323027
O	0.973510	0.602463	0.323147
O	0.973524	0.935606	0.323084
O	0.123901	0.935725	0.714973
O	0.457202	0.935790	0.714886
O	0.792441	0.269256	0.714924
O	0.790086	0.603036	0.714986
O	0.790422	0.936743	0.714409
O	0.329855	0.268841	0.557002
O	0.329746	0.602743	0.558843
O	0.329549	0.936657	0.558232
O	0.662915	0.270245	0.558252
O	0.662646	0.603658	0.558846
O	0.663008	0.936755	0.558397
O	0.996380	0.270514	0.558249

O	0.995602	0.604448	0.558088
O	0.996312	0.938463	0.558727
O	0.202073	0.269298	0.402732
O	0.201947	0.602806	0.402829
O	0.202098	0.935752	0.402904
O	0.535576	0.269320	0.402841
O	0.535456	0.602603	0.403066
O	0.535541	0.935753	0.402938
O	0.868728	0.269446	0.402888
O	0.868650	0.602962	0.403110
O	0.868766	0.936006	0.402999
O	0.088684	0.066291	0.589310
O	0.086921	0.398106	0.588757
O	0.086836	0.731972	0.588675
O	0.421128	0.063447	0.588482
O	0.419796	0.396350	0.588120
O	0.420820	0.730063	0.589221
O	0.754172	0.064331	0.588934
O	0.753818	0.397687	0.588874
O	0.753839	0.730786	0.589332
O	0.293124	0.062771	0.433408
O	0.293090	0.396504	0.433279
O	0.293082	0.729842	0.433400
O	0.626650	0.062960	0.433454
O	0.626653	0.396608	0.433486
O	0.626545	0.729639	0.433685
O	0.959864	0.063216	0.433464
O	0.959816	0.396509	0.433451
O	0.959837	0.730086	0.433641
O	0.165739	0.063385	0.276878
O	0.165706	0.396863	0.276869
O	0.165656	0.730185	0.276932
O	0.499138	0.063400	0.276934
O	0.499137	0.396975	0.276935
O	0.499141	0.730114	0.276949
O	0.832430	0.063427	0.276933
O	0.832451	0.397097	0.276995
O	0.832409	0.730104	0.277045
O	0.316555	0.062538	0.668259
O	0.649480	0.064439	0.668610
O	0.650496	0.396961	0.668787
O	0.647858	0.730143	0.669321
O	0.982686	0.066364	0.669318

O	0.983230	0.398373	0.668450
O	0.981827	0.731884	0.668680
O	0.189496	0.064239	0.511228
O	0.189276	0.398063	0.510652
O	0.189581	0.730964	0.511185
O	0.523266	0.063790	0.511219
O	0.523475	0.397585	0.511146
O	0.523082	0.730255	0.511611
O	0.856443	0.064385	0.511370
O	0.856413	0.397500	0.511345
O	0.856474	0.731136	0.511635
O	0.062024	0.065019	0.357736
O	0.062036	0.398385	0.357726
O	0.062027	0.731722	0.357821
O	0.395450	0.064900	0.357769
O	0.395462	0.398414	0.357729
O	0.395447	0.731603	0.357772
O	0.728791	0.065011	0.357802
O	0.728849	0.398509	0.357849
O	0.728792	0.731641	0.357936
O	0.149506	0.102460	0.669702
O	0.147325	0.771293	0.668069
O	0.483381	0.099809	0.668101
O	0.481008	0.766501	0.669409
O	0.815878	0.102721	0.668678
O	0.816475	0.435661	0.668877
O	0.815573	0.768513	0.668875
O	0.023147	0.102923	0.511380
O	0.023023	0.435785	0.511295
O	0.023133	0.769762	0.511436
O	0.356256	0.101538	0.511030
O	0.356136	0.435682	0.510961
O	0.356665	0.768484	0.511392
O	0.689958	0.102470	0.511279
O	0.689472	0.436065	0.511666
O	0.689764	0.769140	0.511574
O	0.228773	0.101389	0.357746
O	0.228679	0.434868	0.357675
O	0.228719	0.768230	0.357742
O	0.562153	0.101466	0.357787
O	0.562110	0.434934	0.357783
O	0.562144	0.768151	0.357866
O	0.895432	0.101565	0.357785

O	0.895378	0.435015	0.357891
O	0.895446	0.768372	0.357898
O	0.254583	0.103081	0.588902
O	0.251740	0.434000	0.587025
O	0.254221	0.768451	0.588863
O	0.587487	0.101545	0.588551
O	0.585915	0.435209	0.589420
O	0.587242	0.768533	0.589137
O	0.920744	0.103335	0.588851
O	0.920536	0.435594	0.588848
O	0.920623	0.769587	0.588973
O	0.126537	0.103267	0.433401
O	0.126322	0.436702	0.433263
O	0.126451	0.770424	0.433479
O	0.459899	0.103227	0.433393
O	0.459889	0.436803	0.433357
O	0.459907	0.769829	0.433540
O	0.793266	0.103544	0.433448
O	0.793035	0.437010	0.433648
O	0.793219	0.770260	0.433659
O	0.332445	0.102893	0.276933
O	0.332409	0.436403	0.276847
O	0.332414	0.769753	0.276898
O	0.665791	0.102973	0.276964
O	0.665794	0.436591	0.276941
O	0.665800	0.769692	0.277027
O	0.999073	0.102996	0.276922
O	0.999028	0.436565	0.276965
O	0.999022	0.769791	0.276969
O	0.060754	0.232018	0.634305
O	0.060226	0.565823	0.633524
O	0.060806	0.899634	0.633887
O	0.394981	0.898556	0.634079
O	0.727893	0.232032	0.633925
O	0.727512	0.564685	0.634253
O	0.727414	0.898007	0.634093
O	0.265799	0.229890	0.480105
O	0.266173	0.564338	0.480295
O	0.266254	0.896835	0.480539
O	0.599577	0.230396	0.480289
O	0.599302	0.563830	0.480884
O	0.599456	0.896891	0.480565
O	0.932664	0.230497	0.480337

O	0.932695	0.563775	0.480436
O	0.932812	0.897564	0.480578
O	0.140168	0.230795	0.322984
O	0.140168	0.564325	0.323003
O	0.140231	0.897592	0.323040
O	0.473586	0.230867	0.323015
O	0.473604	0.564372	0.323072
O	0.473666	0.897483	0.323098
O	0.806869	0.230934	0.323026
O	0.806874	0.564373	0.323221
O	0.806933	0.897580	0.323141
O	0.290198	0.898046	0.715430
O	0.625344	0.230511	0.715114
O	0.623327	0.561901	0.715233
O	0.624201	0.897543	0.715307
O	0.960725	0.233708	0.715639
O	0.956542	0.562403	0.714425
O	0.956436	0.897085	0.715243
O	0.163840	0.230802	0.558597
O	0.162284	0.563613	0.558111
O	0.163155	0.896821	0.558091
O	0.496575	0.229346	0.557942
O	0.495365	0.562250	0.559041
O	0.496183	0.895577	0.558502
O	0.829501	0.229968	0.558259
O	0.829371	0.562744	0.558388
O	0.829517	0.896523	0.558429
O	0.035472	0.230574	0.402868
O	0.035357	0.564054	0.402910
O	0.035393	0.897367	0.402946
O	0.368788	0.230456	0.402826
O	0.368777	0.564051	0.402887
O	0.368806	0.897023	0.402960
O	0.702164	0.230613	0.402868
O	0.702036	0.564039	0.403154
O	0.702052	0.897123	0.403021
O	0.297937	0.423835	0.732919

Structure 10: S-type configuration of O on $SnO_2(110)$ surface.

```
_cell_length_a   9.42435700
_cell_length_b   19.96585700
_cell_length_c   37.96585700
_cell_angle_alpha   90.00000000
_cell_angle_beta   90.00000000
_cell_angle_gamma   90.00000000
loop_
 _atom_site_type_symbol
 _atom_site_fract_x
 _atom_site_fract_y
 _atom_site_fract_z
  Sn  0.499218  0.666731  0.723356
  Sn  0.832857  0.333765  0.724332
  Sn  0.166929  0.332852  0.724814
  Sn  0.500927  0.500100  0.630053
  Sn  0.327277  0.504758  0.715117
  Sn  0.673560  0.506184  0.718204
  Sn  0.165766  0.668267  0.724515
  Sn  0.166402  0.999536  0.724565
  Sn  0.499198  0.327925  0.725339
  Sn  0.500002  -0.000349  0.724594
  Sn  0.834521  0.667932  0.724546
  Sn  0.833426  -0.000341  0.724565
  Sn  0.166765  0.166370  0.628866
  Sn  0.166616  0.499651  0.628446
  Sn  0.166549  0.833232  0.628918
  Sn  0.499997  0.166398  0.628938
  Sn  0.499993  0.833231  0.628816
  Sn  0.833330  0.166397  0.628871
  Sn  0.832895  0.499579  0.629884
  Sn  0.833391  0.833193  0.628895
  Sn  0.166664  0.333204  0.370314
  Sn  0.166660  0.666536  0.370237
  Sn  0.166632  0.999894  0.370202
  Sn  0.500002  0.333191  0.370299
  Sn  0.499998  0.666548  0.370232
  Sn  0.500000  0.999886  0.370217
  Sn  0.833323  0.333202  0.370312
  Sn  0.833332  0.666539  0.370237
  Sn  0.833352  0.999893  0.370202
  Sn  0.166653  0.166633  0.274667
```

Sn 0.166663 0.499915 0.274762
Sn 0.166649 0.833216 0.274648
Sn 0.499994 0.166632 0.274681
Sn 0.499999 0.499914 0.274760
Sn 0.499988 0.833216 0.274664
Sn 0.833333 0.166631 0.274668
Sn 0.833318 0.499915 0.274760
Sn 0.833330 0.833217 0.274648
Sn -0.000183 0.166497 0.715313
Sn 0.001204 0.498386 0.715256
Sn -0.000179 0.833474 0.715632
Sn 0.332280 0.164718 0.715407
Sn 0.333580 0.833528 0.715447
Sn 0.667678 0.165397 0.715406
Sn 0.666330 0.833688 0.715419
Sn 0.000249 0.332903 0.633498
Sn 0.000072 0.666667 0.633416
Sn -0.000029 0.999824 0.633401
Sn 0.333644 0.333387 0.633627
Sn 0.333098 0.666961 0.633143
Sn 0.333329 0.999791 0.633412
Sn 0.666570 0.333238 0.633704
Sn 0.666897 0.666779 0.633256
Sn 0.666672 0.999781 0.633410
Sn -0.000004 0.166611 0.365791
Sn -0.000013 0.499859 0.365901
Sn -0.000010 0.833170 0.365772
Sn 0.333322 0.166579 0.365797
Sn 0.333350 0.499868 0.365906
Sn 0.333308 0.833189 0.365780
Sn 0.666671 0.166580 0.365797
Sn 0.666660 0.499868 0.365906
Sn 0.666677 0.833189 0.365781
Sn -0.000010 0.333284 0.283703
Sn -0.000012 0.666561 0.283648
Sn -0.000007 0.999926 0.283610
Sn 0.333325 0.333261 0.283704
Sn 0.333332 0.666573 0.283651
Sn 0.333338 0.999917 0.283621
Sn 0.666670 0.333262 0.283701
Sn 0.666647 0.666573 0.283650
Sn 0.666643 0.999916 0.283622
Sn 0.166809 0.333104 0.544982

Sn	0.166600	0.666382	0.544819
Sn	0.166648	0.999782	0.544821
Sn	0.500133	0.333089	0.545001
Sn	0.500111	0.666429	0.544758
Sn	0.499998	0.999768	0.544819
Sn	0.833285	0.333053	0.544983
Sn	0.833390	0.666407	0.544823
Sn	0.833349	0.999782	0.544819
Sn	0.166671	0.166494	0.454350
Sn	0.166709	0.499782	0.454503
Sn	0.166632	0.833117	0.454341
Sn	0.500008	0.166483	0.454344
Sn	0.500104	0.499789	0.454574
Sn	0.499992	0.833121	0.454315
Sn	0.833341	0.166490	0.454351
Sn	0.833247	0.499783	0.454555
Sn	0.833359	0.833123	0.454337
Sn	0.000038	0.166500	0.541971
Sn	-0.000182	0.499743	0.542228
Sn	0.000003	0.833091	0.541986
Sn	0.333393	0.166474	0.541980
Sn	0.333712	0.499776	0.542290
Sn	0.333309	0.833105	0.541974
Sn	0.666650	0.166467	0.541984
Sn	0.666726	0.499764	0.542474
Sn	0.666691	0.833120	0.541963
Sn	0.000011	0.333133	0.457282
Sn	0.000000	0.666459	0.457162
Sn	-0.000002	0.999822	0.457150
Sn	0.333365	0.333136	0.457286
Sn	0.333331	0.666463	0.457168
Sn	0.333318	0.999808	0.457142
Sn	0.666655	0.333121	0.457291
Sn	0.666688	0.666479	0.457168
Sn	0.666679	0.999808	0.457140
O	0.495573	0.563397	0.716203
O	0.843119	0.563789	0.722932
O	0.161426	0.563591	0.722324
O	0.484369	0.435851	0.725122
O	0.835940	0.438534	0.721518
O	0.168901	0.439068	0.723772
O	0.334045	0.664418	0.754236
O	0.665244	0.661496	0.753971

O	0.999482	0.333087	0.754774
O	0.331200	0.497906	0.663677
O	0.667370	0.498955	0.665550
O	0.334935	0.334651	0.686876
O	0.165399	0.104111	0.722997
O	0.167301	0.772416	0.723367
O	0.500357	0.103798	0.722871
O	0.500328	0.771651	0.723238
O	0.834005	0.104316	0.723079
O	0.832014	0.772192	0.723505
O	0.167466	0.267582	0.631782
O	0.166941	0.601142	0.631607
O	0.166621	0.934337	0.631539
O	0.499817	0.267687	0.631823
O	0.499900	0.601348	0.631924
O	0.499989	0.934351	0.631501
O	0.833105	0.267606	0.631770
O	0.833341	0.601063	0.631976
O	0.833360	0.934340	0.631517
O	0.166644	0.101147	0.367735
O	0.166678	0.434423	0.367834
O	0.166733	0.767758	0.367762
O	0.499995	0.101145	0.367756
O	0.500009	0.434408	0.367863
O	0.499991	0.767774	0.367762
O	0.833351	0.101149	0.367735
O	0.833310	0.434418	0.367823
O	0.833256	0.767758	0.367759
O	0.166638	0.271572	0.276326
O	0.166707	0.604834	0.276393
O	0.166687	0.938191	0.276313
O	0.499992	0.271555	0.276378
O	0.499991	0.604839	0.276383
O	0.499990	0.938187	0.276318
O	0.833355	0.271571	0.276326
O	0.833280	0.604835	0.276391
O	0.833301	0.938190	0.276313
O	0.166844	0.227590	0.722121
O	0.166702	0.895192	0.722503
O	0.499365	0.225791	0.722202
O	0.499821	0.895075	0.722726
O	0.833551	0.227957	0.722006
O	0.833264	0.895151	0.722507

O 0.166713 0.065145 0.631302
O 0.166680 0.398270 0.631228
O 0.166425 0.732184 0.630940
O 0.500019 0.065171 0.631391
O 0.500325 0.398724 0.631671
O 0.500096 0.732120 0.631091
O 0.833237 0.065167 0.631317
O 0.833462 0.398294 0.631708
O 0.833452 0.732083 0.631052
O 0.166746 0.231999 0.367813
O 0.166660 0.565310 0.367839
O 0.166637 0.898638 0.367725
O 0.499996 0.231985 0.367794
O 0.500005 0.565325 0.367857
O 0.499991 0.898611 0.367792
O 0.833246 0.231998 0.367809
O 0.833330 0.565315 0.367827
O 0.833344 0.898636 0.367727
O 0.166709 0.061656 0.276340
O 0.166694 0.395007 0.276426
O 0.166655 0.728279 0.276320
O 0.499993 0.061654 0.276336
O 0.499993 0.394999 0.276435
O 0.499982 0.728290 0.276359
O 0.833277 0.061656 0.276338
O 0.833298 0.395007 0.276427
O 0.833322 0.728280 0.276322
O 0.000267 0.670316 0.755114
O -0.000106 0.998981 0.754930
O 0.332197 0.327588 0.755434
O 0.333139 0.999546 0.754878
O 0.667064 0.327506 0.754803
O 0.666779 0.999232 0.754896
O 0.000042 0.165798 0.663508
O 0.002023 0.499683 0.663535
O -0.000130 0.832821 0.663802
O 0.333279 0.165801 0.663632
O 0.333473 0.833026 0.663663
O 0.666704 0.165919 0.663622
O 0.666550 0.833047 0.663648
O -0.000005 0.333146 0.403505
O -0.000000 0.666542 0.403417
O -0.000003 0.999879 0.403403

O 0.333364 0.333161 0.403506
O 0.333351 0.666547 0.403426
O 0.333284 0.999885 0.403389
O 0.666633 0.333138 0.403505
O 0.666644 0.666554 0.403425
O 0.666705 0.999885 0.403388
O -0.000004 0.166727 0.312823
O -0.000007 0.499887 0.312919
O -0.000004 0.833131 0.312804
O 0.333234 0.166711 0.312834
O 0.333321 0.499914 0.312929
O 0.333251 0.833164 0.312818
O 0.666753 0.166709 0.312834
O 0.666673 0.499915 0.312927
O 0.666740 0.833165 0.312817
O 0.001187 0.333291 0.686429
O 0.000184 0.669034 0.686355
O -0.000093 0.999976 0.686356
O 0.331159 0.669356 0.685964
O 0.333227 0.999968 0.686387
O 0.664598 0.333564 0.686720
O 0.668177 0.668603 0.686099
O 0.666772 0.999847 0.686387
O 0.000005 0.166642 0.420371
O -0.000057 0.499734 0.420513
O 0.000000 0.833093 0.420351
O 0.333288 0.166623 0.420371
O 0.333425 0.499810 0.420519
O 0.333200 0.833071 0.420355
O 0.666711 0.166617 0.420371
O 0.666671 0.499807 0.420516
O 0.666770 0.833075 0.420353
O -0.000005 0.333148 0.335543
O -0.000003 0.666617 0.335485
O -0.000006 0.999914 0.335449
O 0.333261 0.333184 0.335540
O 0.333294 0.666573 0.335488
O 0.333360 0.999907 0.335454
O 0.666727 0.333186 0.335536
O 0.666693 0.666575 0.335486
O 0.666629 0.999909 0.335454
O -0.000008 0.166542 0.244338
O -0.000004 0.499947 0.244429

O	-0.000011	0.833227	0.244313
O	0.333344	0.166655	0.244344
O	0.333323	0.499930	0.244431
O	0.333343	0.833181	0.244325
O	0.666641	0.166656	0.244345
O	0.666658	0.499930	0.244428
O	0.666635	0.833180	0.244324
O	0.166713	0.101836	0.543061
O	0.166807	0.435148	0.543465
O	0.166728	0.768396	0.543038
O	0.500010	0.101798	0.543053
O	0.500362	0.435110	0.543366
O	0.500021	0.768434	0.543061
O	0.833324	0.101827	0.543069
O	0.833095	0.435065	0.543359
O	0.833279	0.768419	0.543044
O	0.166707	0.268493	0.456241
O	0.166650	0.601801	0.456172
O	0.166681	0.935144	0.456090
O	0.499996	0.268464	0.456253
O	0.500031	0.601842	0.456207
O	0.499991	0.935141	0.456046
O	0.833323	0.268483	0.456237
O	0.833353	0.601821	0.456168
O	0.833321	0.935148	0.456088
O	0.166829	0.231126	0.543021
O	0.166740	0.564353	0.543293
O	0.166665	0.897739	0.542975
O	0.500027	0.231108	0.543038
O	0.500377	0.564442	0.543213
O	0.499989	0.897738	0.542995
O	0.833269	0.231095	0.543012
O	0.833121	0.564402	0.543200
O	0.833334	0.897747	0.542970
O	0.166705	0.064479	0.456099
O	0.166707	0.397769	0.456257
O	0.166680	0.731104	0.456147
O	0.499996	0.064462	0.456083
O	0.500025	0.397746	0.456299
O	0.499995	0.731140	0.456176
O	0.833298	0.064476	0.456099
O	0.833309	0.397749	0.456259
O	0.833336	0.731116	0.456139

O -0.000017 0.332687 0.578963
O -0.000081 0.666289 0.578834
O 0.000001 0.999744 0.578810
O 0.333579 0.332945 0.578995
O 0.333495 0.666353 0.578738
O 0.333314 0.999657 0.578817
O 0.666748 0.332903 0.579052
O 0.666695 0.666249 0.578798
O 0.666674 0.999674 0.578817
O 0.000015 0.166432 0.488685
O 0.000048 0.499763 0.488894
O -0.000002 0.833070 0.488674
O 0.333372 0.166408 0.488681
O 0.333329 0.499754 0.488916
O 0.333360 0.833086 0.488668
O 0.666657 0.166402 0.488685
O 0.666709 0.499756 0.488958
O 0.666639 0.833097 0.488662
O 0.000054 0.166770 0.595688
O -0.001371 0.499846 0.595917
O -0.000022 0.833440 0.595729
O 0.333500 0.166563 0.595724
O 0.334929 0.500101 0.596028
O 0.333210 0.833365 0.595711
O 0.666559 0.166648 0.595737
O 0.666738 0.500042 0.596594
O 0.666769 0.833356 0.595703
O 0.000046 0.333242 0.510624
O 0.000001 0.666376 0.510489
O 0.000007 0.999814 0.510471
O 0.333418 0.333262 0.510633
O 0.333319 0.666355 0.510486
O 0.333294 0.999796 0.510471
O 0.666689 0.333115 0.510636
O 0.666769 0.666508 0.510484
O 0.666693 0.999791 0.510470
O 0.566243 0.462604 0.757260

Structure 11: S_1-type configuration of O on $SnO_2(100)$ surface.

_cell_length_a 9.42441600
_cell_length_b 14.11538000
_cell_length_c 32.11538000
_cell_angle_alpha 90.00000000
_cell_angle_beta 90.00000000
_cell_angle_gamma 90.00000000
loop_
_atom_site_type_symbol
_atom_site_fract_x
_atom_site_fract_y
_atom_site_fract_z
Sn 0.326401 0.331044 0.685661
Sn 0.334263 0.661145 0.684282
Sn 0.333591 0.332098 0.536456
Sn 0.667261 0.661229 0.684132
Sn 0.666973 0.332066 0.536255
Sn 0.672213 0.329406 0.681925
Sn -0.001356 0.326342 0.683501
Sn 0.167025 0.499272 0.610004
Sn 0.167196 0.166489 0.610309
Sn 0.500865 0.497339 0.610214
Sn 0.499460 0.166649 0.610172
Sn 0.834228 0.499881 0.609261
Sn 0.833664 0.164067 0.609039
Sn 0.166699 0.161079 0.315316
Sn 0.166710 0.494601 0.315313
Sn 0.166715 0.827818 0.315317
Sn 0.500046 0.161100 0.315302
Sn 0.500048 0.494532 0.315283
Sn 0.500042 0.827840 0.315307
Sn 0.833401 0.161086 0.315326
Sn 0.833392 0.494595 0.315319
Sn 0.833392 0.827826 0.315332
Sn 0.166895 0.165547 0.462937
Sn 0.166964 0.498813 0.462971
Sn 0.167033 0.832183 0.462990
Sn 0.500077 0.165520 0.462894
Sn 0.500189 0.498767 0.462935
Sn 0.500047 0.832258 0.462979
Sn 0.833548 0.165525 0.462953
Sn 0.833585 0.498908 0.462967

Sn	0.833477	0.832099	0.462938
Sn	0.167163	0.832313	0.609789
Sn	0.500265	0.832244	0.609751
Sn	0.833622	0.832361	0.609741
Sn	0.000127	0.332363	0.389521
Sn	0.000160	0.665664	0.389562
Sn	0.000125	0.998930	0.389571
Sn	0.333438	0.332329	0.389492
Sn	0.333481	0.665640	0.389537
Sn	0.333443	0.998988	0.389540
Sn	0.666799	0.332332	0.389516
Sn	0.666742	0.665649	0.389548
Sn	0.666765	0.998983	0.389553
Sn	0.000395	0.332132	0.536403
Sn	0.000607	0.665785	0.536295
Sn	0.000431	0.998732	0.536332
Sn	0.333973	0.665187	0.536506
Sn	0.333537	0.999243	0.536523
Sn	0.666525	0.665506	0.536344
Sn	0.666596	0.998827	0.536332
Sn	0.000678	0.661253	0.684008
Sn	0.000377	0.994441	0.684021
Sn	0.333604	0.994597	0.684049
Sn	0.667042	0.994454	0.683983
O	0.333616	0.432608	0.582247
O	0.333462	0.100814	0.581974
O	0.667360	0.433079	0.581686
O	0.665606	0.099737	0.581540
O	0.335235	0.564266	0.638252
O	0.333419	0.234957	0.638443
O	0.666731	0.564754	0.637931
O	0.668660	0.232060	0.637004
O	-0.001964	0.233162	0.636463
O	0.163587	0.407592	0.658236
O	0.166794	0.071885	0.657179
O	0.500513	0.738266	0.656945
O	0.500110	0.397466	0.508178
O	0.502768	0.399341	0.657088
O	0.500291	0.072921	0.657521
O	0.835606	0.405661	0.656105
O	0.833746	0.071674	0.656980
O	0.167150	0.620134	0.716241
O	0.167152	0.267011	0.564741

O	0.159720	0.277374	0.713946
O	0.500419	0.599535	0.564871
O	0.500979	0.621433	0.716639
O	0.500299	0.267196	0.564737
O	0.443652	0.352450	0.736325
O	0.834183	0.619848	0.716122
O	0.833402	0.266437	0.564237
O	0.166736	0.066467	0.361975
O	0.166766	0.399934	0.361962
O	0.166825	0.733094	0.361949
O	0.500103	0.066505	0.361965
O	0.500107	0.399883	0.361943
O	0.500096	0.733066	0.361920
O	0.833465	0.066476	0.361992
O	0.833426	0.399947	0.361982
O	0.833395	0.733120	0.361980
O	0.167096	0.064026	0.508029
O	0.167208	0.397459	0.508181
O	0.167381	0.730461	0.508043
O	0.499952	0.064115	0.508041
O	0.500084	0.730501	0.508078
O	0.833488	0.064137	0.508115
O	0.833650	0.397633	0.508146
O	0.833485	0.730565	0.507995
O	0.167701	0.738395	0.657082
O	0.833503	0.738635	0.657119
O	0.166813	0.266628	0.417618
O	0.166892	0.599898	0.417677
O	0.166887	0.933303	0.417693
O	0.500113	0.266665	0.417618
O	0.500120	0.599807	0.417630
O	0.500118	0.933375	0.417686
O	0.833499	0.266628	0.417644
O	0.833450	0.599941	0.417690
O	0.833399	0.933246	0.417696
O	0.167390	0.600176	0.564764
O	0.167254	0.933486	0.564592
O	0.499974	0.933534	0.564579
O	0.833495	0.600557	0.564470
O	0.833384	0.933683	0.564615
O	0.167056	0.952950	0.716115
O	0.500375	0.952952	0.716052
O	0.832160	0.289542	0.716233

O	0.833617	0.953360	0.716193
O	0.000027	0.119561	0.283229
O	0.000038	0.453493	0.283132
O	0.000024	0.786647	0.283129
O	0.333345	0.119732	0.283169
O	0.333306	0.453342	0.283114
O	0.333357	0.786760	0.283097
O	0.666753	0.119760	0.283172
O	0.666782	0.453357	0.283117
O	0.666750	0.786770	0.283114
O	0.000163	0.100066	0.434789
O	0.000230	0.433457	0.434812
O	0.000283	0.766833	0.434755
O	0.333450	0.100271	0.434683
O	0.333487	0.433382	0.434755
O	0.333565	0.766880	0.434709
O	0.666846	0.100134	0.434753
O	0.666916	0.433363	0.434795
O	0.666661	0.766832	0.434745
O	0.001410	0.099589	0.581489
O	0.000924	0.433601	0.581560
O	0.000382	0.766753	0.581561
O	0.333673	0.766586	0.581613
O	0.666998	0.766694	0.581548
O	0.000071	0.239116	0.341946
O	0.000061	0.572323	0.342080
O	0.000099	0.905462	0.342105
O	0.333336	0.238964	0.341995
O	0.333387	0.572281	0.342047
O	0.333407	0.905509	0.342087
O	0.666835	0.238955	0.342017
O	0.666777	0.572270	0.342061
O	0.666739	0.905505	0.342103
O	0.000351	0.230821	0.491213
O	0.000391	0.564029	0.491329
O	0.000207	0.897167	0.491309
O	0.333535	0.230769	0.491261
O	0.333632	0.563916	0.491292
O	0.333520	0.897461	0.491298
O	0.666704	0.230792	0.491145
O	0.666807	0.564015	0.491251
O	0.666853	0.897336	0.491255
O	0.000196	0.567343	0.637061

O	0.000379	0.899457	0.637424
O	0.333806	0.899842	0.637371
O	0.666838	0.899721	0.637342
O	0.516072	0.265853	0.714997

Structure 12: S-type configuration of O on SnO_2(101) surface.

```
_cell_length_a   16.97242900
_cell_length_b   14.11702300
_cell_length_c   33.67701300
_cell_angle_alpha   90.00000000
_cell_angle_beta   90.00000000
_cell_angle_gamma   90.00000000
loop_
 _atom_site_type_symbol
 _atom_site_fract_x
 _atom_site_fract_y
 _atom_site_fract_z
  Sn  0.379582  0.501394  0.691336
  Sn  0.383939  0.847643  0.689416
  Sn  0.490664  0.498686  0.613857
  Sn  0.550062  0.320452  0.689255
  Sn  0.216070  0.653966  0.689159
  Sn  0.557582  0.661974  0.689710
  Sn  0.156950  0.163437  0.612488
  Sn  0.157268  0.497310  0.612900
  Sn  0.157878  0.830594  0.612719
  Sn  0.490874  0.164263  0.612802
  Sn  0.490861  0.831536  0.612688
  Sn  0.824058  0.164363  0.612652
  Sn  0.824221  0.497631  0.612622
  Sn  0.824528  0.831194  0.612614
  Sn  0.029478  0.165732  0.457137
  Sn  0.029438  0.499148  0.457139
  Sn  0.029475  0.832236  0.457100
  Sn  0.362814  0.165954  0.457216
  Sn  0.362734  0.499251  0.457344
  Sn  0.362843  0.832221  0.457337
  Sn  0.696021  0.165869  0.457160
  Sn  0.695951  0.499123  0.457277
  Sn  0.696114  0.832438  0.457149
  Sn  0.238757  0.153283  0.302712
  Sn  0.238741  0.486656  0.302682
  Sn  0.238819  0.819932  0.302686
  Sn  0.572036  0.153308  0.302691
  Sn  0.572032  0.486679  0.302717
  Sn  0.572070  0.819933  0.302711
  Sn  0.905361  0.153268  0.302661
```

Sn	0.905369	0.486591	0.302664
Sn	0.905411	0.819914	0.302617
Sn	0.049522	0.179097	0.689084
Sn	0.050005	0.513194	0.689307
Sn	0.050429	0.846951	0.689115
Sn	0.383356	0.179679	0.689174
Sn	0.716812	0.180544	0.689197
Sn	0.717538	0.513132	0.689080
Sn	0.719143	0.849390	0.689281
Sn	0.259545	0.167267	0.534577
Sn	0.259939	0.500878	0.535064
Sn	0.260016	0.833705	0.534966
Sn	0.592983	0.167846	0.534730
Sn	0.592881	0.500854	0.535063
Sn	0.593178	0.834278	0.534722
Sn	0.926433	0.167561	0.534659
Sn	0.926491	0.500833	0.534670
Sn	0.926495	0.834094	0.534579
Sn	0.131981	0.169103	0.379213
Sn	0.131918	0.502542	0.379185
Sn	0.131928	0.835715	0.379160
Sn	0.465229	0.169211	0.379217
Sn	0.465210	0.502551	0.379278
Sn	0.465237	0.835697	0.379275
Sn	0.798523	0.169147	0.379171
Sn	0.798470	0.502462	0.379198
Sn	0.798536	0.835790	0.379136
Sn	0.217105	0.986888	0.689228
Sn	0.214968	0.316933	0.689073
Sn	0.550738	0.986783	0.689202
Sn	0.884406	0.987675	0.689173
Sn	0.883516	0.319881	0.689169
Sn	0.883952	0.653644	0.689155
Sn	0.093149	0.998730	0.534549
Sn	0.093077	0.332452	0.534702
Sn	0.093216	0.665628	0.534763
Sn	0.426405	0.999308	0.534734
Sn	0.426517	0.333058	0.534980
Sn	0.426440	0.665579	0.535216
Sn	0.759787	0.999286	0.534634
Sn	0.759667	0.332535	0.534740
Sn	0.760048	0.665924	0.534565
Sn	0.298556	0.330951	0.379237

Sn	0.298602	0.664154	0.379246
Sn	0.298689	0.997401	0.379234
Sn	0.631809	0.330892	0.379227
Sn	0.631838	0.664144	0.379269
Sn	0.631856	0.997490	0.379188
Sn	0.965222	0.330836	0.379180
Sn	0.965233	0.664132	0.379092
Sn	0.965252	0.997439	0.379140
Sn	0.324118	0.002559	0.612666
Sn	0.323488	0.334378	0.612325
Sn	0.325101	0.668564	0.613990
Sn	0.657606	0.002984	0.612619
Sn	0.657303	0.336206	0.612817
Sn	0.657960	0.669202	0.612234
Sn	0.990710	0.002097	0.612444
Sn	0.990713	0.335745	0.612713
Sn	0.990902	0.669137	0.612704
Sn	0.196282	0.000573	0.457145
Sn	0.196079	0.334332	0.457213
Sn	0.196176	0.667511	0.457262
Sn	0.529413	0.000894	0.457200
Sn	0.529282	0.334352	0.457266
Sn	0.529394	0.667430	0.457402
Sn	0.862792	0.000863	0.457101
Sn	0.862701	0.334231	0.457161
Sn	0.862813	0.667490	0.457050
Sn	0.072095	0.013358	0.302658
Sn	0.072054	0.346688	0.302682
Sn	0.072137	0.680055	0.302618
Sn	0.405435	0.013314	0.302716
Sn	0.405382	0.346749	0.302719
Sn	0.405446	0.680005	0.302712
Sn	0.738707	0.013352	0.302662
Sn	0.738656	0.346709	0.302679
Sn	0.738711	0.679998	0.302686
O	0.455753	0.270656	0.714508
O	0.459646	0.634736	0.720322
O	0.561501	0.603976	0.635154
O	0.313723	0.393440	0.668797
O	0.649467	0.398085	0.668953
O	0.315424	0.733444	0.668932
O	0.653000	0.734195	0.668022
O	0.482946	0.433694	0.667932

O	0.483896	0.771368	0.668731
O	0.289176	0.565746	0.713944
O	0.625094	0.566012	0.714596
O	0.226648	0.267225	0.633779
O	0.227092	0.601896	0.634060
O	0.227548	0.935047	0.634111
O	0.561172	0.267949	0.634202
O	0.561214	0.935547	0.633996
O	0.893980	0.268480	0.633992
O	0.894065	0.602063	0.633960
O	0.894354	0.935348	0.634064
O	0.099297	0.269499	0.480453
O	0.099465	0.602602	0.480516
O	0.099473	0.935758	0.480385
O	0.432622	0.269687	0.480674
O	0.432865	0.602357	0.480948
O	0.432788	0.935901	0.480579
O	0.765880	0.269521	0.480485
O	0.766109	0.602702	0.480433
O	0.766079	0.936030	0.480442
O	0.306599	0.269121	0.323138
O	0.306709	0.602316	0.323159
O	0.306719	0.935608	0.323150
O	0.639841	0.269097	0.323111
O	0.639934	0.602335	0.323152
O	0.639935	0.935658	0.323108
O	0.973223	0.269058	0.323088
O	0.973336	0.602368	0.323023
O	0.973312	0.935622	0.323061
O	0.122015	0.268440	0.715261
O	0.122236	0.603904	0.714616
O	0.123515	0.936817	0.714742
O	0.457346	0.937367	0.715040
O	0.789729	0.270598	0.714802
O	0.790279	0.603747	0.714552
O	0.791176	0.938850	0.715361
O	0.329401	0.270056	0.558168
O	0.330542	0.602203	0.559634
O	0.329686	0.936828	0.558404
O	0.662612	0.270677	0.558395
O	0.663040	0.603823	0.558210
O	0.662998	0.937048	0.558356
O	0.996141	0.270269	0.558337

O	0.996234	0.603580	0.558355
O	0.996228	0.936724	0.558174
O	0.201946	0.269351	0.402982
O	0.202060	0.602464	0.403075
O	0.202056	0.935691	0.402976
O	0.535158	0.269387	0.403010
O	0.535251	0.602524	0.403130
O	0.535269	0.935835	0.403013
O	0.868554	0.269281	0.402940
O	0.868574	0.602588	0.402889
O	0.868599	0.935844	0.402915
O	0.087163	0.063768	0.588717
O	0.087357	0.397193	0.588953
O	0.087574	0.730549	0.588978
O	0.420719	0.064380	0.588870
O	0.419735	0.398742	0.589163
O	0.421167	0.730148	0.589555
O	0.754010	0.064580	0.588764
O	0.754005	0.397611	0.588907
O	0.754500	0.731071	0.588779
O	0.293203	0.062887	0.433560
O	0.292991	0.396613	0.433590
O	0.293149	0.729584	0.433632
O	0.626330	0.063033	0.433536
O	0.626249	0.396441	0.433598
O	0.626365	0.729589	0.433648
O	0.959725	0.062992	0.433466
O	0.959656	0.396430	0.433503
O	0.959746	0.729612	0.433443
O	0.165650	0.063299	0.277081
O	0.165607	0.396655	0.277100
O	0.165757	0.729892	0.277061
O	0.498933	0.063282	0.277101
O	0.498917	0.396681	0.277117
O	0.499011	0.729863	0.277108
O	0.832235	0.063291	0.277044
O	0.832214	0.396627	0.277080
O	0.832285	0.729849	0.277066
O	0.315772	0.064641	0.668577
O	0.649524	0.064435	0.668888
O	0.982716	0.064231	0.668571
O	0.982414	0.397299	0.668859
O	0.982820	0.731036	0.668756

O	0.189721	0.063721	0.511293
O	0.189910	0.397263	0.511502
O	0.189849	0.730479	0.511493
O	0.523013	0.064230	0.511386
O	0.523038	0.397687	0.511583
O	0.523072	0.730561	0.511636
O	0.856419	0.064094	0.511291
O	0.856425	0.397312	0.511393
O	0.856552	0.730687	0.511251
O	0.062001	0.064830	0.357831
O	0.061969	0.398226	0.357853
O	0.061991	0.731473	0.357795
O	0.395346	0.064866	0.357887
O	0.395290	0.398291	0.357906
O	0.395345	0.731445	0.357900
O	0.728586	0.064861	0.357835
O	0.728556	0.398201	0.357866
O	0.728587	0.731468	0.357859
O	0.149006	0.102212	0.668683
O	0.148711	0.434489	0.668924
O	0.149099	0.769316	0.668806
O	0.482585	0.102338	0.668784
O	0.815995	0.102743	0.668695
O	0.816021	0.435668	0.668650
O	0.817100	0.770542	0.668936
O	0.023139	0.102566	0.511332
O	0.023088	0.435952	0.511355
O	0.023166	0.769166	0.511337
O	0.356347	0.102636	0.511358
O	0.356335	0.436036	0.511532
O	0.356523	0.768736	0.511626
O	0.689685	0.102873	0.511375
O	0.689641	0.436091	0.511511
O	0.689881	0.769413	0.511380
O	0.228715	0.101741	0.357888
O	0.228631	0.435168	0.357849
O	0.228681	0.768387	0.357857
O	0.561961	0.101831	0.357880
O	0.561914	0.435177	0.357893
O	0.561964	0.768424	0.357900
O	0.895284	0.101771	0.357838
O	0.895232	0.435097	0.357833
O	0.895294	0.768462	0.357791

O	0.253884	0.102286	0.588740
O	0.253770	0.435391	0.589209
O	0.254646	0.768716	0.589161
O	0.587368	0.102732	0.588858
O	0.587293	0.436853	0.589398
O	0.587680	0.769641	0.588961
O	0.920722	0.102552	0.588857
O	0.920751	0.435897	0.588889
O	0.921000	0.769435	0.588866
O	0.126451	0.103464	0.433517
O	0.126377	0.437037	0.433526
O	0.126446	0.770123	0.433503
O	0.459724	0.103712	0.433570
O	0.459628	0.437067	0.433639
O	0.459746	0.770095	0.433679
O	0.792969	0.103621	0.433499
O	0.792934	0.436985	0.433555
O	0.793046	0.770263	0.433483
O	0.332302	0.103317	0.277124
O	0.332283	0.436725	0.277091
O	0.332405	0.770081	0.277105
O	0.665574	0.103362	0.277092
O	0.665546	0.436722	0.277085
O	0.665629	0.770052	0.277101
O	0.998939	0.103356	0.277078
O	0.998915	0.436656	0.277071
O	0.999038	0.770114	0.277038
O	0.060553	0.231346	0.634062
O	0.060534	0.564538	0.634085
O	0.060919	0.898268	0.633907
O	0.393817	0.231305	0.633978
O	0.395610	0.566001	0.638076
O	0.394462	0.899139	0.634178
O	0.727409	0.231866	0.634024
O	0.727663	0.565232	0.633934
O	0.728608	0.899922	0.634051
O	0.265875	0.230490	0.480426
O	0.266325	0.564377	0.480886
O	0.266184	0.896935	0.480649
O	0.599234	0.230769	0.480503
O	0.599263	0.563930	0.480738
O	0.599409	0.897309	0.480510
O	0.932669	0.230599	0.480436

O	0.932732	0.563992	0.480453
O	0.932805	0.897244	0.480394
O	0.139942	0.230905	0.323115
O	0.140010	0.564388	0.323107
O	0.140074	0.897582	0.323083
O	0.473221	0.230946	0.323120
O	0.473287	0.564361	0.323184
O	0.473359	0.897583	0.323155
O	0.806516	0.230914	0.323086
O	0.806581	0.564312	0.323097
O	0.806631	0.897599	0.323045
O	0.289156	0.228539	0.714768
O	0.290704	0.898331	0.715117
O	0.622979	0.230121	0.714775
O	0.624110	0.896137	0.714590
O	0.955878	0.228946	0.714642
O	0.956230	0.562950	0.714745
O	0.957055	0.897159	0.714724
O	0.162638	0.229369	0.558222
O	0.162853	0.562728	0.558465
O	0.163010	0.896158	0.558382
O	0.496102	0.230125	0.558506
O	0.496439	0.563716	0.559283
O	0.496187	0.896456	0.558359
O	0.829496	0.229777	0.558303
O	0.829594	0.563126	0.558275
O	0.829768	0.896635	0.558280
O	0.035312	0.230720	0.402942
O	0.035297	0.564162	0.402940
O	0.035264	0.897257	0.402906
O	0.368615	0.230857	0.403019
O	0.368682	0.564248	0.403130
O	0.368627	0.897201	0.403072
O	0.701874	0.230769	0.402958
O	0.701877	0.564091	0.403036
O	0.701863	0.897329	0.402939
O	0.451440	0.536622	0.736326

Structure 13: L-type configuration of O on SnO_2(110) surface.

```
_cell_length_a   9.42435700
_cell_length_b   19.96585700
_cell_length_c   37.96585700
_cell_angle_alpha   90.00000000
_cell_angle_beta   90.00000000
_cell_angle_gamma   90.00000000
loop_
 _atom_site_type_symbol
 _atom_site_fract_x
 _atom_site_fract_y
 _atom_site_fract_z
  Sn  0.842271  0.335659  0.720659
  Sn  0.491306  0.335656  0.720685
  Sn  0.666731  0.490390  0.719903
  Sn  0.332213  0.500626  0.714153
  Sn  0.166772  0.333888  0.724218
  Sn  0.166668  0.665952  0.724663
  Sn  0.166609  0.000168  0.724518
  Sn  0.500458  0.663379  0.725113
  Sn  0.499848  -0.000056  0.724485
  Sn  0.832797  0.663381  0.725086
  Sn  0.833359  -0.000061  0.724466
  Sn  0.166623  0.166389  0.629187
  Sn  0.166636  0.500570  0.627549
  Sn  0.166680  0.833091  0.629230
  Sn  0.499914  0.166327  0.629063
  Sn  0.501698  0.499904  0.630382
  Sn  0.500063  0.833034  0.629200
  Sn  0.833310  0.166309  0.629054
  Sn  0.831610  0.499900  0.630400
  Sn  0.833241  0.833038  0.629194
  Sn  0.166666  0.333179  0.370033
  Sn  0.166655  0.666520  0.370334
  Sn  0.166637  0.999852  0.370203
  Sn  0.499981  0.333166  0.370013
  Sn  0.499996  0.666543  0.370321
  Sn  0.499995  0.999839  0.370218
  Sn  0.833311  0.333176  0.370024
  Sn  0.833332  0.666526  0.370329
  Sn  0.833343  0.999851  0.370206
  Sn  0.166652  0.166562  0.274619
```

Sn 0.166662 0.499944 0.274754
Sn 0.166650 0.833179 0.274703
Sn 0.499989 0.166557 0.274629
Sn 0.499988 0.499942 0.274755
Sn 0.499990 0.833179 0.274711
Sn 0.833329 0.166562 0.274620
Sn 0.833319 0.499944 0.274754
Sn 0.833327 0.833179 0.274703
Sn -0.000207 0.168211 0.716172
Sn 0.001194 0.500604 0.714155
Sn -0.000538 0.832577 0.715806
Sn 0.333491 0.168192 0.716181
Sn 0.333785 0.832614 0.715810
Sn 0.666622 0.167448 0.715731
Sn 0.666590 0.831692 0.715837
Sn 0.000866 0.332596 0.632194
Sn -0.000188 0.666320 0.633502
Sn -0.000068 0.999742 0.633471
Sn 0.332392 0.332611 0.632189
Sn 0.333490 0.666287 0.633509
Sn 0.333358 0.999736 0.633472
Sn 0.666612 0.332595 0.632774
Sn 0.666636 0.666181 0.633806
Sn 0.666625 0.999772 0.633439
Sn -0.000011 0.166542 0.365751
Sn -0.000041 0.499851 0.365884
Sn -0.000008 0.833143 0.365844
Sn 0.333314 0.166525 0.365761
Sn 0.333364 0.499868 0.365891
Sn 0.333314 0.833158 0.365845
Sn 0.666655 0.166530 0.365759
Sn 0.666649 0.499869 0.365889
Sn 0.666672 0.833157 0.365844
Sn -0.000012 0.333265 0.283479
Sn -0.000013 0.666544 0.283719
Sn -0.000011 0.999888 0.283610
Sn 0.333322 0.333228 0.283474
Sn 0.333330 0.666561 0.283727
Sn 0.333332 0.999872 0.283623
Sn 0.666661 0.333226 0.283473
Sn 0.666650 0.666562 0.283727
Sn 0.666646 0.999871 0.283625
Sn 0.166638 0.333187 0.544272

Sn	0.166714	0.666418	0.545014
Sn	0.166656	0.999766	0.544870
Sn	0.500296	0.333176	0.544280
Sn	0.500217	0.666503	0.544993
Sn	0.499976	0.999787	0.544856
Sn	0.833030	0.333177	0.544287
Sn	0.833180	0.666518	0.544991
Sn	0.833349	0.999777	0.544857
Sn	0.166653	0.166450	0.454347
Sn	0.166657	0.499814	0.454424
Sn	0.166663	0.833088	0.454446
Sn	0.499977	0.166442	0.454335
Sn	0.500169	0.499828	0.454538
Sn	0.499995	0.833097	0.454417
Sn	0.833343	0.166437	0.454333
Sn	0.833160	0.499826	0.454536
Sn	0.833349	0.833097	0.454439
Sn	-0.000023	0.166412	0.542083
Sn	-0.000555	0.499707	0.542136
Sn	0.000022	0.833062	0.542152
Sn	0.333324	0.166428	0.542082
Sn	0.333932	0.499681	0.542133
Sn	0.333370	0.833086	0.542146
Sn	0.666639	0.166430	0.542043
Sn	0.666679	0.499776	0.542491
Sn	0.666651	0.833134	0.542130
Sn	-0.000027	0.333075	0.456836
Sn	0.000001	0.666449	0.457299
Sn	0.000001	0.999787	0.457169
Sn	0.333338	0.333080	0.456852
Sn	0.333353	0.666451	0.457299
Sn	0.333323	0.999774	0.457166
Sn	0.666655	0.333061	0.456832
Sn	0.666670	0.666477	0.457284
Sn	0.666661	0.999767	0.457163
O	0.833167	0.556914	0.725794
O	0.500198	0.556924	0.725827
O	0.846901	0.434121	0.711156
O	0.486582	0.434126	0.711255
O	0.667009	0.360219	0.753680
O	0.666651	0.666099	0.755439
O	0.998090	0.335783	0.753986
O	0.335454	0.335793	0.753997

O	0.166626	0.105713	0.722477
O	0.166744	0.437653	0.720568
O	0.166677	0.770949	0.722431
O	0.499417	0.106669	0.722968
O	0.499638	0.770301	0.722728
O	0.833815	0.106676	0.722966
O	0.833580	0.770285	0.722755
O	0.166645	0.267174	0.629514
O	0.166626	0.601968	0.629929
O	0.166642	0.934558	0.631544
O	0.500515	0.267540	0.631062
O	0.500346	0.601409	0.631046
O	0.500108	0.934543	0.631533
O	0.832665	0.267531	0.631083
O	0.832927	0.601398	0.631039
O	0.833183	0.934528	0.631541
O	0.166643	0.101104	0.367701
O	0.166686	0.434382	0.367754
O	0.166731	0.767757	0.367827
O	0.499980	0.101100	0.367716
O	0.500010	0.434359	0.367770
O	0.499988	0.767765	0.367802
O	0.833339	0.101107	0.367704
O	0.833286	0.434372	0.367728
O	0.833260	0.767759	0.367826
O	0.166622	0.271532	0.276156
O	0.166694	0.604827	0.276433
O	0.166674	0.938157	0.276325
O	0.499983	0.271508	0.276199
O	0.499982	0.604843	0.276428
O	0.499988	0.938151	0.276337
O	0.833362	0.271531	0.276155
O	0.833287	0.604826	0.276437
O	0.833305	0.938158	0.276325
O	0.166702	0.228952	0.725299
O	0.166698	0.561288	0.723203
O	0.166605	0.894742	0.723262
O	0.503847	0.231189	0.725254
O	0.500644	0.894649	0.723318
O	0.829422	0.231214	0.725226
O	0.832536	0.894626	0.723257
O	0.166659	0.065471	0.631944
O	0.166631	0.398367	0.631288

O	0.166787	0.732114	0.632106
O	0.499915	0.065430	0.631811
O	0.500199	0.397509	0.632297
O	0.500102	0.731973	0.631978
O	0.833324	0.065390	0.631825
O	0.833071	0.397512	0.632322
O	0.833084	0.731980	0.631945
O	0.166709	0.231948	0.367726
O	0.166645	0.565284	0.367848
O	0.166641	0.898622	0.367755
O	0.499976	0.231946	0.367725
O	0.499999	0.565311	0.367854
O	0.499989	0.898591	0.367820
O	0.833269	0.231948	0.367717
O	0.833328	0.565299	0.367822
O	0.833347	0.898620	0.367759
O	0.166707	0.061615	0.276327
O	0.166713	0.395002	0.276287
O	0.166658	0.728255	0.276376
O	0.499991	0.061609	0.276333
O	0.499986	0.394986	0.276300
O	0.499983	0.728275	0.276426
O	0.833272	0.061614	0.276328
O	0.833271	0.395003	0.276289
O	0.833327	0.728255	0.276377
O	0.000222	0.666714	0.755002
O	-0.000244	0.001092	0.754859
O	0.333040	0.666687	0.755040
O	0.333424	0.001154	0.754877
O	0.666634	0.002198	0.754788
O	-0.000499	0.168069	0.664262
O	0.004175	0.503501	0.662470
O	-0.000168	0.833634	0.663924
O	0.333668	0.168099	0.664276
O	0.329167	0.503494	0.662461
O	0.333482	0.833760	0.663928
O	0.666647	0.167456	0.664009
O	0.666631	0.501402	0.665431
O	0.666646	0.833585	0.663926
O	-0.000008	0.333076	0.403188
O	0.000001	0.666559	0.403512
O	-0.000003	0.999893	0.403421
O	0.333359	0.333116	0.403206

O 0.333337 0.666561 0.403508
O 0.333290 0.999897 0.403406
O 0.666617 0.333113 0.403194
O 0.666651 0.666569 0.403501
O 0.666691 0.999893 0.403406
O -0.000009 0.166701 0.312777
O -0.000008 0.499845 0.312906
O -0.000014 0.833120 0.312857
O 0.333236 0.166694 0.312790
O 0.333311 0.499886 0.312916
O 0.333220 0.833151 0.312866
O 0.666722 0.166691 0.312789
O 0.666657 0.499888 0.312905
O 0.666755 0.833155 0.312866
O 0.006048 0.326081 0.684983
O -0.000593 0.663914 0.686546
O -0.000380 -0.000033 0.686477
O 0.327444 0.326122 0.684988
O 0.333900 0.663870 0.686564
O 0.333612 -0.000058 0.686478
O 0.666715 0.327272 0.687272
O 0.666671 0.664601 0.686903
O 0.666603 0.000649 0.686479
O 0.000015 0.166403 0.420360
O -0.000148 0.499712 0.420462
O 0.000019 0.833117 0.420434
O 0.333282 0.166478 0.420361
O 0.333439 0.499789 0.420464
O 0.333227 0.833099 0.420427
O 0.666681 0.166478 0.420358
O 0.666688 0.499795 0.420461
O 0.666758 0.833100 0.420423
O -0.000013 0.333104 0.335315
O 0.000002 0.666602 0.335557
O -0.000011 0.999891 0.335458
O 0.333255 0.333101 0.335309
O 0.333260 0.666559 0.335561
O 0.333350 0.999881 0.335463
O 0.666723 0.333094 0.335305
O 0.666713 0.666568 0.335557
O 0.666635 0.999878 0.335465
O -0.000011 0.166475 0.244288
O -0.000006 0.499970 0.244421

O	-0.000013	0.833199	0.244367
O	0.333332	0.166549	0.244291
O	0.333328	0.499960	0.244423
O	0.333330	0.833161	0.244373
O	0.666651	0.166554	0.244293
O	0.666651	0.499960	0.244425
O	0.666643	0.833158	0.244373
O	0.166675	0.101863	0.543150
O	0.166671	0.434987	0.542920
O	0.166785	0.768560	0.543045
O	0.499975	0.101855	0.543102
O	0.500568	0.434844	0.542629
O	0.500047	0.768615	0.543051
O	0.833292	0.101840	0.543113
O	0.832763	0.434878	0.542631
O	0.833239	0.768634	0.543030
O	0.166656	0.268420	0.455909
O	0.166656	0.601799	0.456176
O	0.166686	0.935169	0.456135
O	0.499974	0.268398	0.455977
O	0.500026	0.601849	0.456209
O	0.499977	0.935174	0.456104
O	0.833346	0.268402	0.455898
O	0.833344	0.601839	0.456171
O	0.833333	0.935173	0.456126
O	0.166691	0.231127	0.543164
O	0.166717	0.564248	0.543576
O	0.166681	0.897849	0.543178
O	0.499969	0.231119	0.543184
O	0.500448	0.564449	0.543205
O	0.499968	0.897881	0.543174
O	0.833301	0.231093	0.543192
O	0.832944	0.564468	0.543203
O	0.833369	0.897879	0.543177
O	0.166716	0.064465	0.456160
O	0.166641	0.397715	0.456001
O	0.166695	0.731121	0.456244
O	0.499986	0.064459	0.456155
O	0.500012	0.397688	0.456069
O	0.499977	0.731154	0.456238
O	0.833277	0.064458	0.456161
O	0.833301	0.397691	0.456008
O	0.833352	0.731138	0.456227

O -0.000537 0.333360 0.578228
O -0.000288 0.667379 0.578845
O 0.000021 0.000094 0.578931
O 0.333815 0.333390 0.578215
O 0.333675 0.667267 0.578841
O 0.333283 0.000086 0.578941
O 0.666676 0.333282 0.578450
O 0.666723 0.667082 0.578950
O 0.666665 0.000121 0.578918
O -0.000007 0.166554 0.488744
O 0.000102 0.499845 0.488800
O 0.000009 0.833249 0.488785
O 0.333303 0.166552 0.488737
O 0.333218 0.499811 0.488798
O 0.333364 0.833258 0.488773
O 0.666670 0.166542 0.488719
O 0.666662 0.499842 0.488875
O 0.666644 0.833307 0.488763
O -0.000203 0.165855 0.595988
O -0.003103 0.498463 0.595575
O 0.000010 0.833162 0.595949
O 0.333454 0.165874 0.595992
O 0.336395 0.498415 0.595570
O 0.333345 0.833190 0.595955
O 0.666610 0.166114 0.595919
O 0.666672 0.498500 0.596428
O 0.666648 0.833252 0.595930
O -0.000069 0.332704 0.510116
O 0.000066 0.666288 0.510591
O 0.000028 0.999904 0.510537
O 0.333318 0.332772 0.510130
O 0.333343 0.666249 0.510594
O 0.333259 0.999859 0.510541
O 0.666742 0.332485 0.510119
O 0.666708 0.666572 0.510567
O 0.666697 0.999830 0.510533
O 0.667277 0.431778 0.765322

Structure 14: S_2-type configuration of O on $SnO_2(100)$ surface.

```
_cell_length_a   9.42441600
_cell_length_b   14.11538000
_cell_length_c   32.11538000
_cell_angle_alpha   90.00000000
_cell_angle_beta   90.00000000
_cell_angle_gamma   90.00000000
loop_
 _atom_site_type_symbol
 _atom_site_fract_x
 _atom_site_fract_y
 _atom_site_fract_z
  Sn  0.002466  0.662353  0.684198
  Sn  -0.000062  0.333371  0.536496
  Sn  0.323876  0.319413  0.689529
  Sn  0.332311  0.666713  0.536049
  Sn  0.336249  0.661236  0.684481
  Sn  0.333700  0.333075  0.536797
  Sn  0.668284  0.667241  0.535813
  Sn  0.669133  0.665478  0.684281
  Sn  0.666600  0.332882  0.536681
  Sn  0.671513  0.318307  0.685773
  Sn  -0.002546  0.326281  0.683818
  Sn  0.166637  0.167062  0.609952
  Sn  0.167161  0.832743  0.609803
  Sn  0.167067  0.496886  0.611136
  Sn  0.499116  0.166724  0.610222
  Sn  0.500347  0.832889  0.609239
  Sn  0.501482  0.505665  0.608606
  Sn  0.833450  0.164628  0.608898
  Sn  0.833351  0.498879  0.611018
  Sn  0.166670  0.161714  0.315281
  Sn  0.166677  0.494928  0.315289
  Sn  0.166695  0.828548  0.315354
  Sn  0.500022  0.161745  0.315279
  Sn  0.500036  0.494899  0.315257
  Sn  0.500029  0.828504  0.315358
  Sn  0.833370  0.161707  0.315288
  Sn  0.833375  0.494936  0.315299
  Sn  0.833373  0.828558  0.315364
  Sn  0.166838  0.166518  0.462999
  Sn  0.166842  0.499424  0.463000
```

Sn	0.166774	0.832668	0.462891
Sn	0.499993	0.166446	0.462981
Sn	0.500171	0.499576	0.462960
Sn	0.500027	0.832954	0.462829
Sn	0.833422	0.166484	0.463006
Sn	0.833468	0.499428	0.462954
Sn	0.833739	0.832669	0.462815
Sn	0.834050	0.833378	0.609550
Sn	0.000073	0.332860	0.389529
Sn	0.000144	0.666121	0.389468
Sn	0.000087	0.999744	0.389613
Sn	0.333405	0.332914	0.389522
Sn	0.333410	0.666087	0.389472
Sn	0.333362	0.999804	0.389570
Sn	0.666715	0.332897	0.389527
Sn	0.666799	0.666106	0.389488
Sn	0.666735	0.999822	0.389569
Sn	0.000522	0.664568	0.536912
Sn	0.000507	0.999323	0.536145
Sn	0.333322	0.999772	0.536384
Sn	0.666676	0.999549	0.536142
Sn	0.000092	0.995301	0.683866
Sn	0.333530	0.994574	0.683830
Sn	0.666646	0.994789	0.683793
O	0.336383	0.432836	0.583256
O	0.333031	0.101505	0.581765
O	0.333043	0.767725	0.581097
O	0.665536	0.100411	0.581333
O	0.667912	0.768064	0.581012
O	0.665431	0.433300	0.583029
O	0.999093	0.433504	0.582239
O	0.000638	0.566211	0.638020
O	0.332291	0.236157	0.637964
O	0.333867	0.900424	0.637073
O	0.333473	0.564874	0.491146
O	0.334298	0.569527	0.636289
O	0.668211	0.570864	0.637172
O	0.668438	0.233985	0.636128
O	0.997908	0.231954	0.637133
O	0.167182	0.073390	0.657383
O	0.168147	0.739824	0.657559
O	0.167405	0.398901	0.508648
O	0.159879	0.400375	0.657745

O	0.499980	0.071863	0.656802
O	0.500739	0.738154	0.655582
O	0.500242	0.399074	0.508701
O	0.521975	0.412318	0.658742
O	0.832630	0.072324	0.656848
O	0.835609	0.741065	0.657323
O	0.831617	0.403723	0.658739
O	0.167585	0.599054	0.564863
O	0.168976	0.619562	0.716056
O	0.166630	0.268169	0.564770
O	0.154616	0.279493	0.718602
O	0.500836	0.602373	0.563499
O	0.504135	0.632612	0.718573
O	0.499985	0.267417	0.564792
O	0.502956	0.265292	0.711543
O	0.832837	0.599779	0.564747
O	0.835461	0.622225	0.716229
O	0.833435	0.267308	0.564326
O	0.166705	0.067200	0.361954
O	0.166730	0.400507	0.362014
O	0.166812	0.733482	0.361840
O	0.500031	0.067204	0.361951
O	0.500055	0.400423	0.361965
O	0.500096	0.733579	0.361908
O	0.833412	0.067200	0.361966
O	0.833384	0.400499	0.362030
O	0.833396	0.733544	0.361879
O	0.167056	0.064863	0.507948
O	0.165657	0.730817	0.507967
O	0.499763	0.064762	0.507923
O	0.500062	0.731603	0.507719
O	0.833553	0.064941	0.507999
O	0.832715	0.398722	0.508465
O	0.835282	0.731038	0.507906
O	0.166803	0.267322	0.417629
O	0.166768	0.600295	0.417595
O	0.166894	0.934064	0.417664
O	0.500049	0.267372	0.417643
O	0.500095	0.600277	0.417538
O	0.500067	0.934075	0.417650
O	0.833364	0.267308	0.417645
O	0.833555	0.600310	0.417574
O	0.833285	0.934076	0.417664

O	0.167055	0.934077	0.564447
O	0.500012	0.934303	0.564354
O	0.833650	0.934468	0.564398
O	0.166730	0.953423	0.715929
O	0.500192	0.953756	0.716073
O	0.836864	0.280616	0.717462
O	0.833418	0.954175	0.716079
O	0.000011	0.120284	0.283148
O	0.000005	0.453634	0.283133
O	0.000019	0.787540	0.283124
O	0.333337	0.120404	0.283124
O	0.333287	0.453554	0.283115
O	0.333351	0.787457	0.283134
O	0.666705	0.120402	0.283126
O	0.666772	0.453549	0.283121
O	0.666726	0.787459	0.283145
O	0.000109	0.101089	0.434773
O	0.000181	0.433637	0.434934
O	0.000299	0.767579	0.434503
O	0.333387	0.101170	0.434714
O	0.333460	0.433842	0.434895
O	0.333325	0.767364	0.434627
O	0.666735	0.101039	0.434764
O	0.666725	0.433871	0.434854
O	0.666913	0.767356	0.434674
O	0.001102	0.100357	0.581251
O	0.000591	0.766864	0.581728
O	0.000039	0.239653	0.341947
O	0.000064	0.572684	0.342007
O	0.000055	0.906177	0.342162
O	0.333293	0.239544	0.341998
O	0.333391	0.572690	0.341978
O	0.333394	0.906196	0.342143
O	0.666789	0.239538	0.342010
O	0.666755	0.572701	0.341979
O	0.666684	0.906191	0.342152
O	0.000252	0.232052	0.491231
O	0.000165	0.564266	0.491271
O	0.000239	0.898139	0.491030
O	0.333479	0.231971	0.491342
O	0.333194	0.897996	0.491193
O	0.666529	0.231844	0.491261
O	0.666999	0.565059	0.491048

O	0.667133	0.897895	0.491148
O	0.000740	0.901171	0.637017
O	0.666837	0.900119	0.637179
O	0.426791	0.443720	0.694017

Structure 15: L-type configuration of O on SnO_2(101) surface.

```
_cell_length_a   16.97242900
_cell_length_b   14.11702300
_cell_length_c   33.67701300
_cell_angle_alpha   90.00000000
_cell_angle_beta   90.00000000
_cell_angle_gamma   90.00000000
loop_
 _atom_site_type_symbol
 _atom_site_fract_x
 _atom_site_fract_y
 _atom_site_fract_z
  Sn  0.377423  0.178678  0.687674
  Sn  0.390076  0.497297  0.694573
  Sn  0.489585  0.499943  0.611991
  Sn  0.322669  0.336542  0.611824
  Sn  0.216685  0.321046  0.688364
  Sn  0.556981  0.332256  0.687878
  Sn  0.550397  0.651144  0.689306
  Sn  0.156958  0.163044  0.612583
  Sn  0.157083  0.498114  0.612450
  Sn  0.158494  0.828048  0.613342
  Sn  0.491081  0.166741  0.613300
  Sn  0.490656  0.830941  0.613258
  Sn  0.824211  0.163908  0.612898
  Sn  0.824916  0.498129  0.612426
  Sn  0.823819  0.831370  0.612729
  Sn  0.029582  0.165312  0.457256
  Sn  0.029583  0.499121  0.457031
  Sn  0.029342  0.832377  0.457143
  Sn  0.362835  0.165649  0.457277
  Sn  0.362874  0.499290  0.457113
  Sn  0.362797  0.832664  0.457409
  Sn  0.696043  0.165745  0.457154
  Sn  0.696349  0.499165  0.457062
  Sn  0.696021  0.832623  0.457257
  Sn  0.238738  0.153219  0.302714
  Sn  0.238818  0.486667  0.302640
  Sn  0.238810  0.820018  0.302677
  Sn  0.572039  0.153261  0.302689
  Sn  0.572114  0.486698  0.302657
  Sn  0.572057  0.819998  0.302708
```

Sn	0.905350	0.153176	0.302635
Sn	0.905464	0.486603	0.302621
Sn	0.905390	0.819962	0.302638
Sn	0.050316	0.179928	0.689184
Sn	0.050941	0.513281	0.689191
Sn	0.051161	0.845297	0.689294
Sn	0.383718	0.845825	0.689454
Sn	0.715579	0.182745	0.688989
Sn	0.720401	0.515587	0.689091
Sn	0.717343	0.846016	0.689471
Sn	0.259461	0.167113	0.534659
Sn	0.259638	0.501262	0.534520
Sn	0.260033	0.832964	0.535295
Sn	0.592950	0.167847	0.534769
Sn	0.593544	0.501080	0.534540
Sn	0.592934	0.834601	0.534954
Sn	0.926659	0.167106	0.534812
Sn	0.926695	0.500956	0.534499
Sn	0.926280	0.834210	0.534731
Sn	0.131957	0.168928	0.379245
Sn	0.132005	0.502540	0.379111
Sn	0.131906	0.835819	0.379173
Sn	0.465261	0.169138	0.379231
Sn	0.465335	0.502624	0.379169
Sn	0.465236	0.835836	0.379290
Sn	0.798557	0.169043	0.379153
Sn	0.798681	0.502489	0.379112
Sn	0.798501	0.835841	0.379186
Sn	0.215931	0.984990	0.689242
Sn	0.220689	0.647805	0.690359
Sn	0.550644	0.987136	0.690414
Sn	0.883732	0.986363	0.689396
Sn	0.883994	0.320301	0.689152
Sn	0.885230	0.654374	0.689162
Sn	0.093380	0.997938	0.534892
Sn	0.093156	0.332322	0.534635
Sn	0.093027	0.665854	0.534547
Sn	0.426824	0.999683	0.535176
Sn	0.426421	0.332893	0.534590
Sn	0.426156	0.665996	0.534927
Sn	0.759503	0.999389	0.534851
Sn	0.760590	0.332427	0.534353
Sn	0.759740	0.666117	0.534660

Sn	0.298660	0.330839	0.379189
Sn	0.298641	0.664322	0.379202
Sn	0.298652	0.997446	0.379276
Sn	0.631966	0.330870	0.379150
Sn	0.631882	0.664272	0.379235
Sn	0.631843	0.997475	0.379228
Sn	0.965435	0.330661	0.379095
Sn	0.965244	0.664218	0.379099
Sn	0.965179	0.997427	0.379187
Sn	0.323464	0.001214	0.612445
Sn	0.326247	0.665166	0.614306
Sn	0.656885	0.003227	0.613079
Sn	0.658219	0.336890	0.611502
Sn	0.657720	0.669898	0.612810
Sn	0.990916	0.001588	0.612911
Sn	0.990903	0.335750	0.612661
Sn	0.990866	0.669174	0.612506
Sn	0.196270	0.000287	0.457306
Sn	0.196123	0.334108	0.457105
Sn	0.196158	0.667770	0.457114
Sn	0.529356	0.000819	0.457303
Sn	0.529502	0.334311	0.457116
Sn	0.529423	0.667765	0.457327
Sn	0.862674	0.000846	0.457221
Sn	0.863150	0.333961	0.456989
Sn	0.862775	0.667666	0.457095
Sn	0.072056	0.013318	0.302670
Sn	0.072148	0.346625	0.302623
Sn	0.072134	0.680118	0.302610
Sn	0.405411	0.013313	0.302731
Sn	0.405443	0.346712	0.302681
Sn	0.405448	0.680118	0.302682
Sn	0.738676	0.013292	0.302669
Sn	0.738741	0.346699	0.302636
Sn	0.738714	0.680073	0.302662
O	0.452884	0.295484	0.714746
O	0.459340	0.601078	0.715508
O	0.227261	0.265385	0.633782
O	0.562231	0.269885	0.634446
O	0.313803	0.393911	0.666819
O	0.655389	0.399555	0.667325
O	0.420140	0.397807	0.588367
O	0.481922	0.112976	0.672742

O	0.490698	0.437225	0.665760
O	0.288691	0.229057	0.715416
O	0.621079	0.236313	0.714634
O	0.293122	0.557105	0.714915
O	0.625091	0.560196	0.714163
O	0.394220	0.233536	0.634133
O	0.227231	0.600952	0.634301
O	0.227389	0.933850	0.634225
O	0.561084	0.603684	0.633490
O	0.558941	0.938536	0.634721
O	0.893916	0.268571	0.634072
O	0.894551	0.602155	0.633999
O	0.893622	0.935431	0.634110
O	0.099414	0.269140	0.480429
O	0.099415	0.602798	0.480320
O	0.099494	0.935463	0.480599
O	0.432859	0.269219	0.480544
O	0.432679	0.602993	0.480539
O	0.432847	0.936160	0.480877
O	0.766337	0.269230	0.480295
O	0.766074	0.602980	0.480413
O	0.765835	0.936251	0.480593
O	0.306661	0.269035	0.323116
O	0.306752	0.602464	0.323082
O	0.306704	0.935644	0.323189
O	0.639918	0.269073	0.323073
O	0.640002	0.602433	0.323101
O	0.639915	0.935663	0.323128
O	0.973300	0.268953	0.323035
O	0.973384	0.602426	0.323002
O	0.973286	0.935613	0.323093
O	0.124031	0.269926	0.714436
O	0.125135	0.600857	0.715226
O	0.123159	0.935688	0.715400
O	0.455857	0.938876	0.714785
O	0.789265	0.271496	0.714624
O	0.791914	0.605759	0.715218
O	0.790261	0.936262	0.715028
O	0.329420	0.269753	0.558152
O	0.330405	0.602125	0.559004
O	0.330066	0.936714	0.558472
O	0.663211	0.270639	0.557827
O	0.662906	0.604360	0.558328

O	0.662215	0.938005	0.558608
O	0.996162	0.270163	0.558333
O	0.996112	0.603727	0.558163
O	0.996168	0.936457	0.558463
O	0.202040	0.269115	0.402936
O	0.202004	0.602720	0.402925
O	0.202097	0.935648	0.403059
O	0.535341	0.269265	0.402949
O	0.535246	0.602808	0.403032
O	0.535269	0.935881	0.403070
O	0.868765	0.269080	0.402851
O	0.868581	0.602734	0.402869
O	0.868547	0.935848	0.402987
O	0.087201	0.063338	0.588932
O	0.087269	0.397407	0.588767
O	0.087449	0.730213	0.588843
O	0.421295	0.064645	0.590041
O	0.421665	0.729030	0.589686
O	0.753623	0.064291	0.589182
O	0.755032	0.397946	0.588417
O	0.754042	0.731474	0.588858
O	0.293234	0.062681	0.433699
O	0.293060	0.396430	0.433473
O	0.293185	0.729894	0.433579
O	0.626305	0.062962	0.433619
O	0.626534	0.396372	0.433475
O	0.626370	0.729869	0.433622
O	0.959661	0.062854	0.433532
O	0.959962	0.396216	0.433397
O	0.959683	0.729793	0.433449
O	0.165640	0.063190	0.277095
O	0.165687	0.396622	0.277057
O	0.165741	0.730003	0.277049
O	0.498924	0.063238	0.277113
O	0.498984	0.396654	0.277086
O	0.498998	0.729985	0.277081
O	0.832193	0.063229	0.277030
O	0.832292	0.396613	0.277044
O	0.832280	0.729936	0.277045
O	0.314348	0.063799	0.668096
O	0.316244	0.730336	0.669733
O	0.647347	0.067063	0.669627
O	0.649710	0.730555	0.669384

O	0.982106	0.064355	0.668927
O	0.982956	0.397376	0.668767
O	0.982988	0.730799	0.668655
O	0.189784	0.063313	0.511479
O	0.189679	0.397360	0.511174
O	0.189862	0.730413	0.511398
O	0.523281	0.064136	0.511706
O	0.523287	0.397470	0.511216
O	0.523082	0.731000	0.511626
O	0.856273	0.063997	0.511454
O	0.857003	0.397187	0.511159
O	0.856347	0.730900	0.511327
O	0.061965	0.064758	0.357842
O	0.062095	0.398118	0.357793
O	0.061999	0.731551	0.357786
O	0.395342	0.064858	0.357912
O	0.395390	0.398223	0.357854
O	0.395382	0.731600	0.357877
O	0.728574	0.064812	0.357845
O	0.728705	0.398197	0.357814
O	0.728601	0.731565	0.357844
O	0.148625	0.101034	0.668868
O	0.148617	0.435130	0.668217
O	0.149771	0.767231	0.669334
O	0.483004	0.769958	0.669255
O	0.814812	0.102776	0.669048
O	0.818057	0.437289	0.668880
O	0.816174	0.769530	0.668584
O	0.023289	0.102019	0.511489
O	0.023199	0.435911	0.511217
O	0.022967	0.769309	0.511332
O	0.356741	0.102724	0.511674
O	0.356137	0.435805	0.511119
O	0.356566	0.769110	0.511649
O	0.689689	0.102994	0.511526
O	0.690289	0.436120	0.511221
O	0.689712	0.769897	0.511533
O	0.228712	0.101679	0.357906
O	0.228711	0.435122	0.357793
O	0.228690	0.768522	0.357839
O	0.561983	0.101791	0.357892
O	0.562039	0.435199	0.357829
O	0.561972	0.768544	0.357893

O	0.895268	0.101683	0.357818
O	0.895417	0.435059	0.357780
O	0.895270	0.768512	0.357806
O	0.253740	0.101397	0.588684
O	0.252568	0.435467	0.588159
O	0.255647	0.767275	0.589520
O	0.587294	0.104196	0.589603
O	0.587374	0.436719	0.588400
O	0.588098	0.771291	0.589486
O	0.920802	0.102164	0.589076
O	0.921059	0.435972	0.588737
O	0.920648	0.769599	0.588971
O	0.126517	0.103155	0.433568
O	0.126476	0.436946	0.433396
O	0.126358	0.770314	0.433495
O	0.459809	0.103599	0.433651
O	0.459772	0.437017	0.433467
O	0.459765	0.770576	0.433716
O	0.792961	0.103517	0.433512
O	0.793322	0.436948	0.433436
O	0.793011	0.770476	0.433561
O	0.332278	0.103281	0.277120
O	0.332348	0.436716	0.277051
O	0.332400	0.770220	0.277085
O	0.665571	0.103315	0.277080
O	0.665636	0.436734	0.277042
O	0.665606	0.770138	0.277079
O	0.998919	0.103259	0.277055
O	0.998993	0.436614	0.277021
O	0.999012	0.770177	0.277043
O	0.060399	0.230932	0.633980
O	0.060487	0.564911	0.633990
O	0.061322	0.897507	0.634209
O	0.394329	0.560658	0.636877
O	0.393738	0.898228	0.634364
O	0.727082	0.232048	0.633680
O	0.729049	0.566460	0.633883
O	0.726561	0.898949	0.634404
O	0.265945	0.230194	0.480455
O	0.266244	0.564423	0.480456
O	0.266157	0.896874	0.480905
O	0.599260	0.230515	0.480465
O	0.599628	0.564146	0.480424

O	0.599319	0.897574	0.480689
O	0.932815	0.230235	0.480507
O	0.932807	0.564040	0.480309
O	0.932662	0.897327	0.480513
O	0.139980	0.230807	0.323108
O	0.140056	0.564393	0.323044
O	0.140040	0.897644	0.323113
O	0.473269	0.230897	0.323111
O	0.473360	0.564399	0.323098
O	0.473332	0.897661	0.323177
O	0.806570	0.230863	0.323058
O	0.806690	0.564340	0.323049
O	0.806592	0.897625	0.323084
O	0.290893	0.897974	0.715041
O	0.624095	0.897068	0.715223
O	0.956666	0.230077	0.714872
O	0.957715	0.563789	0.714792
O	0.956946	0.895524	0.714480
O	0.162593	0.229176	0.558252
O	0.162661	0.563408	0.558125
O	0.163324	0.895196	0.558863
O	0.496351	0.230392	0.558653
O	0.496353	0.563575	0.557833
O	0.495985	0.896605	0.558862
O	0.829602	0.229406	0.558398
O	0.829854	0.563405	0.558133
O	0.829296	0.896742	0.558427
O	0.035344	0.230433	0.402980
O	0.035317	0.564137	0.402847
O	0.035257	0.897378	0.402960
O	0.368667	0.230659	0.403033
O	0.368674	0.564211	0.402935
O	0.368705	0.897515	0.403143
O	0.701943	0.230665	0.402930
O	0.702045	0.564161	0.402909
O	0.701852	0.897433	0.403009
O	0.418489	0.382320	0.733365
